# A Novel Clade of Unique Eukaryotic Ribonucleotide Reductase R2 Subunits is Exclusive to Apicomplexan Parasites

**DOI:** 10.1007/s00239-013-9583-y

**Published:** 2013-09-18

**Authors:** James B. Munro, Christopher G. Jacob, Joana C. Silva

**Affiliations:** 1 Department of Microbiology and Immunology, University of Maryland School of Medicine, Baltimore, MD 21201 USA; 2 Institute for Genome Sciences, University of Maryland School of Medicine, 801 W. Baltimore Street, 6th Floor, Baltimore, MD 21201 USA; 3 Howard Hughes Medical Institute, Center of Vaccine Development, University of Maryland School of Medicine, Baltimore, MD 21201 USA

**Keywords:** Ribonucleotide reductase, RNR, Apicomplexa, Structure-based amino acid alignment, Paralog

## Abstract

Apicomplexa are protist parasites of tremendous medical and economic importance, causing millions of deaths and billions of dollars in losses each year. Apicomplexan-related diseases may be controlled via inhibition of essential enzymes. Ribonucleotide reductase (RNR) provides the only de novo means of synthesizing deoxyribonucleotides, essential precursors for DNA replication and repair. RNR has long been the target of antibacterial and antiviral therapeutics. However, targeting this ubiquitous protein in eukaryotic pathogens may be problematic unless these proteins differ significantly from that of their respective host. The typical eukaryotic RNR enzymes belong to class Ia, and the holoenzyme consists minimally of two R1 and two R2 subunits (α_2_β_2_). We generated a comparative, annotated, structure-based, multiple-sequence alignment of R2 subunits, identified a clade of R2 subunits unique to Apicomplexa, and determined its phylogenetic position. Our analyses revealed that the apicomplexan-specific sequences share characteristics with both class I R2 and R2lox proteins. The putative radical-harboring residue, essential for the reduction reaction by class Ia R2-containing holoenzymes, was not conserved within this group. Phylogenetic analyses suggest that class Ia subunits are not monophyletic and consistently placed the apicomplexan-specific clade sister to the remaining class Ia eukaryote R2 subunits. Our research suggests that the novel apicomplexan R2 subunit may be a promising candidate for chemotherapeutic-induced inhibition as it differs greatly from known eukaryotic host RNRs and may be specifically targeted.

**Electronic supplementary material:**

The online version of this article (doi:10.1007/s00239-013-9583-y) contains supplementary material, which is available to authorized users.

## Introduction

The phylum Apicomplexa consists of more than 4,000 described species nearly all of which are obligate, intracellular parasites (Adl et al. 2005; Levine 1988). Many species of Apicomplexa are of medical, agricultural, and economic importance and their adverse impact on human society cannot be overstated. *Babesia*, *Theileria*, *Toxoplasma*, *Cryptosporidium*, and *Plasmodium* are causative agents of babesiosis (hemolytic anemia), theileriosis and East Coast fever, toxoplasmosis, cryptosporidiosis, and malaria, respectively. With increasing incidence of multiple drug resistance, the development of new chemotherapeutic and prophylactic antimalarial (Bustamante et al. 2009; Takala and Plowe 2009) and antiprotozoan (de Azevedo and Soares 2009; da Cunha et al. 2010) drugs and vaccines remains a priority.

The availability of genome sequences from several related species and isolates of Apicomplexa have facilitated the identification of potential drug targets (Winzeler 2008). Essential enzymes are obvious choices, since their inhibition will kill the pathogen. One such example is the ubiquitous and vital enzyme ribonucleotide reductase (RNR) (EC 1.17.4.1). RNR inhibitors have been extensively explored for their utility in cancer chemotherapy (Cerqueira et al. 2007), as antiviral (Moss et al. 1993; Szekeres et al. 1997) and antibacterial agents (Torrents and Sjöberg 2010; Lou and Zhang 2010), and for their potential use in the control of Apicomplexa (Akiyoshi et al. 2002; Hyde 2007; Rubin et al. 1993) and other eukaryotic pathogens (Dormeyer et al. 1997; Ingram and Kinnaird 1999).

RNR provides the only de novo means of generating deoxyribonucleotide diphosphates (dNDPs), an essential step in synthesizing the building blocks for DNA replication and repair (Jordan and Reichard 1998). Synthesis of dNDPs by RNR relies on the use of radical chemistry to catalyze the reduction of the 2′-hydroxyl of a ribonucleotide to hydrogen (Harder 1993). RNR is also essential in maintaining a balanced pool of DNA precursors (Herrick and Sclavi 2007). Deviations in the dNTP pool, both in terms of asymmetry in nucleotide ratios and in terms of dNTP pool expansion, can lead to a loss of DNA replication fidelity and to an increase in mutation and disease (Mathews 2006; Wheeler et al. 2005).

**Fig. 1:**
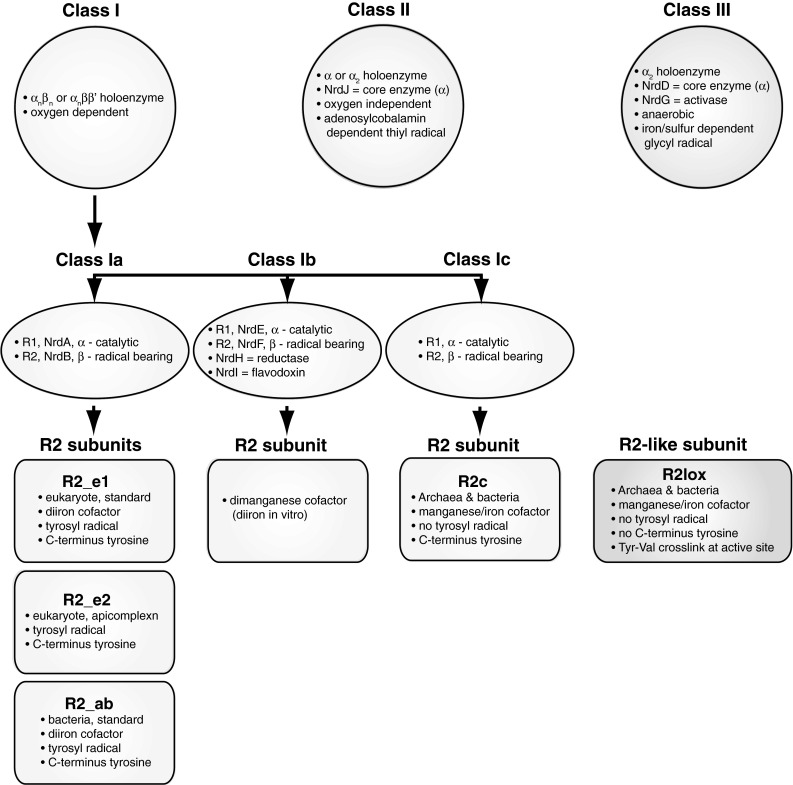
Schematic of RNR classification classification based on enzyme structure and chemistry, with the division of RNR into class I, II, and III, and the division of class I into Ia, Ib, and Ic. Further division of the class Ia R2 subunits follows our phylogenetic analyses

RNRs have been divided into three classes on the basis of their metallocofactor requirements, dependency/reaction with oxygen, and means by which the protein radical is generated (Eklund et al. 2001) (Fig. 1). Typical class I RNRs (i.e., class Ia) are characterized by their oxygen requirement to form a stable tyrosyl radical using a diiron center. In contrast, class II RNRs are indifferent to oxygen and form a thiyl radical via adenosylcobalamin and class III RNRs are anaerobic and form a glycyl radical using an iron–sulfur center in the presence of S-adenosylmethionine and reduced flavodoxin (Nordlund and Reichard 2006). Class I RNRs have been subdivided into classes Ia, Ib, and Ic (Fig. 1). Standard class Ia enzymes utilize the characteristic diiron cofactor, which reacts with oxygen to generate a stable tyrosyl radical. In contrast, class Ib enzymes utilize a dimanganese/tyrosyl cofactor and class Ic enzymes, which lack the tyrosyl radical and diiron site, utilize a manganese/iron metal center (Cotruvo and Stubbe 2011).

Class I proteins consist of two different subunits that form an α_n_β_2_ structure, where the number of subunits (*n*) can be 2 or 6 (Rofougaran et al. 2006). Class I small subunit β is the focus of this work and is further detailed below. The class Ia α component is a homopolymer formed by large subunits, also termed R1 subunits. The β component is typically a homopolymer composed of two small subunits termed R2. However, multiple, distinct copies of the R2 subunit gene are known to occur in many organisms, which can lead to the formation of ββ′ heterodimers, or ββ and β′β′ homodimers. These secondary R2 polypeptides are usually shorter as they lack amino acid residues from the N-terminus (Roa et al. 2009; Tanaka et al. 2000). While β′ cannot assemble a diiron/tyrosyl cofactor, heterodimeric ββ′ RNRs perform one-electron oxidation by generating a temporary, stable tyrosyl radical (Sjöberg 1997; Stubbe et al. 2003).

Class Ic was established to include the R2c proteins, which were typified by the *Chlamydia trachomatis* CtR2c protein (Högbom et al. 2004). Described as being R2-homologs and R2c-like, the R2lox (i.e., R2-like ligand-binding oxidases) proteins were subsequently documented and typified by the *Mycobacterium tuberculosis* Rv0233 protein (Andersson and Högbom 2009). However, the C-terminus structure of R2lox suggests that these proteins do not interact with R1 subunits, and as such, they are not believed to be involved in ribonucleotide reduction (Andersson and Högbom 2009; Högbom 2010). Both R2c and R2lox proteins utilize a manganese/iron-carboxylate cofactor and lack the characteristic tyrosine used in radical formation; however, while the R2c protein accomplishes one-electron oxidation, R2lox proteins may potentially accomplish two-electron oxidation and have a unique tyrosine–valine cross-link at the active site (Högbom 2010; Jiang et al. 2007; Voevodskaya et al. 2007). The RNR R2 subunits, R2lox proteins, and bacterial multicomponent monooxygenases (BMMs) are believed to be homologous, although the evolutionary relationship among them is still to be determined (Andersson and Högbom 2009).

Class I RNRs are found in eukaryotes (typically class Ia), bacteria (almost equally represented by classes Ia and Ib), bacteriophages, and viruses, with a limited distribution in Archaea, while class II and III RNRs are typical of Archaea and bacteria, with limited distribution in eukaryotes (Lundin et al. 2009). With respect to Apicomplexa, the large and small subunits (termed NrdA and NrdB proteins, respectively) were first identified and characterized in *Plasmodium falciparum* (Chakrabarti et al. 1993). A second copy of the small subunit gene (PfR4) was later documented in *P. falciparum* and found to be highly divergent from the standard PfR2 (a typical NrdB protein) (Bracchi-Ricard et al. 2005). The recent completion of several Apicomplexa genome projects has revealed the presence of two NrdB homologous proteins in several of these organisms, a subset of which are represented in the Ribonucleotide Reductase database (RNRdb) (Lundin et al. 2009).

In order to characterize all R2 subunits from apicomplexan parasites and define their phylogenetic position relative to their eukaryotic homologs, we identify all small RNR subunits present in publically available apicomplexan genomes, including several which were not available in RNRdb or were incomplete, and determine their evolutionary history in the wider context of class I RNR small subunits. We produced a structure-based, highly curated amino acid alignment of apicomplexan-specific R2 RNR subunits, standard R2 RNR subunits, R2c RNR subunits, and R2lox proteins, represented by Archaea, bacteria, and eukaryotes. To facilitate interpretation, the positions in this alignment were cross-referenced with those from seminal functional studies. The phylogenetic relationships among these sequences were then inferred using maximum likelihood and Bayesian optimality criteria. Additionally, we provide an extensive sequence comparison study comprising the class Ia R2, class Ic R2c, R2lox, and all apicomplexan-specific proteins, in order to assess the potential functionality of the two different apicomplexan R2 subunits.

## Materials and Methods

### Data Collection and Alignment

A total of 121 unique sequences were obtained by querying public databases, including the RNRdb, NCBI Protein Data Bank, the Broad Institute, and Eukaryotic Pathogen Database Resources (EuPathDB). Redundant sequences were removed. The *T. annulata* and *T. parva* PfR4 (R2_e2) homologs appeared truncated so the Web-based comparative genome visualization tool Sybil (Crabtree et al. 2007) was used to download genomic sequence flanking the annotated genes. From these data, the conserved 3′ sequences were identified. Supplemental Table S1 provides a list of the sequences used in this study, their taxonomic origin, and their unique identifier number (NCBI or otherwise). These sequences represented class Ia and Ic RNR R2s from Archaea, bacteria, and eukaryotes as well as R2lox protein homologs from Archaea and bacteria. Class Ic and R2lox sequences were included because like the apicomplexan-specific R2s, the R2c and R2lox proteins lack a radical-forming tyrosyl.

The majority of the sequences in our matrix lacked structural data. Thus, sequence searches using BLAST were employed to find “best-matching” structures in the RCSB Protein Data Bank (Berman et al. 2000). Redundant chains were removed, and unique entries were pooled. These sequences were aligned using the native combinatorial extension (CE) (Shindyalov and Bourne 1998) as implemented in the Java application STRAP version 1.0 (Gille and Frömmel 2001) to produce an alignment based on α-carbon positions. The resulting structure-based alignment was then employed as a template for the multiple-sequence alignment of our 121 sequences using ClustalW2 (Larkin et al. 2007), as implemented in STRAP.

Minimal manual correction was used to ensure that positional homology was retained for functionally and structurally conserved residues. All manual adjustments are described in the alignment document (Supplemental Fig. S1). Further curation of the alignment included assignment of *S*. *cerevisiae* Y2 coordinates to the alignment and the identification of conserved positions and functional residues (Andersson and Högbom 2009; Högbom et al. 2004; Högbom 2010; Huang and Elledge 1997; Kauppi et al. 1996; Roshick et al. 2000; Uppsten et al. 2006; Voegtli et al. 2001; Wang et al. 1997). Identical columns of residues and columns with conserved or semi-conserved substitutions were identified for each of the five major clades (i.e., R2c, R2lox, R2_ab, R2_e1, and R2_e2) using ClustalW2.

**Fig. 2:**
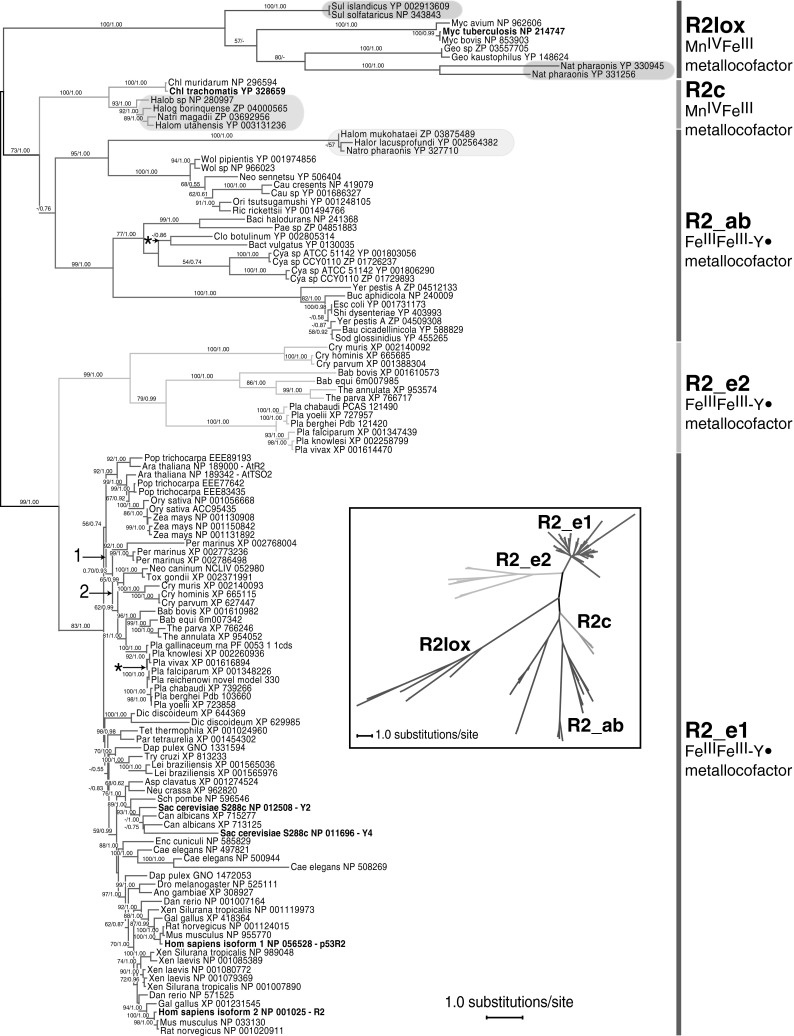
RNR R2 sequences and R2lox homolog proteins group into five major monophyletic clades one of five RAxML analyses (seed #23456). Branches that collapse upon strict consensus of the five RAxML trees are indicated with an asterisk (*). The numbers “1” and “2” represent contrived placement of R2_e2 for the purposes of comparing tree topologies (see Discussion: Origin of the R2_e2 Subunit). Support for each node is represented by bootstrap support and posterior probability values. Archaeal taxa are highlighted in shaded ovals. Taxa in bold include *M. tuberculosis* and *C. trachomatis*, which are characteristic proteins of R2lox and R2c, respectively; the canonical Y2 (*RNR2*) and non-canonical Y4 (*RNR4*) proteins of *S. cerevisiae*; and the canonical R2 and non-canonical p53R2 human proteins. *Inset*: a radial phylogram

Additional structure and sequence-based alignments were generated and evaluated, with inferior results relative to our current knowledge of the structure and function of RNR. Structure-based alignments included the following: (1) STRAP’s implementation of TM-align (Zhang and Skolnick 2005) to create a template, followed by data alignment with ClustalW, (2) MAFFT version 6 (Katoh and Toh 2008) alignment utilizing the STRAP-generated CE template, (3) MAFFT alignment utilizing the STRAP-generated TM-align template, and (4) EXPRESSO (3D-Coffee) as implemented by the T-Coffee server (Armougom et al. 2006). Sequence-similarity-based alignments used MAFFT and combinations of the following options to generate alternative alignments: E-INS-i versus G-INS-i algorithms, JTT100 versus JTT200 scoring matrices, gap opening penalties of 1.53, 2.0, 2.5, and 3.0, and offset values of 0, 0.5, and 1.0. RAxML version 7.2.5 (Stamatakis 2006) analyses of these alternative datasets (results not shown) consistently produced hypotheses of relationships congruent with Fig. 2.

### Phylogenetic Analyses

The AIC, AICc, and BIC criteria provided by ProtTest version 10.2 (Abascal et al. 2005) were used to determine the best-fit model for the data (LG and Γ = 0.66). Phylogenetic analyses included maximum likelihood and Bayesian approaches. Maximum likelihood analyses using the LG+G model were conducted with RAxML on the TeraGrid cluster via the CIPRES portal version 2.2 (Miller et al. 2010). An initial test analysis using the autoMRE criterion (Pattengale et al. 2010) to allow RAxML to halt the number of bootstraps (BS) automatically, showed 350 BS to be adequate. Five RAxML analyses utilizing different starting seeds were executed for 1,000 BS replicates, followed by ML optimization to find the best-scoring tree. Preliminary Bayesian analyses of 1 million generations were conducted using the hybrid MPI/OpenMPI version of MrBayes version 3.1.2 (Ronquist and Huelsenbeck 2003) via the CIPRES portal. The purpose of these test analyses was to optimize mixing of chains by utilizing a variety of mixing temperatures (0.05, 0.1, 0.15, 0.20). Subsequently, two exhaustive analyses, each of which consisted of 4 runs, 6 chains per run, a temperature of 0.05, in which MrBayes was allowed to estimate all parameters, were executed for 3.5 million and 5 million generations on the Texas A&M Brazos cluster. As described in Results: Phylogenetic Analyses, a variety of means were used to assess convergence of the MrBayes MCMC chains and to identify unusual splits (bipartitions). Trees were constructed using Dendroscope version 2.7.4 (Huson et al. 2007). Synapomorphies supporting the R2_e1 and R2_e2 clades were identified using the Trace All Changes function in MacClade version 4.08 (Maddison and Maddison 2000), which used parsimony to reconstruct ancestral states. The R2_e2 characters were identified and highlighted in the alignment document (Supplemental Fig. S1).

## Results

### Evaluation of Competing Alignments

Large evolutionary distances, and the resulting sequence divergence and length heterogeneity, posed a challenge to the alignment of these sequences, and a variety of approaches were used (see Materials and Methods: Data Collection and Alignment). Resulting alignments were compared by evaluating the position of documented functionally and structurally conserved residues, minimization of alignment length, and maximization of the number of columns with identical residues and conserved or semi-conserved substitutions. The most accurate alignment was derived from a structure-based alignment template generated by CE with subsequent ClustalW alignment of the whole dataset, as implemented in STRAP. Similarly, a structure-based alignment approach was used successfully to align α (R1) subunits of the three classes of RNR (Torrents et al. 2002). The final corrected alignment was 920 characters, of which 200 characters were constant, 231 variable characters were parsimony-uninformative, and 489 characters were parsimony-informative (as determined with PAUP* 4.0b10 (Swofford 2003)). All competing alignments were subjected to RAxML analyses and resulted in the same phylogenetic relationship among the five major clades (see Results: Phylogenetic Relationships).

Searches using BLAST to find the most similar experimentally determined protein structures in the RCSB Protein Data Bank resulted in the identification of 19 unique entries (Supplemental Tables S1 and S2) upon which the alignment template created by CE was built. We compared the secondary structures predicted by the Define Secondary Structure of Proteins (DSSP) (Kabsch and Sander 1983) for *S. cerevisiae* Y2 and Y4, *H. sapiens*, *M*. *musculus*, *P. vivax*, *P. yoelii*, *B. halodurans*, *E. coli*, *C. trachomatis*, and *M. tuberculosis* to the STRAP produced alignment. This comparison revealed that positional homology of the residues was not conserved for the three helices α1, α2, and α3, but that helices αA, α4, αB, αC, α5, αD, αE, αF, αG, and αH were directly comparable (Supplemental Fig. S1). Furthermore, detailed study of residue alignment and conservation across functionally relevant residue positions (see below and Supplemental Table S3) showed that the alignment obtained was consistent with the alignments of previous studies in terms of statements of positional homology (Andersson and Högbom 2009; Högbom et al. 2004; Högbom 2010; Huang and Elledge 1997; Kauppi et al. 1996; Roshick et al. 2000; Uppsten et al. 2006; Voegtli et al. 2001; Wang et al. 1997).

### Phylogenetic Analyses

We used two phylogenetic methods to estimate the evolutionary relationships among RNR class I small subunits and R2lox proteins: (1) maximum likelihood, for which five RAxML analyses were run, each with a different starting seed value, and (2) a Bayesian approach implemented in MrBayes. Two MrBayes analyses, each with four independent runs of six chains, ran for 3.5 and 5 million generations, respectively.

The RAxML analyses each resulted in one most likely tree, with nearly identical likelihood scores (range −47,133.307532 to −47,133.610626). For the MrBayes analyses, chain swapping of the six chains for each of the four runs ranged from 17 to 63 % and 23 to 65 % for the 3.5 and 5 million generation analyses, respectively. Convergence was assessed by evaluating (1) average standard deviation of split frequencies (ASDFS), which were well below the recommended value of 0.01; (2) the -Ln cold-chain score of the four runs, which were similar; (3) the potential scale reduction factor (PSRF) for TL, alpha, and branch lengths, all of which were, or approached, 1.000; and (4) the slide, compare, and cumulative commands of AWTY (Are We There Yet?) (Nylander et al. 2008). The ASDFS, cold-chain scores, and PSRF scores were indicative of convergence (Supplemental Table S4). AWTY’s compare command showed a tight relationship to the diagonal for all graphed posterior probabilities of splits across the paired MCMC runs, i.e., the four samples were congruent, which is also indicative of convergence. AWTY’s slide and cumulative functions were less supportive of convergence, in some cases showing trends in the posterior probabilities’ plots in both the 3.5 and 5 million generation analyses. Posterior probability of amino acid models was 1.00 (SD = 0.000) for the Wagner model and 0.00 (SD = 0.000) for all other models in both analyses. No unusual splits across the four MrBayes runs for each of the analyses were identified using AWTY’s “showsplits” command, suggesting that there were no “rogue taxa.”

### Phylogenetic Relationships

One of the five RAxML most likely trees is presented in Fig. 2 (seed #23456). In addition to the maximum likelihood bootstrap support (BS) values, Bayesian posterior probabilities (PP) for the 3.5 million generation analysis are included. See Supplemental Figs. S2–S5 for the four remaining RAxML trees. All trees depicted are unrooted. Strict consensus of the five maximum likelihood trees revealed conflict in only two terminal regions (Fig. 2).

The 3.5 and 5 million generation MrBayes analyses proposed identical hypotheses of relationships. Posterior probability support increased for eight nodes and decreased for seven nodes in comparison with the two analyses, differing by no more than 0.03. The consensus trees are combined and included as Supplemental Fig. S6.

With minor exceptions, the outcomes of the RAxML and MrBayes analyses were congruent with each other in terms of phylogenetic relationships and node support. They differed in (1) the R2lox clade, with a sister relationship of *Mycobacterium* with *Geobacillus* + *Natronomonas* in RAxML that was unresolved (a polytomy) in MrBayes, and (2) in a lack of resolution in the basal R2_e1 clades in the MrBayes analyses. Both the maximum likelihood and Bayesian analyses revealed the presence of five major clades (Fig. 2), defined next in more detail. For the purposes of accurately identifying the five monophyletic clades, we tentatively propose the names R2_ab, R2_e1, and R2_e2 in addition to the conventionally accepted labels R2c and R2lox.

Our goal was to determine the phylogenetic position of the apicomplexan R2 subunits, and thus, our sampling focused on class Ia eukaryotic R2 subunits. While R2c and R2lox protein sampling was restricted with respect to that of standard R2, our phylogenetic analyses allow us to discuss some interesting aspects of their phylogenetic position and relationships.

#### The Standard Class Ia R2 Subunit: Clades R2_ab, R2_e1, and R2_e2

The standard class Ia R2 subunit is the most taxonomically widespread protein, with representation among all three principal domains of life. Unlike class Ib and Ic (R2c) subunits, or R2lox proteins that possess a dimanganese or iron/manganese metal center, class Ia subunits utilize a diiron cofactor to generate a stable tyrosyl radical. However, like R2c, standard R2 subunits typically possess a highly conserved C-terminus tyrosine residue. Interestingly, our analyses show that this subunit’s sequences do not form a monophyletic clade, but in fact represent three clearly distinct clades, R2_ab, R2_e1, and R2_e2 (Fig. 2). Of additional significance was the placement of the apicomplexan-specific R2_e2 clade as sister to the standard eukaryote R2 subunits.

#### The R2_ab Clade (For Archaea and Bacteria)

This monophyletic clade was retained across all maximum likelihood and Bayesian analyses, although support for the clade was <50 % BS and 0.76 PP. Within this clade, there were two distinct and well-supported clades. The first of these clades had 95 % BS/1.00 PP support and consisted of archaeal taxa (*Halorubrum lacusprofundi*, *Halomicrobium mukohataei*, and *Natronomonas pharaonis*) and Proteobacteria (*Caulobacter cresents*, *Caulobacter* sp., *Neorickettsia sennetsu*, *Orientia tsutsugamushi*, *Rickettsia rickettsii*, *Wolbachia pipientis*, and *Wolbachia* sp.). The archaeal sequences were sister to the bacterial clade, and both clades had 100 % BS and 1.00 PP support. The bacteria-only clade consisted of a Rickettsiales + Caulobacterales, with the former polyphyletic. The second clade had 99 % BS/1.00 PP support and consisted solely of bacteria. The R2_ab clade was consistently sister to the R2c clade, with 73 % BS/1.00 PP support for this arrangement.

#### The R2_e1 Clade (For Eukaryotes, Clade 1, Which Includes Orthodox R2)

The eukaryotic standard R2 clade, R2_e1, was supported with 83 % BS/1.00 PP. Many of the phylogenetic relationships proposed for these proteins reflected the accepted species tree, including the monophyly of sequences from several well-established taxonomic groups such as plants, apicomplexans, trypanosomatids, fungi, animals, opisthokonts, and vertebrates. Most of the eukaryotic taxa were represented by at least two differing small subunit sequences, which in most cases are known to be encoded by different loci. However, with the exception of *Perkinsus marinus*, all apicomplexan taxa sampled had only one copy of R2_e1. Interestingly, there are two remarkably different R2_e1 sequences labeled as *Daphnia pulex*, one of which clusters with metazoans and the other with trypanosomatids. The latter might be a contaminant, possibly from a parasite, prey, or symbiont of *Daphnia*.

#### The R2_e2Clade (For Eukaryotes, Clade 2, Which is Apicomplexan Specific)

This novel clade consisted of R2 subunit sequences found only in apicomplexan taxa. It was consistently monophyletic and backed by 99 % BS/1.00 PP support. The R2_e2 clade was sister to the eukaryotic standard R2 proteins (R2_e1) across all analyses, and the joint R2_e1 + R2_e2 clade had support of 99 % BS/1.00 PP. The hypothesis of relationships proposed for the genera sampled (*Babesia*, *Cryptosporidium*, *Plasmodium*, and *Theileria*) was congruent with other studies (Kuo et al. 2008; Kuo and Kissinger 2008). All apicomplexan taxa that possess an R2_e2 protein have only one copy of the orthodox R2_e1 subunit.

#### The Class Ic R2 (R2c) Subunit and Clade

The class Ic R2 small subunit is characterized by the presence of a manganese/iron metal center and the substitution of phenylalanine for the radical-forming tyrosine residue. Like the R2lox proteins, this subunit is limited in distribution to Archaea and bacteria. However, R2c proteins lack the active site cross-link found in R2lox proteins and possess the conserved C-terminus tyrosine found in standard R2 proteins and it is believed that these enzymes accomplish one-electron oxidation.

The R2c subunit was represented by both bacterial and archaeal taxa, namely *Chlamydia trachomatis* and *Chlamydia muridarum* (bacteria) and *Halobacterium* sp., *Halogeometricum borinquense*, *Halomicrobium utahensis*, and *Natrialba magadii* (Archaea). This clade was consistently monophyletic across all analyses with 100 % BS and 1.00 PP support. Our results support a sister group relationship between class Ic (R2c) and the archaeal and bacterial class Ia (R2_ab) subunits included in this analysis, to the exclusion of the apicomplexan-specific and eukaryotic class Ia R2 subunits.

#### The R2lox Proteins and Clade

Similar to class Ic RNR subunits, the R2lox proteins utilize a manganese/iron metal center and lack a tyrosyl radical; however, they also lack the highly conserved C-terminus tyrosine typical of standard R2 enzymes, possess a unique tyrosine–valine cross-link at the active site, and may accomplish two-electron oxidation. As with R2c, the R2lox clade had strong support in both maximum likelihood (100 % BS) and Bayesian (1.00 PP) analyses. The sequences that formed the monophyletic R2lox clade included representatives from *Natronomonas pharaonis*, *Sulfolobus islandicus*, and *Sulfolobus solfataricus* (Archaea) and *Geobacillus kaustophilus*, *Geobacillus* sp., *Mycobacterium avium*, *Mycobacterium bovis*, and *Mycobacterium tuberculosis* (bacteria).

In summary, the maximum likelihood and Bayesian analyses of Class Ia and Ic R2 subunits and R2lox proteins revealed five distinct clades. One of them, namely R2_ab, demonstrated weak-to-moderate (<50 % BS, 0.76 PP) support, and the remaining four (R2_e1, R2_e2, R2c, and R2lox) were consistently and strongly supported. Furthermore, the R2_e2 apicomplexan-specific clade was always found to be sister to the standard eukaryote R2 subunits. Ancestral character state reconstruction inferred by MacClade within a parsimony framework identified 21 unambiguous character states that supported the R2_e2 clade (Supplemental Table S5a) and 30 unambiguous character states that supported the R2_e1 clade (Supplemental Table S5b).

### Clade-specific sequence consistency and conservation

**Fig. 3:**
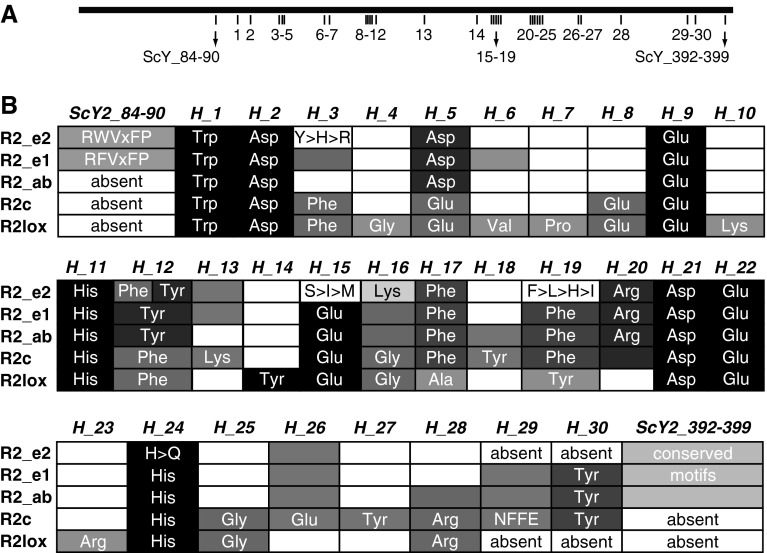
Distribution of characteristic residues across the RNR R2 clades and R2lox homolog proteins **a** The positions as located in the alignment. Individual numbers *1*–*30* represent Högbom positions (*H_1*, *H_2*, etc.), and “*ScY2*” indicates *S. cerevisiae* Y2 coordinates. **b** *Shaded blocks* with *text* represent conserved residue positions. *Shaded blocks* that *lack text* show that the residue was predominantly found in the respective clade, but had not previously been documented. Motifs or residues that were absent are indicated as so, while *blank cells* indicate a variety of residues occurring in that position. In the case of positions *H_3*, *H_15*, *H_19*, and *H_24* where residues were fairly conserved or characteristic for a position for all but the R2_e2 clade, residues are shown in descending order of abundance (single-letter amino acid notation)

Amino acid residues conserved in each of the five major clades identified in Fig. 2 were mapped onto the protein alignment (Supplemental Fig. S1). Sequence conservation could be due to phylogenetic inertia (i.e., shared derived characters that have not yet changed), or to actual structural and/or functional constraints. The results presented here focus on residues conserved across well-studied taxa, many of which have characterized functions (Fig. 3). To facilitate comparison across studies, residues are referenced in terms of *S. cerevisiae* Y2 coordinates (*ScY2_X*) (Voegtli et al. 2001) and as Högbom positions (*H_X*) (Högbom 2010), where “X” represents the alignment coordinate in the respective study. A more detailed accounting of these and additional positions identified in the alignment is presented in Supplemental Table S3, which also provides the matrix position (*M_X*) of each residue in question.

With few exceptions (Fig. 3 and Supplemental Fig. S1), eight residues were identical across all five clades: *H_1*, S*cY2_108* (Trp); *H_2*, *ScY2_118* (Asp); *H_9*, *ScY2_176* (Glu); *H_11*, *ScY2_179* (His); *H_15*, *ScY2_239* (Glu); *H_21*, *ScY2_272* (Asp); *H_22*, *ScY2_27*3 (Glu); *H_24*, *ScY2_276* (His). Five of these positions (*H_9*, *H_11*, *H_15*, *H_22*, and *H_24*) are iron-coordinating residues involved in ligand formation (Högbom et al. 2004). The sixth iron-coordinating residue, *H_5*, *ScY2_145*, was Glu in R2c and R2lox but Asp in R2_ab, R2_e1, and R2_e2. Six residues were found to be unique to R2lox, although rare exceptions were noted by Högbom (Högbom 2010): *H_4*, *ScY2_144* (Gly); *H_7*, *ScY2_154* (Pro); *H_10*, *ScY2_178* (Lys); *H_17*, *ScY2_243* (Ala); *H_19*, *ScY2_247* (Tyr); and *H_23*, *ScY2_275* (Arg). Residues at *H_6*, *ScY2_148* (Val) and *H_14*, *ScY2_235* (Tyr), which form the covalent cross-link unique to R2lox (Andersson and Högbom 2009), were consistent across the R2lox taxa, although Val was also found in most R2_e1 taxa at *H_6*. A single residue, *H_16*, *ScY4_240* (Lys), was unique to R2_e2. R2c, R2_ab, and R2_e1 had no unique residues. Two residues, *H_17*, *ScY2_243* (Phe) and *H_26*, *ScY2_307* (Glu), were consistent across the R2_ab, R2_e1, R2_e2, and R2c proteins but not the R2lox proteins.

The residues at positions, *H_17*, *ScY2_243* (Phe); *H_19*, *ScY2_247* (Phe); and *ScY2_269* (Ile) (latter has no Högbom position), are hydrophobic residues, which form a pocket surrounding the tyrosyl free radical (Akiyoshi et al. 2002; Roshick et al. 2000). With the exception of *H_19* in R2_e2, they were generally well conserved. In R2_e1, R2_ab, and some R2_e2 taxa, the radical-harboring tyrosine residue is found at *H_12*, *ScY2_183* (Högbom et al. 2004). The final seven to eight C-terminus residues were conserved across the R2_e1 and R2_e2 sequences; it is the C-terminus of the R2 subunit that binds to a hydrophobic cleft in the R1 subunit to form the holoenzyme (Uhlin and Eklund 1994; Uppsten et al. 2006). While the alignment of these terminal residues in clade R2_ab fails to clearly show conservation, adjustment of the alignment may reveal a motif.

Two striking differences distinguished the R2_e2 clade from its sister clade of orthodox eukaryotic standard R2 (R2_e1). First, the tyrosine involved in the formation of the stable tyrosyl radical, typical of standard R2, was found only in the R2_e2 sequences from *Plasmodium* taxa (position *H_12* Fig. 3 and Supplemental Fig. S1). The R2_e2 sequences from the three *Cryptosporidium* species and from *Babesia bovis* had a phenylalanine in this position, similar to R2c subunits and R2lox proteins. Both *Theileria* species had an isoleucine substitution, while *Babesia equi* had a valine substitution. Substitution of phenylalanine by leucine, isoleucine, and valine has also been documented in R2c proteins (Högbom 2010). Second, in contrast to the R2_e1 and the R2c taxa, the C-terminus tyrosine residue was not conserved in the R2_e2 taxa (position *H_30* Fig. 3 and Supplemental Fig. S1). In fact, this residue appeared to be entirely lacking. While all *Plasmodium* taxa possessed a tyrosine residue four positions downstream (Supplemental Fig. S1, matrix position 884), our alignment hypothesizes no homology with the *H_30* tyrosine residue found in R2_e1 or R2c.

In summary, the majority of functionally relevant R2_e2 residues are conserved when compared to the standard eukaryotic class Ia R2 subunit clades R2_e1 and R2_ab and to a lesser extent, the R2c clade (e.g., *H_17, H_20,* and *H_26*). R2_e1 and R2_e2 were the only sequences with strongly conserved C-terminus motifs, which are essential in the formation of the RNR holoenzyme. Interestingly, the R2_e2 sequences also share similarities with the R2lox proteins, albeit the shared characteristics tend to an absence of characters (e.g., *H_29* and *H_30)*. In conclusion, be it the presence of *H_16*, (Lys), which is unique to R2_e2 or the phylogenetic analyses that placed R2_e2 sister to the R2_e1 clade, the combined evidence indicated that the R2_e2 sequences are distinct from other R2 proteins in both sequence and evolutionary history. Clearly, the R2_e2 sequences are more closely related to the R2_e1 proteins and are not R2_ab, R2c, or R2lox proteins.

## Discussion

### Phylogenetic Relationships

The robust phylogenetic inferences that can be obtained with maximum likelihood or Bayesian approaches can be extremely time-consuming when including many dozens of sequences, spanning wide evolutionary distances. Given our primary goal of inferring the phylogenetic position of apicomplexan small RNR subunits, our dataset includes an extensive collection of sequences of eukaryotic origin, consisting of ~80 sequences from ~40 species. The dataset also includes ~40 bacterial and archaeal taxa sequences representing both R2 (R2_ab and R2c) subunits and R2lox proteins.

Our phylogenetic analyses revealed five strongly supported major clades (Fig. 2). We define these monophyletic clades as R2c, R2lox, R2_ab, R2_e1, and R2_e2. The relationships among these clades are not fully congruent with the current classification of the R2 subunits, which is based on structural and chemical properties. In particular, the R2 subunits of bacterial and archaeal origin that are grouped with eukaryotic R2s to form class Ia are apparently more closely related to R2c subunits than they are to eukaryotic subunits (Fig. 2). As such, our analyses of the R2 subunits suggest the possible need for a different classification, contingent upon more substantive sampling of bacteria and Archaea followed by rigorous phylogenetic analysis.

In light of the fact that R2c subunits utilize a manganese/iron-carboxylate cofactor while the R2_ab clade, much like R2_e1 proteins, purportedly utilizes a diiron cofactor, the sister group relationship between the class Ia R2_ab and the class Ic R2c clades is somewhat surprising. However, a recent study identified the same relationship (Lundin et al. 2010). In addition, and contrary to our results, in the study of Lundin et al. (2010), the R2c clade was found to be polyphyletic: the *Chlamydia*-R2c taxa were sister to a mixed clade of Bacteria, while the archaeal-R2c taxa were monophyletic and sister to a clade containing the chlamydial-R2c as well as several bacterial sequences from Gammaproteobacteria, Actinobacteria, and Alphaproteobacteria, among others. Regarding the monophyly (or lack thereof) of the R2c clade between our study and that of Lundin et al. (2010), the discordance may reflect our limited sampling of R2c sequences and of the bacterial sequences to which they are most similar. Alternatively, the results of Lundin et al. (2010) may reflect the poor performance of the neighbor-joining method when applied to large datasets of very distantly related proteins. Like Lundin et al. (2010), we found eukaryote relationships within the R2_e1 clade to be largely congruent with accepted hypotheses of relationships.

Particularly intriguing in our analyses was the consistent placement of the apicomplexan-specific R2_e2 clade as sister to a clade with all remaining eukaryotic sequences (R2_e1). The implication of this placement for the origin of R2_e2 proteins is discussed below (see Discussion: Origin of the R2_e2 Subunit). Interestingly, in the analysis of a more comprehensive set of R2 subunits (Lundin et al. 2010), five sequences representing what we term the R2_e2 clade were found to be sister to a clade containing the major eukaryotic clade + Bacteroidetes and a second clade of bacterial origin. However, that analysis utilized the neighbor-joining method, which is prone to long-branch attraction at this level of sequence divergence, potentially resulting in erroneous relationship inferences. The more reliable maximum likelihood analysis of a subset of R2 subunits by Lundin et al. (2010) did not include the R2_e2 sequences, and so the placement of R2_e2 sequences remained unresolved.

The R2-homologous R2lox proteins share considerable sequence identity with the R2 and R2c subunits. Of particular note is the presence of a tryptophan in position H_1 (Fig. 3 and Supplemental Fig. S1), which is shared across all R2 and R2lox proteins and which is involved in radical transfer in R2 proteins (Saleh and Bollinger 2006). R2lox proteins were included in our phylogenetic analyses to investigate a potential relationship between apicomplexan-specific R2 and R2lox proteins, as tentatively suggested by sequence similarity. However, our analyses show no close relationship between the two, or with the R2_e2 clade.

### Support for the Novel R2_e2 Apicomplexan Clade

The unique nature of the monophyletic R2_e2 clade and its sister relationship to R2_e1 (eukaryotic standard R2) were well supported across all our RAxML and Bayesian analyses (Supplemental Figs. S2-S6). This relationship was also present in additional phylogenetic analyses of different sequence alignment methods described in Methods and Materials.

To further test the R2_e1 + R2_e2 sister relationship, we estimated the likelihood of alternative placements of the R2_e2 clade. The first alternative hypothesis placed R2_e2 within the larger R2_e1 eukaryote clade and sister to the apicomplexan R2_e1 clade. The second placed R2_e2 within the R2_e1 apicomplexan clade, sister to all Apicomplexa, save the *Perkinsus marinus* taxa (Fig. 2). Topologies were compared using the Shimodaira–Hasegawa test (Shimodaira and Hasegawa 1999), as implemented in the PHYLIP proml application (Felsenstein 1989). Log-likelihood scores for the hypothesized R2_e1 + R2_e2 sister relationship and the manipulated R2_e2 + apicomplexan R2_e1 and R2_e2 + apicomplexan R2_e1 save *Perkinsus marinus* relationships were −49,740.2, −49,754.4, and −49,755.0, respectively. The proposed R2_e1 + R2_e2 sister relationship provides a significantly better fit to the data than either of the manipulated topologies (*P* value ~0.000 for both).

Furthermore, using a parsimony framework, we identified several dozen amino acid residues that support the separate clades R2_e1 and R2_e2. While this method has its limitations (Cunningham 1999; Losos 1999), the utility of looking at characters in the context of ancestral state reconstruction is well demonstrated (Mathews et al. 2002; Nie et al. 2010) and has been used to infer support (Morton and Msiska 2010).

However, we were unable to identify amino acid substitutions related to functional divergence between R2_e1 and R2_e2. The majority of the residues that differentiated the two clades were variable within each clade, the substitutions were often homoplastic (Supplemental Tables S5a, b), and none of these residue positions was of known structural or functional significance (Supplemental Fig. S1).

### Origin of the R2_e2 Subunit

The origin of the R2_e2 lineage is difficult to assert. The fact that the R2_e2 gene subtree agrees with the postulated species tree for the apicomplexan taxa represented (Zhu et al. 2000; Silva et al. 2011), and that the gene is in a region of conserved synteny in several Apicomplexa genera, provides extremely compelling evidence for its presence early in the evolution of the Apicomplexa phylum. The phylum dates back to at least 600 million years (Douzery et al. 2004), so the R2_e2 lineage is quite old. However, the sister group relationship between R2_e2, present only in the Apicomplexa, and R2_e1, the orthodox class Ia R2 subunit present in most eukaryotes, is puzzling. At least four scenarios can account for the distribution of small subunit RNR proteins in apicomplexans:

1. Taken at face value, the phylogenetic position of the apicomplexan R2_e2 clade suggests an ancient R2 duplication near the origin of the eukaryotes, giving rise to the R2_e1 and R2_e2 paralog lineages, with R2_e2 copies being subsequently lost in all eukaryotic lineages other than the Apicomplexa. Since the Apicomplexa phylum is not sister to the remaining eukaryote clades (Burki et al. 2012; Ciccarelli et al. 2006; Parfrey et al. 2010), this hypothesis would require several independent losses of the R2_e1 paralog in eukaryotes, including, at a minimum, losses from plants, heterokonts, and non-apicomplexan alveolates.
2. R2_e2 could have resulted from a duplication of R2_e1 early in the evolution of the phylum Apicomplexa, followed by rapid sequence divergence. Given the time frame involved (the phylum likely dates back >600 My), it is possible that any phylogenetic signal placing the R2_e2 clade as a sister group to the apicomplexan R2_e1 has been erased by multiple substitutions, a process that could have been facilitated by functional divergence of one of the duplicates.
3. The ancestor to the R2_e2 clade could have resulted from a horizontal transfer event from an Archaea or bacterial taxon into an early apicomplexan, followed by sequence convergence to conform to eukaryotic functional or structural requirements. The placement of R2_e2 as sister to R2_e1 would then result from convergence, rather than shared evolutionary history. However, while sequence convergence is often invoked, molecular convergence in the sense of globally similar sequences (nucleotides or amino acids) having evolved from unrelated ancestors has yet to be convincingly demonstrated (Doolittle 1994; Patterson 1988).
4. Another intriguing possibility is the transfer into the nucleus from the original apicoplast genome, thought to be derived from red algae (Fast et al. 2001; Janouškovec et al. 2010). Such transfer would have to have occurred before the diversification of the phylum, as *Cryptosporidium* species have R2_e2 but lack an apicoplast (supposedly a secondary loss (Barta and Thompson 2006)). Gene transfers between plastid and nuclear genomes are not uncommon in apicomplexans. Many genes for apicoplast proteins are encoded in the host’s nuclear genome (van Dooren et al. 2002). Studies of the *Plasmodium* genome have identified 551 nuclear chromosome gene products that are targeted to the plastid, including housekeeping enzymes involved in DNA replication and repair (Gardner et al. 2002). In *Cryptosporidium*, some 31 genes of plastid/endosymbiont origin were recorded (Huang et al. 2004). Much like for hypothesis (2), under this scenario, the placement of the R2_e2 group would have to result from rapid sequence divergence to erase the phylogenetic signal associated with the standard eukaryotic R2_e1 sequences.

The gene structure of R2_e1 and R2_e2 provides no insights as to the origin of R2_e2. R2_e1 and R2_e2 are single exon genes in *Cryptosporidium*, both have multiple exons in *Theileria* and *Babesia*, and in the genus *Plasmodium*, R2_e1 is a single exon gene, but R2_e2 has 5 exons. Therefore, the structure of the genes seems to reflect the average gene structure of their respective genomes, since *Cryptosporidium* has the smallest average number of introns per gene (< 0.5), while *Babesia* and *Theileria* have the highest (1.7 and ~2.5, respectively).

The chromosomal location of the two genes is perhaps more informative. If the R2_e2 gene originated from a duplication event, one might expect the two paralogs to be located in tandem in the genome. We found this to be the case in one genus, *Cryptosporidium*. On the other hand, in *Babesia* and *Theileria*, they are in the same chromosome but several thousand base pairs apart, while in *Plasmodium,* they are in different chromosomes. The difference between genera is not unexpected, since they have different chromosome numbers, ranging from 14 in *Plasmodium* to four in both *Theileria* and *Babesia*, and synteny across genera is limited. However, if R2_e2 was acquired by horizontal transfer from another species or organelle, the probability that the insertion point would be next to its very divergent homolog seems quite low. Therefore, the tandem arrangement of the two genes in *Cryptosporidium* seems to suggest an ancient duplication as described in hypotheses (1) or (2) above, with chromosomal rearrangement in the other genera throughout the last 600 MY, resulting in the break in linkage between the two loci.

### Function of the Apicomplexan R2 Subunit R2_e2

Many eukaryotes have two or more R2 protein-coding loci, and yet except in humans and a few model organisms, the role of the resulting proteins has been little studied. Humans and mice have two R2 subunits, with humans possessing the canonical hRRM2 and the non-canonical p53R2 and it has been suggested that both subunits are essential (Zhou et al. 2010). Like hRRM2, p53R2 subunits form a holoenzyme with R1 with an iron–tyrosyl free radical (Guittet et al. 2001). In humans, the subunits have evolved different roles with hRRM2 maintaining the dNTP pool for DNA replication during S phase, while the non-canonical p53R2, once thought to be solely involved in DNA repair, is now believed to be involved in mitochondrial DNA replication, or both processes (Bourdon et al. 2007; Håkansson et al. 2006). In contrast, the active holoenzyme of the yeast *Saccharomyces cerevisiae* contains two different small subunits, in the form α_2_ββ′ (Perlstein et al. 2005). While the canonical form Y2 (*RNR2*) produces the free radical, the non-canonical Y4 (*RNR4*) lacks key residues needed to form a diiron center (Sommerhalter et al. 2004) and may instead play a chaperone role (Cotruvo and Stubbe 2011).

The apicomplexan taxa examined also possess a non-canonical R2 subunit. However, while the non-canonical subunits of humans, mice, and yeast fall within the same clade as the canonical subunits, i.e., clade R2_e1, the non-canonical apicomplexan R2 subunits form a distinct clade, sister to the R2_e1 clade. The clade R2_e2 is exclusive to apicomplexan parasites.

The role of R2_e2 in apicomplexans remains to be characterized. The presence of intact open reading frames in all apicomplexan taxa where this subunit is found is congruent with a functional role, but the long-branch lengths in the R2_e2 clade relative to R2_e1 suggest that the function of R2_e2 is more resilient to changes in the primary sequence of the protein. In *Plasmodium falciparum*, the only taxon for which Re_e2 has been studied, the two subunits, PfR2 (R2_e1) and PfR4 (R2_e2), were found to interact with one another and with the R1 subunit to form an α_2_ββ′ complex (PfR1_2_/PfR2/PfR4) (Bracchi-Ricard et al. 2005), similar to the suggested active form in *S. cerevisiae* (Perlstein et al. 2005) and human RNRs (Yanamoto et al. 2005). Our analyses show that R2_e2, much like Y4 in yeast, seems to lack key residues to produce a free radical and is therefore likely to play a complementary role to R2_e1. Experiments are needed to substantiate the functional role of this apicomplexan-specific copy of the RNR R2 subunit.

Even though its function remains elusive, the documented interaction of R2_e2 with the other RNR subunits (R1 and R2_e1) in *P. falciparum* (Bracchi-Ricard et al. 2005), taken together with the conservation of most of the key functional residues in R2_e2 (Munro and Silva, 2012), suggests that this subunit may in fact be an integral component of the RNR holoenzyme and hence a bona fide target of RNR-directed therapeutics. RNR inhibitors work by a variety of ways and may act at the translation level preventing synthesis of the enzyme or at the protein level to prevent the formation of the holoenzyme or inhibit a fully formed enzyme (reviewed in (Munro and Silva 2012)). In terms of evolutionary history and primary sequence, the R2_e2 lineage is clearly distinct from the human R2 subunits, revealing the potential of apicomplexan RNR as a therapeutic target. In particular, the C-terminus residues of the *Plasmodium* R2_e2 are very conserved (QIxFDEDF or QIxLDEDF, where “x” is variable) and quite distinct from the human terminal residues (NxFTLDADF), a difference that may be exploited to prevent the formation of the holoenzyme (Fisher et al. 1995; Ingram and Kinnaird 1999; Rubin et al. 1993).

## Electronic supplementary material

Below is the link to the electronic supplementary material.

Supplementary material 1 (DOC 356 kb)

Supplementary material 2 (PDF 1643 kb)

Supplementary material 3 (PDF 208 kb)

Supplementary material 4 (DOCX 29 kb)
