# Supplementary material for: A Novel Clade of Unique Eukaryotic Ribonucleotide Reductase R2 Subunits is Exclusive to Apicomplexan Parasites
Source: J Mol Evol. 2013 Sep 18;77(3):92–106. doi: 10.1007/s00239-013-9583-y (PMC3824934; doi:10.1007/s00239-013-9583-y)
Supplement: Supplementary file 1 — Supplementary material 1 (DOC 356 kb) [file 239_2013_9583_MOESM1_ESM.doc]

**Notes:**

121 taxa, 920 characters

**Alignment generation:**

Alignment was created by gathering 19 RCSB PDP structures which were aligned using CE in STRAP to create a template alignment based of α-carbon positions. ClustalW 2 was then used to align the 131 taxa to this template alignment. Manual correction follows.

**Manual “corrections”:**

1. shifted LDQ of Hom_sapiens_B_isoform_3_NP_001165949 to match LDQ of Hom_sapiens_isoform_1_NP_056528 (~matrix position 260)

2. shifted D and E of Högbom #5 to align with unorthodox Bab_bovis_XP_001610573, Bab_equi_6m007985, The_annulata_XP_953574, Cry_hominis_XP_665685, Cry_parvum_XP_001388304 - easier than introducing gaps in unorthodox

3. shifted sequences around Högbom #6 & #7 for Geo_kaustophilus_YP_148624, Geo_sp_ZP_03557705 1 position to align w/ others

4. introduced gaps to align Högbom #1 of Nat_pharaonis_YP_330945 and retain alignment of Högbom #2

5. shifted sequence around Högbom #14 to align Nat_pharaonis_YP_331256

6. introduced a gap to align Högbom 19 of Nat_pharaonis_YP_331256 and Nat_pharaonis_YP_330945

7. shifted sequence around Högbom #28 to align Nat_pharaonis_YP_331256 and Nat_pharaonis_YP_330945

8. shifted sequence around Högbom #29 to align Baci_halodurans_NP_241368

9. shifted sequence around Högbom #29 & #30 to align Bau_cicadellinicola_YP_588829, Esc_coli_YP_001731173, Shi_dysenteriae_YP_403993, Sod_glossinidius_YP_455265, Yer_pestis_A_ZP_04509308, Buc_aphidicola_NP_240009, Yer_pestis_A_ZP_04512133, Chl_muridarum_NP_296594, Chl_trachomatis_YP_328659

10. Moved N and P, prior to Högbom #29 so as to align for most taxa

11. gaps introduced across alignment to align Högbom #30 of Pla_yoelii_XP_727957 w/ other unorthodox Plasmodium

12. shifted Per_marinus_XP_002768004 residues to align Högbom positions 9 & 11 & 12

13. Halom_mukohataei_ZP_03875489, Natro_pharaonis_YP_327710, Halor_lacusprofundi_YP_002564382, shifted E into position under Högbom position 5 as it represents a conserved substitution.

**Coordinates:**

Coordinates follow those of Voegtli et al. [1] for *S. cerevisiae* Y2, chain A 1JK0 (NP_012508 is Y2-like).

**Secondary structure:**

Taxa in blue font = the 19 PDB structures used to create the template alignment. The DSSP secondary structures [2] are mapped to corresponding taxa in the alignment.

H = alpha helix

B = residue in isolated beta-bridge

E = extended strand, participates in beta ladder

G = 3-helix (3/10 helix)

I = 5 helix (pi helix)

T = hydrogen bonded turn

S = bend

**Residues of importance:**

[3]

| = Högbom’s 8 sites consistent across all taxa: positions 1, 2, 9, 11, 15, 21, 22, 24.

| = Högbom’s residues consistent across R21ox only: positions 4, 7, 10, 17, 19, 23 were found to be unique. Positions 6, 14, and the position between 19 and 20, while consistent across R2lox, the residues were also found in other R2 small subunits.

| = Högbom’s residues consistent across R2c only: position between 2 and 3, 13, 18, position between 25 and 26, 26, 27, 3 positions between 27 and 28, 2 positions between 28 and 29, 29, while consistent across R2c, the residues were also found in other R2 small subunits.

| = Högbom’s residues consistent across Mn/Fe (R2c and R2lox) proteins only: position between positions 1 and 2, 3, 5, 8, 12 16, 25, 28, while consistent across R2c and R2lox (with the exception of position 28), the residues were also found in other R2 small subunits

| = Högbom’s residues consistent across R2c, R2_ab, and R2_e2 proteins only (i.e. C-terminus containing Tyr): positions 17, 19, 30.

| = Högbom’s residues consistent across R2_ab and R2_e2 only (Note: radical harboring residue in position 12): positions 5, 12, 20

* = Högbom notes rare exceptions

↓ and ↓ = 16 sites, formerly considered to be conserved until the identification of *S. cerevisiae* Rnr4p (i.e. Y4) (↓ = conserved and ↓ no longer conserved (i.e. histidine 179 to tyrosine in Y4, phenylalanine 243 to glutamine in Y4, serine 246 to asparagine in Y4, phenylalanine 247 to tyrosine in Y4, glutamic acid 273 to argenine in Y4, histidine 276 to tyrosine in Y40) [4]

⧫ = electron transfer pathway residues in E. coli [5] and an additional site ⧫ [6]

❖ = electron transfer pathway residues in C. trachomatis [7]

◊ = electron transfer pathway residues in Salmonella typhimurium [7,8]

⚫ = diiron center residues [6,8-10] Högbom positions [3] 5, 9, 11, 15, 22, 24 - i.e. conserved iron ligands (diiron cluster formation) across *S. cerevisiae* Y2 and Y4, mouse R2 and E. coli R2 [1].

⯀ = hydrophobic residues forming pocket surrounding the tyrosyl free radical, Högbom positions [3] 17, 19, and position 269 of *S. cerevisiae* (yellow highlighting) [6]

‡ = loop resides [11] where green highlighting = conserved residues, residues in white font = taxa documented in [11] and pink highlighting = exception to the otherwise highly conserved phenylalanine.

substitutions:

* = identical

: = conserved

. = semi-conserved

Blue highlighting = the unambiguous changes supporting the unorthodox clade as traced using MacClade

**References**

1. Voegtli WC, Ge J, Perlstein DL, Stubbe J, Rosenzweig AC (2001) Structure of the yeast ribonucleotide reductase Y2Y4 heterodimer. Proc Natl Acad Sci USA 98: 10073-10078.

2. Kabsch W, Sander C (1983) Dictionary of protein secondary structure: Pattern recognition of hydrogen-bonded and geometrical features. Biopolymers 22: 2577-2637.

3. Högbom M (2010) The manganese/iron-carboxylate proteins: What is what, where are they, and what can the sequences tell us? J Biol Inorg Chem 15: 339-349.

4. Wang PJ, Chabes A, Casagrande R, Tian XC, Thelander L, et al. (1997) Rnr4p, a novel ribonucleotide reductase small-subunit protein. Molecular and Cellular Biology 17: 6114-6121.

5. Jordan A, Reichard P (1998) Ribonucleotide reductases. Annu Rev Biochem 67: 71-98.

6. Roshick C, Iliffe-Lee ER, McClarty G (2000) Cloning and characterization of ribonucleotide reductase from *Chlamydia trachomatis*. J Biol Chem 275: 38111-38119.

7. Högbom M, Stenmark P, Voevodskaya N, McClarty G, Gräslund A, et al. (2004) The radical site in chlamydial ribonucleotide reductase defines a new R2 subclass. Science 305: 245-248.

8. Uppsten M, Färnegårdh M, Domkin V, Uhlin U (2006) The first holocomplex structure of ribonucleotide reductase gives new insight into its mechanism of action. Journal of Molecular Biology 359: 365-377.

9. Chakrabarti D, Schuster SM, Chakrabarti R (1993) Cloning and characterization of subunit genes of ribonucleotide reductase, a cell-cycle-regulated enzyme, from *Plasmodium falciparum*. Proc Natl Acad Sci USA 90: 12020-12024.

10. Nordlund P, Sjöberg BM, Eklund H (1990) Three-dimensional structure of the free radical protein of ribonucleotide reductase. Nature 345: 593-598.

11. Sommerhalter M, Voegtli WC, Perlstein DL, Ge J, Stubbe J, et al. (2004) Structures of the yeast ribonucleotide reductase Rnr2 and Rnr4 homodimers. Biochemistry 43: 7736-7742.

matrix position 10 20 30 40 50 60 70 80 90 100 110 120 130 140 150 160 170 180 190 200 210

. . . . . . . . . . . . . . . . . . . . .

Bab_bovis_XP_001610573 ---------------------------------------------------------------------------------------------------------------------------------------------------------------------------------------------------------------------------

Bab_equi_6m007985 ---------------------------------------------------------------------------------------------------------------------------------------------------------------------------------------------------------------------------

The_annulata_XP_953574 ---------------------------------------------------------------------------------------------------------------------------------------------------------------------------------------------------------------------------

The_parva_XP_766717 ---------------------------------------------------------------------------------------------------------------------------------------------------------------------------------------------------------------------------

Pla_berghei_Pdb_121420 ---------------------------------------------------------------------------------------------------------------------------------------------------------------------------------------------------------------------------

Pla_yoelii_XP_727957 ---------------------------------------------------------------------------------------------------------------------------------------------------------------------------------------------------------------------------

Pla_chabaudi_PCAS_121490 ---------------------------------------------------------------------------------------------------------------------------------------------------------------------------------------------------------------------------

Pla_falciparum_XP_001347439 ---------------------------------------------------------------------------------------------------------------------------------------------------------------------------------------------------------------------------

Pla_knowlesi_XP_002258799 ---------------------------------------------------------------------------------------------------------------------------------------------------------------------------------------------------------------------------

Pla_vivax_XP_001614470 ---------------------------------------------------------------------------------------------------------------------------------------------------------------------------------------------------------------------------

Cry_hominis_XP_665685 ---------------------------------------------------------------------------------------------------------------------------------------------------------------------------------------------------------------------------

Cry_parvum_XP_001388304 ---------------------------------------------------------------------------------------------------------------------------------------------------------------------------------------------------------------------------

Cry_muris_XP_002140092 ---------------------------------------------------------------------------------------------------------------------------------------------------------------------------------------------------------------------------

Ano_gambiae_XP_308927 ---------------------------------------------------------------------------------------------------------------------------------------------------------------------------------------------------------------------------

Dro_melanogaster_NP_525111 ---------------------------------------------------------------------------------------------------------------------------------------------------------------------------------------------------------------------------

Dan_rerio_NP_571525 ---------------------------------------------------------------------------------------------------------------------------------------------------------------------------------------------------------------------------

Gal_gallus_XP_001231545 ---------------------------------------------------------------------------------------------------------------------------------------------------------------------------------------------------------------------------

Hom_sapiens_isoform_2_NP_001025 ---------------------------------------------------------------------------------------------------------------------------------------------------------------------------------------------------------------------------

Mus_musculus_NP_033130 ---------------------------------------------------------------------------------------------------------------------------------------------------------------------------------------------------------------------------

Rat_norvegicus_NP_001020911 ---------------------------------------------------------------------------------------------------------------------------------------------------------------------------------------------------------------------------

Xen_laevis_NP_001079369 ---------------------------------------------------------------------------------------------------------------------------------------------------------------------------------------------------------------------------

Xen_Silurana_tropicalis_NP_001007890 ---------------------------------------------------------------------------------------------------------------------------------------------------------------------------------------------------------------------------

Xen_laevis_NP_001080772 ---------------------------------------------------------------------------------------------------------------------------------------------------------------------------------------------------------------------------

Xen_laevis_NP_001085389 ---------------------------------------------------------------------------------------------------------------------------------------------------------------------------------------------------------------------------

Xen_Silurana_tropicalis_NP_989048 ---------------------------------------------------------------------------------------------------------------------------------------------------------------------------------------------------------------------------

Gal_gallus_XP_418364 ---------------------------------------------------------------------------------------------------------------------------------------------------------------------------------------------------------------------------

Hom_sapiens_isoform_1_NP_056528 ---------------------------------------------------------------------------------------------------------------------------------------------------------------------------------------------------------------------------

Mus_musculus_NP_955770 ---------------------------------------------------------------------------------------------------------------------------------------------------------------------------------------------------------------------------

Rat_norvegicus_NP_001124015 ---------------------------------------------------------------------------------------------------------------------------------------------------------------------------------------------------------------------------

Xen_Silurana_tropicalis_NP_001119973 ---------------------------------------------------------------------------------------------------------------------------------------------------------------------------------------------------------------------------

Dan_rerio_NP_001007164 ---------------------------------------------------------------------------------------------------------------------------------------------------------------------------------------------------------------------------

Dap_pulex_GNO_1472053 ---------------------------------------------------------------------------------------------------------------------------------------------------------------------------------------------------------------------------

Asp_clavatus_XP_001274524 ---------------------------------------------------------------------------------------------------------------------------------------------------------------------------------------------------------------------------

Neu_crassa_XP_962820 ---------------------------------------------------------------------------------------------------------------------------------------------------------------------------------------------------------------------------

Dap_pulex_GNO_1331594 ---------------------------------------------------------------------------------------------------------------------------------------------------------------------------------------------------------------------------

Can_albicans_XP_715277 ---------------------------------------------------------------------------------------------------------------------------------------------------------------------------------------------------------------------------

Can_albicans_XP_713125 ---------------------------------------------------------------------------------------------------------------------------------------------------------------------------------------------------------------------------

Sac_cerevisiae_S288c_NP_012508 ---------------------------------------------------------------------------------------------------------------------------------------------------------------------------------------------------------------------------

Lei_braziliensis_XP_001565976 ---------------------------------------------------------------------------------------------------------------------------------------------------------------------------------------------------------------------------

Lei_braziliensis_XP_001565036 ---------------------------------------------------------------------------------------------------------------------------------------------------------------------------------------------------------------------------

Try_cruzi_XP_813233 ---------------------------------------------------------------------------------------------------------------------------------------------------------------------------------------------------------------------------

Par_tetraurelia_XP_001454302 ---------------------------------------------------------------------------------------------------------------------------------------------------------------------------------------------------------------------------

Tet_thermophila_XP_001024960 ---------------------------------------------------------------------------------------------------------------------------------------------------------------------------------------------------------------------------

Ara_thaliana_NP_189000 ---------------------------------------------------------------------------------------------------------------------------------------------------------------------------------------------------------------------------

Pop_trichocarpa_EEE89193 ---------------------------------------------------------------------------------------------------------------------------------------------------------------------------------------------------------------------------

Ara_thaliana_NP_189342 ---------------------------------------------------------------------------------------------------------------------------------------------------------------------------------------------------------------------------

Pop_trichocarpa_EEE77642 ---------------------------------------------------------------------------------------------------------------------------------------------------------------------------------------------------------------------------

Pop_trichocarpa_EEE83435 ---------------------------------------------------------------------------------------------------------------------------------------------------------------------------------------------------------------------------

Ory_sativa_ACC95435 ---------------------------------------------------------------------------------------------------------------------------------------------------------------------------------------------------------------------------

Zea_mays_NP_001130908 ---------------------------------------------------------------------------------------------------------------------------------------------------------------------------------------------------------------------------

Zea_mays_NP_001131892 ---------------------------------------------------------------------------------------------------------------------------------------------------------------------------------------------------------------------------

Zea_mays_NP_001150842 ---------------------------------------------------------------------------------------------------------------------------------------------------------------------------------------------------------------------------

Ory_sativa_NP_001056668 ---------------------------------------------------------------------------------------------------------------------------------------------------------------------------------------------------------------------------

Per_marinus_XP_002786498 ---------------------------------------------------------------------------------------------------------------------------------------------------------------------------------------------------------------------------

Per_marinus_XP_002773236 ---------------------------------------------------------------------------------------------------------------------------------------------------------------------------------------------------------------------------

Bab_bovis_XP_001610982 ---------------------------------------------------------------------------------------------------------------------------------------------------------------------------------------------------------------------------

Bab_equi_6m007342 ---------------------------------------------------------------------------------------------------------------------------------------------------------------------------------------------------------------------------

The_annulata_XP_954052 ---------------------------------------------------------------------------------------------------------------------------------------------------------------------------------------------------------------------------

The_parva_XP_766246 ---------------------------------------------------------------------------------------------------------------------------------------------------------------------------------------------------------------------------

Cry_hominis_XP_665115 ---------------------------------------------------------------------------------------------------------------------------------------------------------------------------------------------------------------------------

Cry_parvum_XP_627447 ---------------------------------------------------------------------------------------------------------------------------------------------------------------------------------------------------------------------------

Cry_muris_XP_002140093 ---------------------------------------------------------------------------------------------------------------------------------------------------------------------------------------------------------------------------

Neo_caninum_NCLIV_052980 ---------------------------------------------------------------------------------------------------------------------------------------------------------------------------------------------------------------------------

Tox_gondii_XP_002371991 ---------------------------------------------------------------------------------------------------------------------------------------------------------------------------------------------------------------------------

Dic_discoideum_XP_644369 ---------------------------------------------------------------------------------------------------------------------------------------------------------------------------------------------------------------------------

Cae_elegans_NP_497821 ---------------------------------------------------------------------------------------------------------------------------------------------------------------------------------------------------------------------------

Enc_cuniculi_NP_585829 ---------------------------------------------------------------------------------------------------------------------------------------------------------------------------------------------------------------------------

Pla_berghei_Pdb_103660 ---------------------------------------------------------------------------------------------------------------------------------------------------------------------------------------------------------------------------

Pla_chabaudi_XP_739266 ---------------------------------------------------------------------------------------------------------------------------------------------------------------------------------------------------------------------------

Pla_yoelii_XP_723858 ---------------------------------------------------------------------------------------------------------------------------------------------------------------------------------------------------------------------------

Pla_falciparum_XP_001348226 ---------------------------------------------------------------------------------------------------------------------------------------------------------------------------------------------------------------------------

Pla_reichenowi_novel_model_330 ---------------------------------------------------------------------------------------------------------------------------------------------------------------------------------------------------------------------------

Pla_gallinaceum_rna_PF_0053_1_1cds ---------------------------------------------------------------------------------------------------------------------------------------------------------------------------------------------------------------------------

Pla_knowlesi_XP_002260936 ---------------------------------------------------------------------------------------------------------------------------------------------------------------------------------------------------------------------------

Pla_vivax_XP_001616894 ---------------------------------------------------------------------------------------------------------------------------------------------------------------------------------------------------------------------------

Sch_pombe_NP_596546 ---------------------------------------------------------------------------------------------------------------------------------------------------------------------------------------------------------------------------

Sac_cerevisiae_S288c_NP_011696 ---------------------------------------------------------------------------------------------------------------------------------------------------------------------------------------------------------------------------

Per_marinus_XP_002768004 ---------------------------------------------------------------------------------------------------------------------------------------------------------------------------------------------------------------------------

Cae_elegans_NP_500944 MYGKEIQNSTLTAAGFYHDPVIGDHRSPGFQQRCASIYNQDEIQVAANISIDLMNQPRVLLNGCNVKLTVYPNDSKFLIEVYNRDNNTEFQFKITDVYALVNEFDLADGLSNALESSIIEHKIIQYPLISSQVRSFYIESGRLDAPANTLFTSKMPRRIFIGLVDSDAYNGSYDKSPFNFKPHVVFSNSHNFPLCAPHLQTACHFRFLSRSGVVRAMNP

Cae_elegans_NP_508269 -------------------------------------------------------------------------MISRMDPKSHDVIVDELDFSTMPGTQSGVLNSRWTPIGLKNNFQAAGPYEFILTNNSRSYLNLKRTYLIFTFKITNSDGNIVKMETDEAKNTQVYAPINNIAHSIVKNFTLHINSQLAFHNSSNYAYKSYFEHVLMYGKEIQNSTL

Dic_discoideum_XP_629985 ---------------------------------------------------------------------------------------------------------------------------------------------------------------------------------------------------------------------------

Baci_halodurans_NP_241368 ---------------------------------------------------------------------------------------------------------------------------------------------------------------------------------------------------------------------------

Pae_sp_ZP_04851883 ---------------------------------------------------------------------------------------------------------------------------------------------------------------------------------------------------------------------------

Bact_vulgatus_YP_0130035 ---------------------------------------------------------------------------------------------------------------------------------------------------------------------------------------------------------------------------

Clo_botulinum_YP_002805314 ---------------------------------------------------------------------------------------------------------------------------------------------------------------------------------------------------------------------------

Cya_sp_ATCC_51142_YP_001803056 ---------------------------------------------------------------------------------------------------------------------------------------------------------------------------------------------------------------------------

Cya_sp_CCY0110_ZP_01726237 ---------------------------------------------------------------------------------------------------------------------------------------------------------------------------------------------------------------------------

Cya_sp_ATCC_51142_YP_001806290 ---------------------------------------------------------------------------------------------------------------------------------------------------------------------------------------------------------------------------

Cya_sp_CCY0110_ZP_01729893 ---------------------------------------------------------------------------------------------------------------------------------------------------------------------------------------------------------------------------

Cau_cresents_NP_419079 ---------------------------------------------------------------------------------------------------------------------------------------------------------------------------------------------------------------------------

Cau_sp_YP_001686327 ---------------------------------------------------------------------------------------------------------------------------------------------------------------------------------------------------------------------------

Neo_sennetsu_YP_506404 ---------------------------------------------------------------------------------------------------------------------------------------------------------------------------------------------------------------------------

Ori_tsutsugamushi_YP_001248105 ---------------------------------------------------------------------------------------------------------------------------------------------------------------------------------------------------------------------------

Ric_rickettsii_YP_001494766 ---------------------------------------------------------------------------------------------------------------------------------------------------------------------------------------------------------------------------

Wol_pipientis_YP_001974856 ---------------------------------------------------------------------------------------------------------------------------------------------------------------------------------------------------------------------------

Wol_sp_NP_966023 ---------------------------------------------------------------------------------------------------------------------------------------------------------------------------------------------------------------------------

Bau_cicadellinicola_YP_588829 ---------------------------------------------------------------------------------------------------------------------------------------------------------------------------------------------------------------------------

Esc_coli_YP_001731173 ---------------------------------------------------------------------------------------------------------------------------------------------------------------------------------------------------------------------------

Shi_dysenteriae_YP_403993 ---------------------------------------------------------------------------------------------------------------------------------------------------------------------------------------------------------------------------

Sod_glossinidius_YP_455265 ---------------------------------------------------------------------------------------------------------------------------------------------------------------------------------------------------------------------------

Yer_pestis_A_ZP_04509308 ---------------------------------------------------------------------------------------------------------------------------------------------------------------------------------------------------------------------------

Buc_aphidicola_NP_240009 ---------------------------------------------------------------------------------------------------------------------------------------------------------------------------------------------------------------------------

Yer_pestis_A_ZP_04512133 ---------------------------------------------------------------------------------------------------------------------------------------------------------------------------------------------------------------------------

Halom_mukohataei_ZP_03875489 ---------------------------------------------------------------------------------------------------------------------------------------------------------------------------------------------------------------------------

Natro_pharaonis_YP_327710 ---------------------------------------------------------------------------------------------------------------------------------------------------------------------------------------------------------------------------

Halor_lacusprofundi_YP_002564382 ---------------------------------------------------------------------------------------------------------------------------------------------------------------------------------------------------------------------------

Chl_muridarum_NP_296594 ---------------------------------------------------------------------------------------------------------------------------------------------------------------------------------------------------------------------------

Chl_trachomatis_YP_328659 ---------------------------------------------------------------------------------------------------------------------------------------------------------------------------------------------------------------------------

Halob_sp_NP_280997 ---------------------------------------------------------------------------------------------------------------------------------------------------------------------------------------------------------------------------

Halog_borinquense_ZP_04000565 ---------------------------------------------------------------------------------------------------------------------------------------------------------------------------------------------------------------------------

Halom_utahensis_YP_003131236 ---------------------------------------------------------------------------------------------------------------------------------------------------------------------------------------------------------------------------

Natri_magadii_ZP_03692956 ---------------------------------------------------------------------------------------------------------------------------------------------------------------------------------------------------------------------------

Geo_kaustophilus_YP_148624 ---------------------------------------------------------------------------------------------------------------------------------------------------------------------------------------------------------------------------

Geo_sp_ZP_03557705 ---------------------------------------------------------------------------------------------------------------------------------------------------------------------------------------------------------------------------

Myc_avium_NP_962606 ---------------------------------------------------------------------------------------------------------------------------------------------------------------------------------------------------------------------------

Myc_bovis_NP_853903 ---------------------------------------------------------------------------------------------------------------------------------------------------------------------------------------------------------------------------

Myc_tuberculosis_NP_214747 ---------------------------------------------------------------------------------------------------------------------------------------------------------------------------------------------------------------------------

Nat_pharaonis_YP_331256 ---------------------------------------------------------------------------------------------------------------------------------------------------------------------------------------------------------------------------

Nat_pharaonis_YP_330945 ---------------------------------------------------------------------------------------------------------------------------------------------------------------------------------------------------------------------------

Sul_islandicus_YP_002913609 ---------------------------------------------------------------------------------------------------------------------------------------------------------------------------------------------------------------------------

Sul_solfataricus_NP_343843 ---------------------------------------------------------------------------------------------------------------------------------------------------------------------------------------------------------------------------

1JK0:A S. cerevisiae Y2 α1 α2 α3

.......... .................------------------------....... ............. ...

HHHHHHHHHHTHHHHHHHHHHHHTHHHH------------------------HHHHHHH HHHHHHHHHHGGG GGGS T T S S

**1**SMQ:A S. cerevisiae HHHHHHHHHHTTHHHHHHHHHHHHHHHH------------------------HHHHHHTTTTHHHHHHHHGGG GGGS T------T S S

1JK0:B S. cerevisiae Y4 HHHHHHHHHTTT TTTS T T S S

1SMS:A S. cerevisiae HHHHHHTTTTTTHHHHHHHTT GGG S SS SS

3HF1:A H. sapiens TT TTT GGG------S SS S

2UW2:A H. sapiens TT TTTS S

2VUX:A H. sapiens STTT S

1H0N:A M. musculus GGGG TTTSS S SSS

2O1Z:A P. vivax HHHHHHHTT----------------------------------------------------------------------GGG GGGS S SS S

2P1I:A P. yoelii

2RCC:A B. halodurans S TTS SS S SBS S TTS

2ALX:A E. coli SS S GGGS SBSSS S S

1AV8:A E. coli SS S TTSS SBSSS S S

1JPR:A E. coli SS S GGGS SBSSS S S

1JQC:A E. coli SS S GGGS SBSSS S S

1MXR:A E. coli SS S TTSS SBSSS S S

1SYY:A C. trachomatis SSGGGGGGS GGG SSS SS TT S

2ANI:A C. trachomatis SSGGGGGGS GGG SSS SS TT S

3EE4:A M. tuberculosis GGGSTT TTS------------------------------------------------------------------------------------------------

S. cerevisiae S288c NP 012508 (Y2) 10 20 30 40 50 60 70 80 90

coordinates . . . . . . . . .

matrix position 220 230 240 250 260 270 280 290 300 310 320 330 340 350 360 370

. . . . . . . . . . . . . . . .

‡‡‡‡‡‡

Bab_bovis_XP_001610573 ---------------------------MDVGSIKYLQPQEIALEQHNETLLKENAN----------------------------------------------------------------------------------------RWVMFP--

Bab_equi_6m007985 ---------------------------MALRTVKHLSSKEIEGLQANEIVLKANPN----------------------------------------------------------------------------------------RWVMFP--

The_annulata_XP_953574 ------------------------------MIYKYLPYTSILKVQNDEILLKENHN----------------------------------------------------------------------------------------RWVMFP--

The_parva_XP_766717 --------------------------MSGTMVYKYLPCASIEEVQNDEILLKENHN----------------------------------------------------------------------------------------RWVMFP--

Pla_berghei_Pdb_121420 -----------------------------MDKEQYHTQEVLLKAQYNDEILKENQ----------------------------------------------------------------------------------------FRWVMFP--

Pla_yoelii_XP_727957 -----------------------------MDKEQYHTQEVLLKAQYNDEILKENQ----------------------------------------------------------------------------------------FRWVMFP--

Pla_chabaudi_PCAS_121490 -----------------------------MNKEHYHTQEVLLEAQYNDEILKENQ----------------------------------------------------------------------------------------FRWVMFP--

Pla_falciparum_XP_001347439 -----------------------------MSKEQYHDQEVLLEAQNNDEILKENK----------------------------------------------------------------------------------------FRWVMFP--

Pla_knowlesi_XP_002258799 -----------------------------MDKEHYHDQEVLLDAQNNDDILKENQ----------------------------------------------------------------------------------------FRWVMFP--

Pla_vivax_XP_001614470 -----------------------------MDKEHYHDQEVLLDAQNNDDILKENQ----------------------------------------------------------------------------------------FRWVMFP--

Cry_hominis_XP_665685 ---------------------------------MNEAKKELLEQQKNESILLDNP----------------------------------------------------------------------------------------FRWVLFP--

Cry_parvum_XP_001388304 ---------------------------------MNESKKELLEQQKNESILLDNP----------------------------------------------------------------------------------------FRWVLFP--

Cry_muris_XP_002140092 ---------------------------------MIDNRRELLEKQKQETILLDNP----------------------------------------------------------------------------------------FRWVLFP--

: * :: :* * ***:**

Ano_gambiae_XP_308927 -------------------MVLEKENFAENMETIIKNTRKVLTESGANATPTKTPVPMEEEDKKVAAGDADAAVDPKELAKSELSEVHKHESAP------------------------------FDPSIEPLLRDNP------RRFVIFP--

Dro_melanogaster_NP_525111 ------------------MASKENIADNMEKFSLKSPSKKILTDSTNNVRKMSIGHEANGQLAKESSTVNGIGKSANSLMEKSVTP--------------------------------------FDPSLEPLLRENP------RRFVIFP--

Dan_rerio_NP_571525 -----------------MSSTRSPLKTKNENTISTKMNNMSFVDKENTPPSLSSTRILASKTARKIFDESEGQSKAKKG----------------------------------------------AVEEEPLLKENP------HRFVIFP--

Gal_gallus_XP_001231545 ------------------------MLSTRVPLAARQEQPRLSPLKNLALSDKENTPPALSSSRVLASKTARKIFQESEGTPVARG-----------------------------------------AEEEPLLRENP------RRFVIFP--

Hom_sapiens_isoform_2_NP_001025 ------------------------------MLSLRVPLAPITDPQQLQLSPLKGLSLVDKENTPPALSGTRVLASKTARRIFQEPTEPKTKAAAPG------------------------------VEDEPLLRENP------RRFVIFP--

Mus_musculus_NP_033130 ------------------------------MLSVRTPLATIADQQQLQLSPLKRLTLADKENTPPTLSSTRVLASKAARRIFQDSAELESKAPTN-----------------------------PSVEDEPLLRENP------RRFVVFP--

Rat_norvegicus_NP_001020911 ------------------------------MLSVRAPLATIADQQQLHLSPLKRLSLADKENTPPTLSSARVLASKAARRIFQDSAELESKAP-----------------------------TKPSIEEEPLLRENP------RRFVVFP--

Xen_laevis_NP_001079369 -------------------MLSARKPFAELNENVSPMKNLTLTEKENTPSTLNSSRVLASKTARKIFHETETPKSKAPKNP--------------------------------------------RLEDEPLLKDNP------HRFVIFP--

Xen_Silurana_tropicalis_NP_001007890 -------------------MLSARKPFAQLNENVSPMKNLTLAEKENTPPSLNSTRVLASKTARNIFQEAETTKSKAPKDP--------------------------------------------RIQDEPLLKDNP------HRFVIFP--

Xen_laevis_NP_001080772 -------------------MLSARKPFAQLNDNVSPMKNLTLTEKENTPPTLNSTRVLASKTARKIFQEADTPTSKVPKNP--------------------------------------------RFTDEPLLKDNP------HRFVIFP--

Xen_laevis_NP_001085389 -------------------MLSPRNALSPLKENVSPMKRMVLSDKENTVGSAELLVRQTWRCTEQTLTCSSFCRFQPPNVNSDRISRG------------------------TQKELVCQSVKDPLIQDEPLLRDNP------GRFVILP--

Xen_Silurana_tropicalis_NP_989048 -------------------MLSPRNALSPLKDNVSPMKRMALLDKENTPPGFNSGRTSRGTHKQWVCQSLKDP----------------------------------------------------RIQDEPLLRDNP------GRFVILP--

Gal_gallus_XP_418364 -----------------------------MGERRGRAALQEAAGPERSSSPSAAENG-------------------------------------------------------------------LKPHEEPLLRKNP------RRFVIFP--

Hom_sapiens_isoform_1_NP_056528 ------------------------------MGDPERPEAAGLDQDERSSSDTNESEIK--------------------------------------------------------------------SNEEPLLRKSS------RRFVIFP--

Mus_musculus_NP_955770 ------------------------------MGDPERPEAARPEKGEQLCSETEENVVR--------------------------------------------------------------------SNEEPLLRKSS------RRFVIFP--

Rat_norvegicus_NP_001124015 ------------------------------MGDPERPEAARPEEGEQLCPETKENEVR--------------------------------------------------------------------SNEEPLLRKSS------RRFVIFP--

Xen_Silurana_tropicalis_NP_001119973 -----------------------------MGDPGLPTDSGRTGDKNLTNGHSDEE--------------------------------------------------------------------------EPFLRKNP------QRFVIFP--

Dan_rerio_NP_001007164 ------------------------------MNSCTSNTPTVITGYQNGHKDVDPNS----------------------------------------------------------------------VEDEPLLRENP------KRFVIFP--

Dap_pulex_GNO_1472053 -------------------MSFLSQRFDNLRTSDNKENRTNSSGQKILKPHDENVSPTKLTTVEKSVQVRD------------------------------------------------------NDQNEPLLRSNP------SRFVLFP--

Asp_clavatus_XP_001274524 -----------------------------MTAQVTPSKQAASSLENLKMSDSPVKKINFGVAGKENAPSTTPVTDAPVKKIVEKPTEAS----------------------------TKIAAIKELEANEPLLQENP------HRFVLFP--

Neu_crassa_XP_962820 -------------MSVQTSPSKQVTSGIQNLNMDSPAKKLDFGATDKENKPFDEDLAKLEAEIDAEHNANKKAAEAKKMAPTLK----------------------------------------PEEANEPLLTENP------QRFVLFP--

Dap_pulex_GNO_1331594 ----------------------------------MSQLEPILQENKN-------------------------------------------------------------------------------------------------RFVIFP--

Can_albicans_XP_715277 -------MSVQETPTKGLTGKISDLDMSNMKGKSLTDKLAADAKLKDEAQTIHEVKQAVASTDKATEEKDDSLKKHQDFLAKHKVHR---------------------------------HKLKQLEAEEPLLVENK------RRYVMFP--

Can_albicans_XP_713125 -------------------MSKNTDNNKPSPKKVTEKSTPIVTKPESDKTVEKASDDSEIDLPEVYKRHREFLAKHVVNRHKLK----------------------------------------QEESNEPLLTPDK------TRHTIYP--

Sac_cerevisiae_S288c_NP_012508 ------------------------------MPKETPSKAAADALSDLEIKDSKSNLNKELETLREENRVKSDMLKEKLSKDAEN------------------------HKAYLKSHQVHRHKLKEMEKEEPLLNEDK------ERTVLFP--

Lei_braziliensis_XP_001565976 -------------------------MSSTEANGTAAAKRPREEDAEVEVACTSMDGAAADVAKTEDLVIKPIATGAEVLLADKVAEG-------------------------------------TNAEEEPLQQENP------FRYVLFP--

Lei_braziliensis_XP_001565036 ----------------------MAGESKAIAEGAAPAKRVRTEEVETGIERPSGEAARVSSPSTAEDLVIKPIATDAEVLLADKVAEG------------------------------------TNAEEEPLQQENP------FRYVLFP--

Try_cruzi_XP_813233 ------------------------------MSGEKHPREFQLQEQEEPLLRENPD-----------------------------------------------------------------------------------------RYVIFP--

Par_tetraurelia_XP_001454302 ----------------------MIIQNQEMSGNTKTQTFTPLSNVQASQTVLNQN-----------------------------------------------------------------------YCDEPLLKQNP------NRFVLFP--

Tet_thermophila_XP_001024960 -------------------MQQIEQTNEQLFAPSNVDLQKAANPKVNKYLATGMNPLPLLKSKNDEGKAPKEG-----------------------------------------------------YEDEPLLKDNP------NRFVLFP--

Ara_thaliana_NP_189000 -----------------------------MGSLKEGQGRDMEEGESEEPLLMAQNQ----------------------------------------------------------------------------------------RFTMFP--

Pop_trichocarpa_EEE89193 -----------------------------MGSLRNGTESERIREEDKQEPILKEQN---------------------------------------------------------------------------------------QRFCMFP--

Ara_thaliana_NP_189342 --------------------------------MPSMPEEPLLTPTPDR------------------------------------------------------------------------------------------------RFCMFP--

Pop_trichocarpa_EEE77642 --------------------------------MPAIPEEPLLAENPD-------------------------------------------------------------------------------------------------RFCMFP--

Pop_trichocarpa_EEE83435 --------------------------------MPAIPEEPLLAENPD-------------------------------------------------------------------------------------------------RFCMFP--

Ory_sativa_ACC95435 -----------------------------MPAAPTLVPACDLEEPLLAESSE--------------------------------------------------------------------------------------------RFSMFP--

Zea_mays_NP_001130908 -----------------------------MPSAPALVPACDMQEPLLAESSD--------------------------------------------------------------------------------------------RFSMFP--

Zea_mays_NP_001131892 -----------------------------MPSTPTLVPPCDAVEPLLAESSD--------------------------------------------------------------------------------------------RFSMFP--

Zea_mays_NP_001150842 -----------------------------MPAAPTLLPPCDAEEPLLAESSD--------------------------------------------------------------------------------------------RFSMFP--

Ory_sativa_NP_001056668 ----------------------------MPAAAAAKTLVPARGGGDMEEPLLAESS---------------------------------------------------------------------------------------DRFSMFP--

Per_marinus_XP_002786498 -----------------------------MATFSEQMKALEPMEDLLKENPH--------------------------------------------------------------------------------------------RYVMFP--

Per_marinus_XP_002773236 -----------------------------MTSLSEQMKALEPKEDLLKENPH--------------------------------------------------------------------------------------------RYVMFP--

Bab_bovis_XP_001610982 -----------------------------MTDSLSTDDLSSRMKALQSEEFILVEDP--------------------------------------------------------------------------------------NRNGLYP--

Bab_equi_6m007342 -----------------------------MTPSTLTLEMKANEADEFLLNPDP------------------------------------------------------------------------------------------LRIGLYP--

The_annulata_XP_954052 -----------------------------MTMSTLSEKMKALEAEEFILNRNPN------------------------------------------------------------------------------------------RTCLFP--

The_parva_XP_766246 --------------------------------------MKALEAEEFLLNRNPN------------------------------------------------------------------------------------------RTSLFP--

Cry_hominis_XP_665115 --------------------------------------------------------------------------------------------------------------------------------------------------------

Cry_parvum_XP_627447 -----------------------------MTEENNNVILTKETKDEENIEKLVNEVKIN-------------------------------------------------------------------QVNEPMLKYS--------RENRNELK

Cry_muris_XP_002140093 -----------------------------MLKSFLSAETSREIDQAVEKIKAMQH-------------------------------------------------------------------------EEPVLSSDTR-----LHQIIKS--

Neo_caninum_NCLIV_052980 -------------------MAPTTPSPCLLNNVAGTRLASSAKDASPSTAWLTSPEVLEAATDPKKLSTIVEKYRTPEQKLLSQQMK-------------------------------------EKESEEPLLIANP------RRWVILP--

Tox_gondii_XP_002371991 --------------------MAPTTPSPCLLNDIASSRLGATKEGAQSTAWLTSSEVLDSATDPKKLSAIVEKYRTPEQKLLSEQMK-------------------------------------EKEKEEPLLMANP------HRWVILP--

Dic_discoideum_XP_644369 --------------------------------MEEINKKDTFIEPILKENKD--------------------------------------------------------------------------------------------RFVLFP--

Cae_elegans_NP_497821 --------------MTLTEIQNVEKENAGASVPKHSSNKLKLEKELEKLEIVDQTKAASAEETNNESEVN------------------------------------------------------ELDADEPMLQDLDN------RFVIFP--

Enc_cuniculi_NP_585829 --------------------------------MSHGEETELLLDPKEE------------------------------------------------------------------------------------------------RFVLLP--

Pla_berghei_Pdb_103660 -----------------------------MAEVMNISKSASFSKQEKEFSDFQKTK----------------------------------------------------------------------ESNEKILNKES------NRFTLHP--

Pla_chabaudi_XP_739266 -----------------------------MAEVVNISKSTSFSKQEKEFSDLQKNK----------------------------------------------------------------------ECNEKILNKES------SRFTLHP--

Pla_yoelii_XP_723858 -----------------------------MAEVVNISKSASFSKQEKEFSDFQKSK----------------------------------------------------------------------ESNEKILNKES------NRFTLHP--

Pla_falciparum_XP_001348226 -----------------------------MADVINISRIPIFSKQEREFSDLQKGK----------------------------------------------------------------------EINEKILNKES------DRFTLYP--

Pla_reichenowi_novel_model_330 -----------------------------MADVINISRIPIFSKQEREFSDLQKGK----------------------------------------------------------------------EINEKILNKES------DRFTLYP--

Pla_gallinaceum_rna_PF_0053_1_1cds -----------------------------MADVINISKVPIFTKKEREFSDIQKRK----------------------------------------------------------------------ESNEKILNKES------DRFTLYP--

Pla_knowlesi_XP_002260936 -----------------------------MADVLNISKVPIFSKKEKAFSDLQKSK----------------------------------------------------------------------EANEKILSKET------DRFTLYP--

Pla_vivax_XP_001616894 -----------------------------MADVLNISKIPIFSKKEKKFSDLQKSK----------------------------------------------------------------------EANEKILSKET------DRFTLYP--

Sch_pombe_NP_596546 -----------MGLEHLEEFSYPKEHGEEVEYDSEQGVRKIYVKSIKETFNFDNVSEEEKQEGGDYYLGKKED-----------------------------------------------------ELDEVVLRPNP------HRFVLFP--

Sac_cerevisiae_S288c_NP_011696 ------------------------------MEAHNQFLKTFQKERHDMKEAEKD---------------------------------------------------------------------------EILLMENS------RRFVMFP--

Per_marinus_XP_002768004 --------------------------------------------------------------------------------------------------------------------------------------------------------

Cae_elegans_NP_500944 LYSDPRPAVVPQPDLYGRYHWSQLTPSPPATDSVEPTVTPELTTQNENAGQFSNTVEKSRKPRKKCTKRKVLNDFVSIQLCTT------------------------------------------YTFLEPLLAPNN------NRFVVHP--

Cae_elegans_NP_508269 TAAGFYHDPVIGDHRSPGFQQRCASIYNQGEIQVAANISIDLMNQPRVLLNGCNVKLTVYPNDSKFLIEAYNRDNNTEFQFKITDVYALVNEFDLADGLSNALESSIIEHKIIQYPLISSQVRSFYIESGRLDAPANTLFTSKMPRRIFIGL

Dic_discoideum_XP_629985 -------------------MMNRDREDIEKVLKYFENETEYIIQLLHHSAKLNNEEMFMYIFEKYKHTITDN----------------------------------------------------KIYILINSIFKDNNLKMAQFIFKNFENE

Baci_halodurans_NP_241368 ------------------------------MEQLQKRKIYDTTASNASTGILNGKS----------------------------------------------------------------------------------------SNVLNWDD

Pae_sp_ZP_04851883 ----------------------------MQLQTIFNTEAPNKSTRIIGGECSG---------------------------------------------------------------------------------------------ILNWND

Bact_vulgatus_YP_0130035 -----------------------------MNTIKLKKNALFNPEGDTDLRHRRMIGGN-------------------------------------------------------------------------------------TTNLNDFNN

Clo_botulinum_YP_002805314 ----------------------------MLKKMIFNEKGQRGTESMINGNTTN---------------------------------------------------------------------------------------------LREWNR

Cya_sp_ATCC_51142_YP_001803056 ---------------------------MVVTHQTEMPINPIFNPEGDDAIENRSIWFGN------------------------------------------------------------------------------------TTNLMQLND

Cya_sp_CCY0110_ZP_01726237 ---------------------------MPVTNQTKMSINPIFNPGGDDAIENRSIWFGN------------------------------------------------------------------------------------TTNLMQLND

Cya_sp_ATCC_51142_YP_001806290 ---------------------------MTFTEIKPSPIFNPTGKDNKEKRHLWGG---------------------------------------------------------------------------------------DTTNVINLNE

Cya_sp_CCY0110_ZP_01729893 ---------------------------MTFTEIKPSPIFNPTGKDNKENRHLWGG---------------------------------------------------------------------------------------DTTNVINLNE

Cau_cresents_NP_419079 -----------------------------MSAPSSLILPGLMTPSGGYKP------------------------------------------------------------------------------------------------------

Cau_sp_YP_001686327 -----------------------------MSAKLATLPGLLTPSAAYKP-------------------------------------------------------------------------------------------------------

Neo_sennetsu_YP_506404 --------------------------------MSLLEPRLSYKP------------------------------------------------------------------------------------------------------------

Ori_tsutsugamushi_YP_001248105 --------------------------------MSLLNARPIYKP------------------------------------------------------------------------------------------------------------

Ric_rickettsii_YP_001494766 --------------------------------MSLLDASPIYKP------------------------------------------------------------------------------------------------------------

Wol_pipientis_YP_001974856 --------------------------------MSLLEADPIYKP------------------------------------------------------------------------------------------------------------

Wol_sp_NP_966023 --------------------------------MSLLEADPIYKP------------------------------------------------------------------------------------------------------------

Bau_cicadellinicola_YP_588829 -----------------------------MVYTTFSPKK---NNQLLEPMFLGQS-----------------------------------------------------------------------------------------VNVVRFDQ

Esc_coli_YP_001731173 -----------------------------MAYTTFSQTK---NDQLKEPMFFGQP-----------------------------------------------------------------------------------------VNVARYDQ

Shi_dysenteriae_YP_403993 -----------------------------MAYTTFSQTK---NDQLKEPMFFGQP-----------------------------------------------------------------------------------------VNVARYDQ

Sod_glossinidius_YP_455265 -----------------------------MAYTTFSQVK---NDQLLEPMFFGQS-----------------------------------------------------------------------------------------VNVARFDQ

Yer_pestis_A_ZP_04509308 -----------------------------MAYTTFSQNK---NNQLLEPMFFGQS-----------------------------------------------------------------------------------------VNVARFDQ

Buc_aphidicola_NP_240009 -----------------------------MSYTIFSKKK---NNQLKEPMFFGQP-----------------------------------------------------------------------------------------VNIARYDQ

Yer_pestis_A_ZP_04512133 --------------------------------MTYSTFRLGANDATKEPMFLGQS-----------------------------------------------------------------------------------------VNVARYDQ

Halom_mukohataei_ZP_03875489 -----------------------------MTENKTTGGDTETDIFSERTQLKP---------------------------------------------------------------------------------------------------

Natro_pharaonis_YP_327710 -----------------------------MTESKTTGRDTETDIFSERTQLKP---------------------------------------------------------------------------------------------------

Halor_lacusprofundi_YP_002564382 -----------------------------MTENKTTGGDTATDIFSERTQLKP---------------------------------------------------------------------------------------------------

Chl_muridarum_NP_296594 ------------------------------MQADILDGKQKRVNLNSKRLVNCN-----------------------------------------------------------------------------------------QVDVNQLVP

Chl_trachomatis_YP_328659 ------------------------------MQADILDGKQKRVNLNSKRLVNCN-----------------------------------------------------------------------------------------QVDVNQLVP

Halob_sp_NP_280997 -------------------------------MPVLDSDAEHDPNKILP--------------------------------------------------------------------------------------------------------

Halog_borinquense_ZP_04000565 -------------------------------MAILNNDTEHDPNKILP--------------------------------------------------------------------------------------------------------

Halom_utahensis_YP_003131236 -------------------------------MPIIDTAAEHDPNKILP--------------------------------------------------------------------------------------------------------

Natri_magadii_ZP_03692956 -------------------------------MPIINTDAEHDPNKILP--------------------------------------------------------------------------------------------------------

Geo_kaustophilus_YP_148624 -----------------------------MVHHDGFQTVKATIDWEHP--------------------------------------------------------------------------------------------------------

Geo_sp_ZP_03557705 -----------------------------MVHHDGFQTVKGTIDWEHP--------------------------------------------------------------------------------------------------------

Myc_avium_NP_962606 --------------------------------------MNRTRSASMAQGGLNWDS------------------------------------------------------------------------------------------------

Myc_bovis_NP_853903 --------------------------------------MTRTRSGSLAAGGLNWAS------------------------------------------------------------------------------------------------

Myc_tuberculosis_NP_214747 --------------------------------------MTRTRSGSLAAGGLNWAS------------------------------------------------------------------------------------------------

Nat_pharaonis_YP_331256 -------------------------------MDLDSTRQLPLDRESRG--------------------------------------------------------------------------------------------------------

Nat_pharaonis_YP_330945 -----------------------------MTQIRDDSREMRIDPDSVAG-------------------------------------------------------------------------------------------------------

Sul_islandicus_YP_002913609 -----------------------------MGMSFEEYKHEYFKSIRSGGLN----------------------------------------------------------------------------------------------WSLFP--

Sul_solfataricus_NP_343843 -----------------------------MGMSFEEYKHEYFKSIRSGGLN----------------------------------------------------------------------------------------------WSLFP--

1JK0:A S. cerevisiae Y2 αA α4 αB αC α5 αD

........... ... ....... ..................-----------------------------------------....... ..................--.......... .....------..------.... .......----......

S HHHHHHHHHHH TT GGG TT----HHHHHHH T HHHHHHHHHHHHHHHTTS-----------------------------------------SHHHHHHIIIIITS HHHHHHHHHHHHHHHHHH--HHHHHHHHHH HHHHH------HH------HHHHTT HHHHHHH----HHHHHHTTSSS

**1**SMQ:A S. cerevisiae S HHHHHHHHHHH-TT GGG SH----HHHHHHH--------TS HHHHHHHHHHHHHHTT HHHHTTTSS HHHHHHHHHHHHHHHHHH--HHHHHHHHHH HHHHH------HH------HHTTSS HHHHHHH----HHHHHHTSSSS

1JK0:B S. cerevisiae Y4 S HHHHHHHHHHH-HT GGG TT----TSTTTTT---------S TTHHHHHHHHHHHH HHHHHHHS SHHHHHHHHHHHHHHHHHH--HHHHHHHHHHS S T------TS SGGGG S HHHHHHH----HHHHHTTSSSS

1SMS:A S. cerevisiae S HHHHHHHHHHH-HT GGG SS----SSHHHHS---------S SHHHHHHHHHHHHHHTTH-----------------------------------------HHHHHHHHHHHHHH HHHHHHHHHHHHHHHHHH--HHHHHHHHHHHS GG------G S----STT STT HHHHHHH----HHHHHHSS SS

3HF1:A H. sapiens S HHHHHHHHHHH-HT GGGS SS----HHHHHTT---------S HHHHHHHHHHHHHHHHH HHHHTHHHH HHHHHHHHHHHHHHHHHH--HHHHHHHHHH HHHHH------HH------HHHHTTSHHHHHHH----HHHHHHTTSTT

2UW2:A H. sapiens S HHHHHHHHHHH-HT GGGS GG----GHHHHHT---------S SHHHHHHHHHHHHHHHHH-----------------------------------------HHHHHHHHHTHHHH SHHHHHHHHHHHHHHHHHH--HHHHHHHHHH HHHHT------T TTTGGGT----TTHHHHHH SS-------------------------

2VUX:A H. sapiens S HHHHHHHHHHH-HT GGGS TT HHHHHHS HHHHHHHHHHHHHHHH HHHHHHHH HHHHHHHHHHHHHHHHHH--HHHHHHHHHH S-----------HHHHH------HH------HHHHHHSHHHHHHH----HHHHHHTTTSS

1H0N:A M. musculus HHHHHHHHHHH-HT GGGS TT----HHHHHHH---------S HHHHHHHHHHHHHHTTHH-----------------------------------------HHHHHHHHTTHHHH HHHHHHHHHHHHHHHHHH--HHHHHHHHHH S---------HHHHH------HH------HTTTTT HHHHHHH----HHHHHHHH SS-------------------------

2O1Z:A P. vivax S HHHHHHHHHHH-HT GGG HH----HHHHHHH---------S HHHHHHHHHHHHHHHH HHHHHHH HHHHHHHHHHHHHHHHHH--HHHHHHHHHH HHH------HHH----HHHHHTTSHHHHHHH----HHHHHHSSTTS

2P1I:A P. yoelii HHHHHHHHHHH-TT GGGT GGG SHHHHH---------S TTTHHHHHHHHHHHTT HHHHHHH HHHHHHHHHHHHHHHHHH--HHHHHHHHHHT S-----------HHH------HHH----TTHHHHS HHHHHHH----HHHHHTSS SS

2RCC:A B. halodurans SBTTHHHHHHHHH-HT GGGS HH----HHHHGGG---------S HHHHHHHHHHHHHHHHTT-----------------------------------------TT HHHHHHTTB HHHHHHHHHHHHHHHHHH--HHHHHHHHHHS H----------HHHH------HH------HHHHHT HHHHHHH----HHHHHHHHHHHHS

2ALX:A E. coli S THHHHHHHHHH-HH GGGS HH----HHHHHHH---------S HHHHHHHHHHHHHHHHHH-----------------------------------------HHHHHHHHHHHGGGB SHHHHHHHHHHHHHHHHHH--HHHHHHHHHH S HH------HH------HHHHHH HHHHHHH----HHHHHHHHHHH-HHHHHHHHT SEEEESSS EEEE

1AV8:A E. coli S HHHHHHHHHHH-HH GGGS HH----HHHHHHH---------S HHHHHHHHHHHHHHHHHH-----------------------------------------HHHHHHHHHHTGGGB SHHHHHHHHHHHHHHHHHH--HHHHHHHTTTSS HH------HH------HHHHHH HHHHHHH----HHHHHHHHHHH-HHHHHHHHH SEEEEETTEEEEE

1JPR:A E. coli S HHHHHHHHHHH-HT GGGS HH----HHHHHHH---------S HHHHHHHHHHHHHHHHHH-----------------------------------------HHHHHHHHHHTGGGB SHHHHHHHHHHHHHHHHHH--HHHHHHHHHH S HH------HH------HHHHHH HHHHHHH----TTHHHHHHHHH-HHHHHHHHH SEEEEETTEEEEE

1JQC:A E. coli S HHHHHHHHHHH-HT GGGS HH----HHHHHHH---------S HHHHHHHHHHHHHHHHHH-----------------------------------------HHHHHHHHHHTGGGB SHHHHHHHHHHHHHHHHHH--HHHHHHHHTT S HH------HH------HHHHHH HHHHHHH----TTHHHHHHHHH-HHHHHHHHH SEEEEETTEEEEE

1MXR:A E. coli S HHHHHHHHHHH-HT GGGS TT----HHHHHHT---------S HHHHHHHHHHHHHHHHHH-----------------------------------------HHHHHHHHHHTGGGB SHHHHHHHHHHHHHHHHHH--HHHHHHHHTT S HH------HH------HHHHHH HHHHHHH----TTHHHHHHHHH-HHHHHHHHH SEEEEETTEEEEE

1SYY:A C. trachomatis HHHHHHHHHHH-HT GGGS HH----HHHHHHSS S HHHHHHHHHHHHHHHHHH-----------------------------------------HHHHHHHHHTHHHH HHHHHHHHHHHHHHHHHH--HHHHHHHHHHT HH------HH------TTHHHH HHHHHHH----HHHHHTTSGGGSTT SSS-----------------

2ANI:A C. trachomatis HHHHHHHHHHH-HT GGGS HH----HHHHHHSS S HHHHHHHHHHHHHHHHHH-----------------------------------------HHHHHHHHHTHHHH HHHHHHHHHHHHHHHHHH--HHHHHHHHHHT HH------HH------HTHHHH HHHHHHH----HHHHHHHTGGGSTT SSS-----------------

3EE4:A M. tuberculosis ----HHHHHHHHHH-HT GGGS TT----HHHHHHH---------S HHHHHHHHHHHHHHHHHH-----------------------------------------HHHHHHTHH-HHHHHHH---------------TT HHHHHHHHHHHHHHHHHH--HHHHHHHHHHT S GGGGTT HHHHHHHH---THHHHHHHHHHH

S. cerevisiae S288c NP 012508 (Y2) 100 110 120 130 140 150 160 170 180 190 200 210 220

coordinates . . . . . . . . . . . . .

matrix position 380 390 400 410 420 430 440 450 460 470 480 490 500 510 520 530 540 550 560 570 580 590 600 610 620 630

. . . . . . . . . . . . . . . . . . . . . . . . . .

E. coli electron transfer pathway ⧫

C. trachomatis electron transfer pathway ❖ ❖

S. typhimurium electron transfer pathway ◊ ◊ ◊ ◊

diiron center residues ⚫ ⚫ ⚫

conserved residues (Wang 1997) ↓ ↓ ↓ ↓ ↓

Högbom 2010 positions 1 * 2 | 3 45 6 7 891011 12 13

Högbom 2010 exceptions | * * | | * *| | | |

Bab_bovis_XP_001610573 IHYDALWAMYKEIE-NSFWAAEDFRFAN----ERDTYSS---------LPTELRQLVVKLISYHNRLDRS--------------------------------------EVARPATITLDLLADTQ------------------IPEARAFYGFQVSYENIHS--ELFGIMSSAIPGT--------------------------------IDVPEADAKI----EWLTRNLTSTS-------------------------

Bab_equi_6m007985 IKHDSFWAMYKEVE-NNFWAAEDFIFSE----DKEVLDR---------LDKVLLDALNKVLSYHIMLDNN--------------------------------------VKCRPSAITLDLLSDVQ------------------VPEARAFYGFQLTDENIHS--ETVGSMFQTLAAS-------------------------------LDSKSGSDKIS-----WLHKNVIETK-------------------------

The_annulata_XP_953574 IEHDTFWILYKEVE-NNFWAAEDFIFTD----DIKLLYQ---------LPKPIFNTLLNLLSFHINLDN--------------------------------------KLICRPVDITLDLLSEVQ------------------IAEARAFYGFQLTDENIHT--ETISSMFQLFNQQ----------LDTN---------------IVSSNTLFLSSYGMDKIIWLYNECENNK-------------------------

The_parva_XP_766717 IEHDTFWIMYKEVE-NNFWAAEDFIFSD----DLNSLFK---------LPKPLFNSLFHLLSFHINLDN--------------------------------------KWVCRPVDMTLELLSEVQ------------------IAEARAFYGFQLTDENIHT--ETIGTMFQLLNQP-------------------------------LDNTIGQDKIT-----WLYNECEKNK-------------------------

Pla_berghei_Pdb_121420 IKYKTFWNYYKEIE-SLFWTAEDHNFDK----DKPFLNS---------MDKITLSKLLELLCFYSLKD-----------------------------------------LHLCDEQAIITSKLLD---------------IIQIPEGRAFYGFQMCMENIHN--EVYACIFESYSSDS--------KQKKE-------------IIDKVLKYESVFKKQ----NWLYSEFEANIP------------------------

Pla_yoelii_XP_727957 IKYKTFWNYYKEIE-SLFWTAEDHNFDK----DKPFLNS---------MDKTTLSKLLELFCFYSLKD-----------------------------------------LHLRDEQAIITSKLLD---------------IIQIPEGRAFYGFQMCMENIHN--EVYACIFESYSLDS--------KQKKE-------------IIDKVLKYESVFKKQ----NWLYNEFEANIP------------------------

Pla_chabaudi_PCAS_121490 IKYKTFWNYYKEIE-SLFWTAEDHNFDK----DKPFLSS---------MDQTMLSKLLELLCFYSLKD-----------------------------------------LHLRDEQAIITSKLLD---------------IIQIPEGRAFYGFQMCMENIHN--EVYACIFESYSSDS--------KKKKE-------------IIDQVLKYESVFKKQ----NWLYNEFEANIP------------------------

Pla_falciparum_XP_001347439 IKYKTFWSYYKEIE-SLFWTAEDYNFDK----DKQYLEN---------IDKNMLVKLFELICFYSLKD-----------------------------------------LHVYEEQALITSKMLD---------------IIQIPEGRAFYGFQMCMENIHD--EVYACIFETYIPD---------SKQKR------------VIINKVIELDSVLKKQ----KWLTEIFESNIP------------------------

Pla_knowlesi_XP_002258799 IKYKTFWSYYKEVE-SLFWTAEDYNFDK----DKKFLES---------IDKNMLVKLFELICFYSLKD-----------------------------------------LHVYEEQALITSKMLD---------------IIQIPEGRAFYGFQMCMENIHD--EVYACIFETYIPD---------AKQKR------------TIINKVIQLDSVLKKQ----KWLTELFETNIP------------------------

Pla_vivax_XP_001614470 IKYKTFWSYYKEIE-SLFWTAEDYNFDK----DKKFLES---------IDKNMLVKLFELICFYSLKD-----------------------------------------LHVYEEQALITSKMLD---------------IIQIPEGRAFYGFQMCMENIHD--EVYACIFETYIPD---------AKQKR------------VIINKVIQLDSVLKKQ----KWLTELFETNIP------------------------

Cry_hominis_XP_665685 IKYSRLWSLYKKYE-VSFWTAEVISPME----DKTKLMN---------MDERLLDYIEMVFAKRIYIDN---------------------------------------TCLDTVLLTCELLGQVQ------------------VPEARGYFSFTMCMENIYK--ELFMNYVEMLRKNKEKNEKTDEVEKKDENNLNSKISKEEYEYNLEKKLNELEDFTKMK-ELVGNFYDEDT-------------------------

Cry_parvum_XP_001388304 IKYSRLWSLYKKYE-VSFWTAEVISPME----DKTKLMN---------MDEKLLDYIEMAFAKRIYIDN---------------------------------------TCLDTVLLTCELLGQVQ------------------VPEARGYFSFTMCMENIYK--ELFMNYVEMIRKNKEKIERTDEVEKKDENNLNSKISKEEYEYNLEKKLNELEDFTKMK-ELVGNFYDEDT-------------------------

Cry_muris_XP_002140092 IKYNRLWNFYKKYE-VVFWTAEVISYIK----DLG---------IISEVDRRFVDSIELLFSSRVFISN---------------------------------------TGLDSVLVACEFLSQIQ------------------VPEARGYFSFTICMENIYK--EVFMNYIDVIRKYKS-------ENIKEYD----HKEAKMRYHLKLKDIITDNQHYQRKIEFANQLLDSSVP------------------------

*.:. :* **: * **:** . : : : :. . : : :.*.*.::.* : ***: * . .

Högbom 2010 positions 1 * 2 | 3 45 6 7 891011 12 13

Högbom 2010 exceptions | * | | | * *| | | |

Ano_gambiae_XP_308927 IQYPDIWQMYKKAE-ASFWTVEEVDLSK----DMKDWET---------LKPSERHFISHVLAFFAASD-----------------------------------------GIVNENLVERFSQEVQ------------------VTEARCFYGFQIAMENVHS--EMYSLLIDTYIRD---------SKERE------FL------FNAIETLPCVKRKA----DWALNWISSKK-------------------------

Dro_melanogaster_NP_525111 IQYHDIWQMYKKAE-ASFWTVEEVDLSK----DLTDWHR---------LKDDERHFISHVLAFFAASD-----------------------------------------GIVNENLVERFSQEVQ------------------ITEARCFYGFQIAMENVHS--EMYSVLIDTYIRD---------PHQRE------YL------FNAIETMPAVKRKA----DWALSWISSKS-------------------------

Dan_rerio_NP_571525 IQYHDIWQMYKKAE-ASFWTAEEVDLSK----DLQHWDS---------LKDEERYFISHVLAFFAASD-----------------------------------------GIVNENLVERFTQEVQ------------------VTEARCFYGFQIAMENIHS--EMYSLLIDTYIKD---------SKERE------FL------FNAIETMPCVKKKA----DWALNWIGDKN-------------------------

Gal_gallus_XP_001231545 IQYHDIWQMYKKAE-ASFWTAEEVDLSK----DLQHWES---------LKPEEKYFISHVLAFFAASD-----------------------------------------GIVNENLVERFSQEVQ------------------VTEARCFYGFQIAMENIHS--EMYSLLIDTYIKD---------SKERE------FL------FNAIETLPCVKKKA----DWAIRWIGDKK-------------------------

Hom_sapiens_isoform_2_NP_001025 IEYHDIWQMYKKAE-ASFWTAEEVDLSK----DIQHWES---------LKPEERYFISHVLAFFAASD-----------------------------------------GIVNENLVERFSQEVQ------------------ITEARCFYGFQIAMENIHS--EMYSLLIDTYIKD---------PKERE------FL------FNAIETMPCVKKKA----DWALRWIGDKE-------------------------

Mus_musculus_NP_033130 IEYHDIWQMYKKAE-ASFWTAEEVDLSK----DIQHWEA---------LKPDERHFISHVLAFFAASD-----------------------------------------GIVNENLVERFSQEVQ------------------VTEARCFYGFQIAMENIHS--EMYSLLIDTYIKD---------PKERE------YL------FNAIETMPCVKKKA----DWALRWIGDKE-------------------------

Rat_norvegicus_NP_001020911 IEYHDIWQMYKKAE-ASFWTAEEVDLSK----DIQHWEA---------LKPDERHFISHVLAFFAASD-----------------------------------------GIVNENLVERFSQEVQ------------------VTEARCFYGFQIAMENIHS--EMYSLLIDTYIKD---------SKERE------YL------FNAIETMPCVKKKA----DWALRWIGDKE-------------------------

Xen_laevis_NP_001079369 IQYHDIWQMYKKAE-ASFWTAEEVDLSK----DLQHWES---------LKKEEKYFISHVLAFFAASD-----------------------------------------GIVNENLVERFSKEVQ------------------VTEARCFYGFQIAMENIHS--EMYSLLIDTYVKD---------PKERE------YL------FNAIETLPCVKKKA----DWALHWIGDKQ-------------------------

Xen_Silurana_tropicalis_NP_001007890 IQYHDIWQMYKKAE-ASFWTAEEVDLSK----DLRHWES---------LKAEEKYFISHVLAFFAASD-----------------------------------------GIVNENLVERFSKEVQ------------------VTEARCFYGFQIAMENIHS--EMYSLLIDTYIKD---------PKERE------YL------FNAIETLPCVKKKA----DWALRWIGDKQ-------------------------

Xen_laevis_NP_001080772 IQYHDIWQMYKKAE-ASFWTAEEVDLSK----DIQHWES---------LKTDERYFISHVLAFFAASD-----------------------------------------GIVNENLVERFSKEVQ------------------VTEARCFYGFQIAMENIHS--EMYSLLIDTYIKD---------PKERG------FL------FNAIETLPCVKKKA----DWALRWISDKQ-------------------------

Xen_laevis_NP_001085389 IEYHDIWQMYKKAE-ASFWTAEEVDLSK----DLQHWET---------LKPEERYFIAYVLAFFAASD-----------------------------------------GIVNENLVERFSQEVQ------------------VTEVRCFYGFQIAMENIHS--EMYSLLIDTYIKD---------PKERE------FL------FNAIETLPCVKKKA----EWSLRWISDRE-------------------------

Xen_Silurana_tropicalis_NP_989048 IEYHDIWQMYKKAE-ASFWTAEEVDLSK----DLPHWEA---------LKPEERYFISYVLAFFAASD-----------------------------------------GIVNENLVERFSQEVQ------------------VTEARCFYGFQIAMENIHS--EMYSLLIDTYIKD---------PKERE------FL------FNAIETLPCVKKKA----EWALRWISDRE-------------------------

Gal_gallus_XP_418364 IQHPDIWKMYKQAQ-ASFWTAEEVDLSK----DLPHWNK---------LKADEKYFISHVLAFFAASD-----------------------------------------GIVNENLVARFSQEVQ------------------IPEARCFYGFQILIENVHS--EMYSLLIDTYIKD---------PEKRD------FL------FNAIETMPCVKKKA----DWALKWIEDRE-------------------------

Hom_sapiens_isoform_1_NP_056528 IQYPDIWKMYKQAQ-ASFWTAEEVDLSK----DLPHWNK---------LKADEKYFISHILAFFAASD-----------------------------------------GIVNENLVERFSQEVQ------------------VPEARCFYGFQILIENVHS--EMYSLLIDTYIRD---------PKKRE------FL------FNAIETMPYVKKKA----DWALRWIADRK-------------------------

Mus_musculus_NP_955770 IQYPDIWRMYKQAQ-ASFWTAEEVDLSK----DLPHWNK---------LKSDEKYFISHILAFFAASD-----------------------------------------GIVNENLVERFSQEVQ------------------VPEARCFYGFQILIENVHS--EMYSLLIDTYIRD---------PKKRE------FL------FNAIETMPYVKKKA----DWALRWIADRK-------------------------

Rat_norvegicus_NP_001124015 IQYPDIWKMYKQAQ-ASFWTAEEVDLSK----DLPHWNK---------LKSEEKYFISHILAFFAASD-----------------------------------------GIVNENLVERFSQEVQ------------------VPEARCFYGFQILIENVHS--EMYSLLIDTYIRD---------PKKRE------FL------FNAIETMPYVKKKA----DWALRWIADRK-------------------------

Xen_Silurana_tropicalis_NP_001119973 IHYPDIWKMYKKAQ-ASFWTAEEVDLSK----DLVHWEK---------LKPEERNFISHILAFFAASD-----------------------------------------GIVNENLVERFSQEVQ------------------VPEARCFYGFQILIENVHS--EMYSLLIETYIKD---------PRRRE------FL------FNAIETMPCVRKKA----QWALRWISDRK-------------------------

Dan_rerio_NP_001007164 IQYPDIWKMYKQAQ-ASFWTVEEVDLSK----DLTHWDG---------LKSEEKHFISHVLAFFAASD-----------------------------------------GIVNENLVQRFSQEVQ------------------LPEARSFYGFQILIENVHS--EMYSMLINTYIRD---------LKERD------YL------FNAVQTMPCVRRKA----DWALQWISDTN-------------------------

Dap_pulex_GNO_1472053 IQYHDIWQMYKKAE-ASFWTAEEVDLSK----DLKDWDN---------LQPNERHFVSHVLAFFAASD-----------------------------------------GIVNENLVERFAQEVQ------------------VPEARCFYGFQIAMENIHS--EMYSLLIETYIKD---------SAERT------RL------FNAVETMPCIAKKA----EWAMKWIGNKD-------------------------

Asp_clavatus_XP_001274524 IKYHEIWQMYKKAE-ASFWTAEEIDLSK----DLHDWNN--------RLNDDERYFISHVLAFFAASD-----------------------------------------GIVNENLLERFSNEVQ------------------VPEARCFYGFQIMIENIHS--ETYSLLIDTYIKE---------PKQRA------YL------FDAIDTIPCINKKA----QWAMRWISDKE-------------------------

Neu_crassa_XP_962820 IKYHEIWQMYKKAE-ASFWTAEEIDLSK----DLHDWNN--------RLNDDEKFFISHILAFFAASD-----------------------------------------GIVNENLVERFSGEVQ------------------IPEARCFYGFQIMMENIHS--ETYSLLIDTYIKE---------PSQRT------YL------FNAIDTIPCIRKKA----DWALRWITDKS-------------------------

Dap_pulex_GNO_1331594 IKHHDIWEWYKKME-ASFWTAEEIDLSQ----DLNDWNN--------KLSEDEKYFIKHILAFFAASD-----------------------------------------GIVNENLAENFVNEVQ------------------YAEAKFFYGFQIMMENIHS--ETYSLLIDTYVKD---------ESEKN------EL------FTAIEVFPAIKKKA----DWALKWIESDS-------------------------

Can_albicans_XP_715277 IRYHEIWNFYKKAE-ASFWTAEEIDLSK----DLDDWNN--------KLNENERYFISRVLAFFAASD-----------------------------------------GIVGENLIENFSTEVQ------------------LPEAKSFYGFQIMMENIHS--ETYSLLIETYIKD---------PQEAD------YL------FNAIANIPCIQKKA----DWAIKWIQDDE-------------------------

Can_albicans_XP_713125 IKYPELWQFYKKSL-ASFWTAEELDLSK----DLDDWNN--------KMNENERFFISRVLAFFAASD-----------------------------------------GIVNENLVENFCAEVQ------------------IPEAKSVYKFQIMMENIHS--ETYSLLIETYFKD---------PEEAD------FL------FNAIDNIPFIRKKA----DWAIRWIQSED-------------------------

Sac_cerevisiae_S288c_NP_012508 IKYHEIWQAYKRAE-ASFWTAEEIDLSK----DIHDWNN--------RMNENERFFISRVLAFFAASD-----------------------------------------GIVNENLVENFSTEVQ------------------IPEAKSFYGFQIMIENIHS--ETYSLLIDTYIKD---------PKESE------FL------FNAIHTIPEIGEKA----EWALRWIQDAD-------------------------

Lei_braziliensis_XP_001565976 IQYHDIWRKYKEQE-SCIWTVEEIDLGN----DMKDWVA---------LNDGERHFIKHVLAFFAGSD-----------------------------------------GIVVENLAQRFMSDVK------------------VPEARAFYGFQLMMENIHS--ETYSVLLDTYITD-----------SEE------KLR----LLHAIQTIPCIQKKA----EWAVRWIGSSA-------------------------

Lei_braziliensis_XP_001565036 IQYHDIWRKYKEQE-SCIWTVEEIDLGN----DMKDWVA---------LNDGERHFIKHVLAFFAGSD-----------------------------------------GIVVENLAQRFMSDVK------------------VPEARAFYGFQLMMENIHS--ETYSVLLDTYITD-----------SEE------KLR----LLHAIQTIPCIQKKA----EWAVRWIGSSA-------------------------

Try_cruzi_XP_813233 IKYHDIWKKYKESE-SSIWTVEEIDLSS----DLADWEK---------MNEGEKFFIKHVLAFFAASD-----------------------------------------GIVLENLAQRFMTEVQ------------------VPEVRCFYGFQIAIENVHS--ETYSVLLDTYITD---------TEEKD------RM------LHAISTIPCIQKKA----NWAIRWIRSSS-------------------------

Par_tetraurelia_XP_001454302 IKYNDIWNMYKQHK-ASFWTAEEIDLYQ----DLKDWEK---------LTADEKHFIKYVLAFFAASD-----------------------------------------GIVNENLAQQFCTEVQ------------------VPEARCFYGFQIAMENIHS--ETYSLLIDTYITD---------ENEKN------YL------LHAIDNVPVIQKKA----MWAMKWINEND-------------------------

Tet_thermophila_XP_001024960 IKYNKVWEMYKKEL-ASFWTADEIDLYQ----DLKDWER---------LTDNERHFIKYVLAFFAASD-----------------------------------------GIVLENLAEQFMCEVQ------------------IPEARAFYGFQIAMENIHS--ETYSLLIDTYIKD---------EVEKD------YL------FKASQNVPVIHKKA----QWALKWINNND-------------------------

Ara_thaliana_NP_189000 IRYKSIWEMYKKAE-ASFWTAEEVDLST----DVQQWEA---------LTDSEKHFISHILAFFAASD-----------------------------------------GIVLENLAARFLNDVQ------------------VPEARAFYGFQIAMENIHS--EMYSLLLETFIKD---------SKEKD------RL------FNAIETIPCISKKA----KWCLDWIQSPM-------------------------

Pop_trichocarpa_EEE89193 IRYKELWEMYKKAE-ASFWTAEEVDLSR----DMQQWEA---------LSDSEKHFISHVLAFFAASD-----------------------------------------GIVLENLAARFLYDVQ------------------IPEARAFYGFQIAMENIHS--EMYSLLLETYIKD---------SREKH------RL------FNAIENIPCVAEKA----KWALDWIQSSM-------------------------

Ara_thaliana_NP_189342 IHYPQIWEMYKKAE-ASFWTAEEVDLSQ----DNRDWEN--------SLNDGERHFIKHVLAFFAASD-----------------------------------------GIVLENLASRFMSDVQ------------------VSEARAFYGFQIAIENIHS--EMYSLLLDTYIKD---------NKERD------HL------FRAIETIPCVAKKA----QWAMKWIDGSQ-------------------------

Pop_trichocarpa_EEE77642 IQYPSIWEMYKKAE-ASFWTAEEVDLSS----DIRHWEN---------LTPDEKHFISHVLAFFAASD-----------------------------------------GIVLENLAGRFMKEVQ------------------VSEARAFYGFQIAIENIHS--EMYSLLLETYIKD---------SEEKN------RL------FHAIETVPCVAKKA----RWALRWIDGSE-------------------------

Pop_trichocarpa_EEE83435 IQYPSIWEMYKKAE-ASFWTAEEVDLSS----DVGHWEN---------LTPDEKHFISHVLAFFAASD-----------------------------------------GIVLENIAGRFMKEVQ------------------VSEARAFYGFQIAIENIHS--EMYSLLLETYIKD---------STEKN------RL------FHAIETVPCVAKKA----EWALRWIDGGE-------------------------

Ory_sativa_ACC95435 IRYPQIWEFYKKAV-ASFWTAEEVDLSA----DARHWDAA--------LSPDERHFISHVLAFFAASD-----------------------------------------GIVLENLASRFMSDVQ------------------VAEARAFYGFQIAIENIHS--EMYSLLLETYIRD---------GAEKD------RL------FRAIDTVPAVRRKA----DWAMRWIDGGE-------------------------

Zea_mays_NP_001130908 IRYPQIWEFYKKAV-ASFWTAEEVDLSA----DARHWDAA--------LSPDERHFISHVLAFFAASD-----------------------------------------GIVLENLASRFMSDVQ------------------AAEARAFYGFQIAIENIHS--EMYSLLLETYIRD---------GTEKD------RL------FRAIETVPAVRRKA----DWAMRWIDGGE-------------------------

Zea_mays_NP_001131892 IRFPQIWEFYKKAV-ASFWTAEEVDLSA----DARHWDEA--------LSPDERHFISHVLAFFAASD-----------------------------------------GIVLENLASRFMSDVQ------------------VAEARAFYGFQIAIENIHS--EMYSLLLETYIRD---------DVEKD------RL------FRAIDTVPAVRRKA----DWAMRWIDGGE-------------------------

Zea_mays_NP_001150842 IRFPQIWEFYKKAV-ASFWTAEEVGLSA----DARHWDEA--------LSPDERHFISHVLAFFAASD-----------------------------------------GIVLENLASRFMTDVQ------------------VAEARAFYGFQIAIENIHS--EMYSLLLETYIRD---------HVEKD------RL------FRAIDTVPAVRRKA----DWAMRWIDGGE-------------------------

Ory_sativa_NP_001056668 IRYPQIWEFYKKAV-ASFWTAEEVDLSA----DARHWDAA--------LSPDERHFVSHVLAFFAASD-----------------------------------------GIVLENLASRFMSDVQ------------------VAEARAFYGFQIAIENIHS--EMYSLLLETYIRD---------DVEKD------RL------FRAIDTVPAVRRKA----DWAMRWIDGGE-------------------------

Per_marinus_XP_002786498 IKYLAIWEMYKKHE-ASFWTAEEIDLSQ----DLRDWEN---------LSDNDRHFISHVLAFFAASD-----------------------------------------GIVLENLSAKFSGEVQ------------------CPEARAFYGFQIAMENIHS--ETYSLLIDNYIKD---------PEQKD------KI------FRAIETVPSVRKKA----EWALSWINDDN-------------------------

Per_marinus_XP_002773236 IKYLAIWEMYKKHE-ASFWTAEEIDLSQ----DLRDWEN---------LSDNDRHFISHVLAFFAASD-----------------------------------------GIVLENLSAKFSGEVQ------------------CPEARAFYGFQIAMENIHS--ETYSLLIDNYIKD---------PAEKD------KI------FRAIETVPSVRKKA----EWALSWINDDN-------------------------

Bab_bovis_XP_001610982 IKYPDFWEWYKKAQ-ASFWTSEEIDLSS----DLKDWGT---------LTEGEHHFIKNVLAFFAASD-----------------------------------------GIVLENLALRFLKDVK------------------LPEAKFFYCFQITVENIHS--ETYSLLIEQYIRD---------EAEKD------RL------FRAIETIDAVRDKA----IWAAKWMNDEK-------------------------

Bab_equi_6m007342 IKFPDFWEWYKKAQ-ASFWTAEEIDFSL----DLAHWNK---------LTSDERHFISNVLAFFAASD-----------------------------------------GIVLENLALKFLRDVK------------------LPEAQSFYSFQIAVENIHS--ETYSLLIENYIKD---------EAERT------RL------FRAIDTIQAVKDKA----NWAAKWITENN-------------------------

The_annulata_XP_954052 IVYPDFWEWYKKAQ-ASFWTAEEIDFSM----DYNHWHK---------LNKDERHFITNVLAFFAASD-----------------------------------------GIVLENLALKFLRDVK------------------IPEAQSFYSFQIAVENIHS--ETYSLLIENYVKD---------EAEKR------RL------FMAIETIDAVKDKA----NWAKKWITDEN-------------------------

The_parva_XP_766246 IVYPEFWEWYKKAQ-ASFWTAEEIDFSM----DYNHWHR---------LNKDERHFITNVLAFFAASD-----------------------------------------GIVLENLALKFLRDVK------------------IPEAQSFYSFQIAVENIHS--ETYSLLIENYVKD---------EAEKR------RL------FMAIETIDAVKDKA----NWAKKWITDEN-------------------------

Cry_hominis_XP_665115 --------MYKKAE-ASFWVTEEIDLSQ----DTRDWES---------LKDPERHFIKYVLAFFAASD-----------------------------------------GIVMENLAVNFLREIQ------------------IPEARMYYAFQMSIEQIHS--ETYSLLIDRYITD---------IKERQ------ML------FEAISHIEAVKKKA----EWATKWMNSER-------------------------

Cry_parvum_XP_627447 ELIKDIWSMYKKAE-ASFWVTEEIDLSQ----DTRDWES---------LKDPERHFIKYVLAFFAASD-----------------------------------------GIVMENLAVNFLREIQ------------------IPEARMYYAFQMSIEQIHS--ETYSLLIDRYITE---------IKERQ------ML------FEAISHIEAVKKKA----EWATKWMNSER-------------------------

Cry_muris_XP_002140093 -EIEVMWSMYKKEE-ASFWTAEEIDLGQ----DMRDWES---------LKEPEKHFIKYVLAFFAASD-----------------------------------------GIVMENLAVNFLKEIQ------------------IEEARMYYGFQIAMEQIHS--ETYSLLIDQYISD---------VKDRQ------ML------FEAISHLPAVKTKA----KWATKWMNNNR-------------------------

Neo_caninum_NCLIV_052980 IQHHAIWEMYKKQE-ASFWTAEEIDLAQ----DMTHWES---------LNSNEKHFIKYVLAFFAASD-----------------------------------------GIVLENLAEKFLSEIQ------------------IPEARAFYGFQIAMENIHS--ETYSLLIDQYIRD-----------EKE------KMEL----FDAVHNVKAVAVKA----AWAAMWINNRN-------------------------

Tox_gondii_XP_002371991 IQHHAIWEMYKKQE-ASFWTAEEIDLAQ----DMTHWET---------LGENEKHFIKYVLAFFAASD-----------------------------------------GIVLENLAEKFLTEIQ------------------VPEARAFYGFQIAMENIHS--ETYSLLIDQYIRD-----------EEE------KLQ----LFDAVHNVKAVAVKA----AWAAMWINNRN-------------------------

Dic_discoideum_XP_644369 IKYPDIWRMYKKAL-ASHWVAEEIDLGN----DNVDWEYK--------LTDNERHFISHVLAFFAASD-----------------------------------------GIVNENLATRFMSEVQ------------------IPEARCFYGFQIAIENIHS--ETYSLLIETYIKD---------KQTKD------KL------FNAIETIPCIKKKA----EWALRWINDSD-------------------------

Cae_elegans_NP_497821 LKHHDIWNFYKKAV-ASFWTVEEVDLGK----DMNDWEK---------MNGDEQYFISRILAFFAASD-----------------------------------------GIVNENLCERFSNEVQ------------------VSEARFFYGFQIAIENIHS--EMYSKLIETYIRD---------ETERN------TL------FNAVDEFEFIKKKA----DWALRWISDKK-------------------------

Enc_cuniculi_NP_585829 IKYHDIWKMYKKAE-SSFWTVEEVSLDK----DIDDWGK---------LNAKERHFISYVLAFFAASD-----------------------------------------GIVNLNLVERFSTEVK------------------VLEARFFYGFQMAIENIHS--EMYSLLIDTYIRD---------NDEKN------FL------FDAIRTIPSVKEKA----DWAIRWIEDKN-------------------------

Pla_berghei_Pdb_103660 IIYPEVWNFYKKAE-ASFWTAEEIDLSS----DLKDFEK---------LNVNEKHFIKHVLAFFAASD-----------------------------------------GIVLENLASKFLREVE------------------IIEAKKFYSFQIAVENIHS--ETYSLLIDNYIKD-----------EKE------RLN----LFHAIENIPAIKNKA----LWAAKWINDTN-------------------------

Pla_chabaudi_XP_739266 IMYPEVWNFYKKAE-ASFWTAEEIDLSS----DLKDFEK---------LNVNEKHFIKHVLAFFAASD-----------------------------------------GIVLENLASKFLREVQ------------------IIEAKKFYAFQIAVENIHS--ETYSLLIDNYIRD-----------EKE------RLN----LFHAIENIPAIKNKA----LWAAKWINDTN-------------------------

Pla_yoelii_XP_723858 IMYPEVWNFYKKAE-ASFWTAEEIDLSS----DLKDFEK---------LNVNEKHFIKHVLAFFAASD-----------------------------------------GIVLENLASKFLREVE------------------IIEAKKFYSFQIAVENIHS--ETYSLLIDNYIKD-----------EKE------RLN----LFHAIENIPAIKNKA----LWAAKWINDTN-------------------------

Pla_falciparum_XP_001348226 ILYPDVWDFYKKAE-ASFWTAEEIDLSS----DLKDFEK---------LNENEKHFIKHVLAFFAASD-----------------------------------------GIVLENLASKFLREVQ------------------ITEAKKFYSFQIAVENIHS--ETYSLLIDNYIKD-----------EKE------RLN----LFHAIENIPAVKNKA----LWAAKWINDTN-------------------------

Pla_reichenowi_novel_model_330 ILYPDVWDFYKKAE-ASFWTAEEIDLSS----DLKDFEK---------LNENEKHFIKHVLAFFAASD-----------------------------------------GIVLENLASKFLREVQ------------------ITEAKKFYSFQIAVENIHS--ETYSLLIDNYIKD-----------EKE------RLN----LFHAIENIPAVKNKA----LWAAKWINDTN-------------------------

Pla_gallinaceum_rna_PF_0053_1_1cds ILYPDVWEFYKKAE-ASFWTAEEIDLSS----DLKDFEK---------LNENEKHFIKYVLAFFAASD-----------------------------------------GIVLENLASKFLREVQ------------------ITEAKKFYSFQIAVENIHS--ETYSLLIDNYIRD-----------EKE------RLN----LFHAIENIPAVKNKA----LWAAKWINDTN-------------------------

Pla_knowlesi_XP_002260936 ILYPDVWDFYKKAE-ASFWTAEEIDLSS----DLKDFEK---------LNENEKHFIKHVLAFFAASD-----------------------------------------GIVLENLASKFLRQVQ------------------ITEAKKFYSFQIAVENIHS--ETYSLLIDNYIKD-----------EKE------RMN----LFHAIENIPAVKNKA----LWAAKWINDTN-------------------------

Pla_vivax_XP_001616894 ILYPDVWDFYKKAE-ASFWTAEEIDLSS----DLKDFEK---------LNDNEKHFIKHVLAFFAASD-----------------------------------------GIVLENLASKFLRQVK------------------ITEAKKFYAFQIAVENIHS--ETYSLLIDNYIKD-----------EKE------RMN----LFHAIENIPAVKNKA----LWAAKWINDTN-------------------------

Sch_pombe_NP_596546 IKYHEIWQFYKKAE-ASFWTAEEIDLSK----DLVDWDN--------KLNADERYFISTVLAYFAASD-----------------------------------------GIVNENLLERFSSEVQ------------------IPEARCVYGFQIMIENIHS--ETYSLLLDTYIRE---------PKEKQRH------------FDAILTMGSIKAKA----KWALRWINDED-------------------------

Sac_cerevisiae_S288c_NP_011696 IKYHEIWAAYKKVE-ASFWTAEEIELAK----DTEDFQK---------LTDDQKTYIGNLLALSISSD-----------------------------------------NLVNKYLIENFSAQLQ------------------NPEGKSFYGFQIMMENIYS--EVYSMMVDAFFKD------------PK------NIP----LFKEIANLPEVKHKA----AFIERWISNDD-------------------------

Per_marinus_XP_002768004 -MNCLVNCGYKSPE-GSYN-----NTST---------------------------------------------------------------------------------------------------------------------------------ETKHYREALYATHAESNTFIEDAQ-------EKE------RI------FEAIETIPSVQHKA----EWALAWINDDN-------------------------

Cae_elegans_NP_500944 IVHRDIWEFYKKAV-ACFWTSEEVDLGK----DMSHWEI---------LTSDERQFISSILAFFAASD-----------------------------------------GIVTENLCSRFSTEVQ------------------VTEARFFYGFQIAVENIHS--EMYAKLLEAYIRD---------DAERN------IL------FNAITTFKFIKAKA----DWCLRWISDHN-------------------------

Cae_elegans_NP_508269 VDSDAYNGSYDKSP-FNFKPHGISDIHV----DYCGMTLPGRPF---ALDFDRNKFMEAYIQLQETLGHSRSNSTCNSISTQMFKEGGYTIFGFELSPVAQDTSLFELVRQTNVSIRLNFREKVPVGGLYCIVYAEFDQIFSLDFMRNPIVDTIIAVENIHS--EMYAKLLEAYIRD---------DAERN------------ILFNAITTFKFIKAKA----DWCLRWISDHN-------------------------

Dic_discoideum_XP_629985 ISPLDIWKMYKKTL-ANQWVVEEIESSD----DGLDWEKK--------LTNDERDFFSNVLAFFVAS-----------------------------------------CGIINKNLN-RFMSKVQ------------------IPEAKCFYNYQIHIKYLHS--ETYSLLIETCIKD---------KNIKD------------KLFNAIETIPCVKKKA----EWALKWINESF-------------------------

*.. * : . :. : : .* .: . : :: ::* * *: ::: . : : . : *: : *:

(Hom_sapiens_B_isoform_3_NP_001165949 & Per_marinus_XP_002768004 excluded)

Högbom 2010 positions 1 * 2 | 3 45 6 7 891011 12 13

Högbom 2010 exceptions | * | | | * *| | | |

Baci_halodurans_NP_241368 VRFSWAYPLYKNML-ANFWTPFEINMSH----DAKQFPT---------LTETEQEAFKKIIGLLAFLD-----------------------------------------SVQTDYSMRAAEYLTD-------------------SSLAALMSVLSFQEVVHN--QSYSYVLSSLVPK----------ATQD------EI------FEYWKHDDVLKERN----EFIIDGYEKFVDN-----------------------

Pae_sp_ZP_04851883 IRMPHMYKLYKVLL-LNHWIADEIPMAK----DAQQFAL---------LDPEEQRTFKINISLLAVLD----------------------------------------------SMQTMFVGDVKR--------------YFTDSSLEAISAIIGQQEVVHN--QSYSYVLSSIVSE---------QEQKE-------------IFEYWKHDPVLLDRNKFI-ANIYQNFRDEP-------------------------

Bact_vulgatus_YP_0130035 MRYKWVSDWYRQAM-NNFWIPEEINLTQ----DTKDYPH---------LDQAERTAYDKILSFLVFLD-----------------------------------------SLQSNNLPTISEYITAN-------------------EVNLCLHIQAFQECVHS--QSYSYMLDSICSP---------EKRNE------------ILYQWKTDGHLLKRNTFIG-NCYNEFQESQD-------------------------

Clo_botulinum_YP_002805314 IKYSWASDFYRTML-NNFWIPEEISLNE----DIKQFPY---------LTDGERNAFDKIISFLNFLD-----------------------------------------SVQSENLPNISRYIT-------------------AAEVSSLLNIQTFQEEIHA--QSYSYILDTVTNP----------ITRD------------KIYDQWREDEHLLERNKFI-AGIYEKFNKEP-------------------------

Cya_sp_ATCC_51142_YP_001803056 VRYTWAVGLYQQMR-ENFWIPQRLDVTQ----DVTDYAN---------LTDDERYAYDGILSYLTFLD-----------------------------------------SVQTCNIPHLKSSITA-------------------PEISLCMAEQISQEGMHN--QSYQYLIETIIPP-----------DRR-----------TQVYDFWRTDKVLKDRCEFIAKLYQKYIDDAT-------------------------

Cya_sp_CCY0110_ZP_01726237 VRYAWAVGLYQQMR-ENFWIPQRLDVTQ----DVTDYAN---------LTDDERYAYDGILSYLTFLD-----------------------------------------SVQTCNIPHLKSSIT-------------------APEISLCMAEQISQEGMHN--QSYQYLIETIIPP-----------ERR-----------TQVYEFWRTDTVLKDRCEFIAKLYQKYIDDPT-------------------------

Cya_sp_ATCC_51142_YP_001806290 TRYGWARTIYQTMR-EGYWIPQRTDLSQ----DKLDYVN---------LIPGERRALKGILSYLNFLD-----------------------------------------SVQVANIPELSRYITA-------------------PEARMCLAEQTSQEAMHG--ETYQYITESIIPE----------NEQH------------EVYDFWKKDLLLNERCEFIASYYQTLADNPCG------------------------

Cya_sp_CCY0110_ZP_01729893 TRYGWARSIYQTMR-ESFWVPQRTDLSQ----DKLDYAN---------LIPGERRALKGILSYLNFLD-----------------------------------------SVQVANIPELSRYITA-------------------PEARMCLAEQTSQEAMHG--ETYQYITESIIPE----------NEQH------------EVYDFWKQDLLLHERCEFIAGYYQNLANNPTG------------------------

Cau_cresents_NP_419079 FRYPWAYDFWKKQQ-QVHWMPEEVPLGE----DLKDWAVK--------LNDKERNLLTQIFRFFTQSD-----------------------------------------VEVQDNYMERYGRVFKP------------------TEVKMMLASFANMETIHI--AAYALLLETIGMP-----------ETE--------------FSAFMEYEAMKAKH----DYMQTFGVDSN-------------------------

Cau_sp_YP_001686327 FRYPWAHEFWKKQQ-QVHWMPEEVPLGE----DLKDWAVK--------LNDKERNLLTQIFRFFTQSD-----------------------------------------IEVADNYMERYGRVFKP------------------TEVKMMLSSFANMETIHI--AAYALLLETIGMP-----------ESE--------------FGAFMQYQAMRDKH----DFMQKFGVETN-------------------------

Neo_sennetsu_YP_506404 FQYDWAYQAWEIQQ-KIHWLPEEIPMAD----DVQDWHHK--------ICGTEKNLLTQIFRFFTQAD-----------------------------------------VEVHDCYMRHYAGVFK------------------PPEVCMMLTAFANMETIHI--AAYAHLLDTVGMP-----------ETE--------------YLAFTKYKQMKEKC----EYLKAFKMDNP-------------------------

Ori_tsutsugamushi_YP_001248105 FSYPWAYKAWHTQQ-KIHWLPEEVPLAD----DIKDWKYN--------LTPGEKHLLTQIFRFFTQAD-----------------------------------------IEVNNCYMKHYARVFQP------------------TEVQMMLSAFSNMETIHI--AAYSHLLDTIGMP-----------ETE--------------YQAFMKYKAMKDKY----DYMQRFNVDNK-------------------------

Ric_rickettsii_YP_001494766 FSYPWAYEAWHTQQ-KIHWLPEEVPLAD----DVKDWKYN--------LTPGEKHLLTQIFRFFTQAD-----------------------------------------IEVNNCYMKHYSRVFKP------------------TEILMMLSAFSNMETVHI--AAYSHLLDTVGMP-----------EVE--------------YSAFLKYKEMKDKY----DYMQQFGVDAK-------------------------

Wol_pipientis_YP_001974856 FNYPWAYDAWLQQQ-RIHWIPEEVPLAD----DVKDWKTK--------LSNVEKNLLTQIFRFFTQAD-----------------------------------------IEVNNCYMRHYSNIFKP------------------TEICMMLASFSNMETIHI--AAYSYLLDTIGMP-----------ESE--------------YQAFLKYDAMRKKYEYMLEFEESKKQDKK-------------------------

Wol_sp_NP_966023 FNYPWAYDAWLQQQ-RIHWIPEEVPLAD----DVKDWKTK--------LSSVEKNLLTQIFRFFTQAD-----------------------------------------IEVNNCYMRHYSNIFKP------------------TEICMMLASFSNMETIHI--AAYSYLLDTIGMP-----------ESE--------------YQAFLKYDAMRKKY----EYMLEFEESKK-------------------------

Bau_cicadellinicola_YP_588829 QKYESFEKLIEKQL-SFFWRPEEVDISL----DRIDYQA---------LPQHEKHIFISNIKYQTLLD-----------------------------------------SIQGRSPNVALLPLIS------------------IPELETWVETWAFSETIHS--RSYTHIIRNIVND------------PS------LV------FDDIVTNTEILKRA----KDIAAFYDDLIQMTSYFHLFG-EGNHSINGKLVIVN

Esc_coli_YP_001731173 QKYDIFEKLIEKQL-SFFWRPEEVDVSR----DRIDYQA---------LPEHEKHIFISNLKYQTLLD-----------------------------------------SIQGRSPNVALLPLIS------------------IPELETWVETWAFSETIHS--RSYTHIIRNIVND------------PS------VV------FDDIVTNEQIQKRA----EGISSYYDELI-EMTSYWHLLGEGTHTVNGKTVTVS

Shi_dysenteriae_YP_403993 QKYDIFEKLIEKQL-SFFWRPEEVDVSR----DRIDYQA---------LPEHEKHIFISNLKYQTLLD-----------------------------------------SIQGRSPNVALLPLIS------------------IPELETWVETWAFSETIHS--RSYTHIIRNIVND------------PS------VV------FDDIVTNEQIQKRA----EGISSYYDELI-EMTSYWHLLGEGTHTVNGKTVTVS

Sod_glossinidius_YP_455265 QKYDIFEKLIEKQL-SFFWRPEEVDVSR----DRIDYQA---------LPEHEKHIFISNLKYQTLLD-----------------------------------------SIQGRSPNVALLPLIS------------------IPELETWVETWAFSETIHS--RSYTHIIRNIVND------------PS------LV------FDDIVTNEEILKRA----KDISGYYDGLIELTSYYHLLG-EGTHQVNGKTVVVN

Yer_pestis_A_ZP_04509308 QKHAIFEKLIEKQL-SFFWRPEEIDVSR----DRIDYNA---------LPDHEKHIFISNLKYQTLLD-----------------------------------------SIQGRSPNVALLPLIS------------------IPELETWVETWSFSETIHS--RSYTHIIRNIVND------------PS------VV------FDDIVTNEEILKRA----KDISAYYDDLIEMTSYYHLLG-EGTHQVNGKTVVVK

Buc_aphidicola_NP_240009 QKYKIFEQLIEKQL-SFFWRPEEIDLSR----DRIDFQN---------LPDNEKHIFISNLKYQTLLD-----------------------------------------SIQGRSPNIAFLPIIS------------------IPELETWIETWSFSETIHS--RSYTHIIRNIVNC------------PS------LV------FDDIISNKNIYDRA----QNISIYYDELINLTSYWHLLG-EGIHLINGKKIHIN

Yer_pestis_A_ZP_04512133 QKYRDFEKLIEKQL-SFFWRPEEVDITT----DRIDFNT--------KLQEHERHIFLSNLRYQTLLD-----------------------------------------SVQGRSPNATLLPLIS------------------IPELETWVETWSFSETIHS--RSYTHIIRGMVDDPSIVFDGIVTDEEIIS----RAVSISSEYDRLYEMTCARQHLGED-EFERLYVSEFD-----------------------GK

Halom_mukohataei_ZP_03875489 YEYPDVLEYKDAIR-NSYWVHTEFNFSG----DVQDFKVN--------TTPAEKAVIKRTMLAIAQIE------------------------------------------VQVKTFWADIYDEMP------------------KAEVGNVGMTFAESEVRHM--DAYSHLLDILGIT------------ED--------------FEEVTDVPAIEERIDYLDEYLEKSESDDT-------------------------

Natro_pharaonis_YP_327710 YEYADFLDYKDAIR-NSYWVHTEFNFSG----DVQDFRTN--------TTPAEKTVIKRTMLAIAQIE------------------------------------------VQVKTFWADIYDEMP------------------KTEVGNVGMTFAESEVRHM--DAYSHLLDILGIT------------ED--------------FEEVTDVPAIEDRIEYLDKYLEKSESDDK-------------------------

Halor_lacusprofundi_YP_002564382 YEYNDFLDYKDAIR-NSYWVHTEFNFSG----DVQDFKVN--------TTPAEKTVIKRTMLAIAQIE------------------------------------------VQVKTFWSDIYEEMP------------------KAEIGSVGMTFAESEVRHM--DAYSHLLDVLGITG--------------------------DFEEVTEVPAVKDRIEYLDECLERGQSDDT-------------------------

.* . . * :: *: : : . * * :* : : :

Högbom 2010 positions 1 * 2 | 3 45 6 7 891011 12 13

Högbom 2010 exceptions | * | | | | *| | * |

Chl_muridarum_NP_296594 IKYKWAWEHYLNGC-ANNWLPTEISMGK----DIELWKSN-------VLSEDERRVILLNLGFFSTAE-----------------------------------------SLVGNNIVLAIFKHVT------------------NPEARQYLLRQAFEEAVHT--HTFLYICESLGLD------------EK------EI------FNAYNERASIKAKD----DFQMEITGKVLDPNFRTDS-----------------

Chl_trachomatis_YP_328659 IKYKWAWEHYLNGC-ANNWLPTEIPMGK----DIELWKSD-------RLSEDERRVILLNLGFFSTAE-----------------------------------------SLVGNNIVLAIFKHVT------------------NPEARQYLLRQAFEEAVHT--HTFLYICESLGLD------------EK------EI------FNAYNERAAIKAKD----DFQMEITGKVLDPNFRTDS-----------------

Halob_sp_NP_280997 IDYDWAREYYQQGV-ANNWTPEEVAMQG----DISQWQGN-------ELTDAERQLVEWNLGFFSTAE-----------------------------------------SLTANNIVLAIYEYVT------------------APECRQYLLRQAYEEAVHT--DTFIYCCDSLGFDP------------------------EHMYGMYDRIPSIAEKDEFV-VGLTRAVDDQAFTIDTDDDLR---------------

Halog_borinquense_ZP_04000565 IDYDWARQYYRDGV-ANNWVPEEIPMQE----DIAQWNG-D------ALSDSEKQLVEWNLGFFSTAE-----------------------------------------SLTANNIVLALYEYVT------------------APECRQYLLRQAYEEAIHT--DTFIYCCDSLGFDP------------------------EYMYGMYDRVPSIKEKDEFV-VDLTRNLDDPD---------------------FTVE

Halom_utahensis_YP_003131236 IDYDWAREYYEAGV-NNNWVPEEIPMQD----DVAQWNGD-------ELTDAERQLVEWNLGFFSTAE-----------------------------------------SLTANNLVLALYEYVT------------------APECRQYLLRQAYEEAIHT--DTFIYCCDSLGFEP------------------------EYLYGMYDRVPSIEEKDQFV-VDLTRSIDDPEFTIDGDSD-----------------

Natri_magadii_ZP_03692956 IDYDWAREYYEAGV-NNNWVPEEIPMQD----DVSQWNG-D------ALSDAERQLVEWNLGFFSTAE-----------------------------------------SLTANNIVLAVYDHVT------------------APECRQYLLRQAYEEAIHT--DTFIYCCDSLGFEP------------------------DYMYGMYDRIPSIAEKDEFV-IDLTRVINQNG---------------------FVIE

*.*.** ::* * *** * *:.* *: *:. *:: *:::: ********* **..**:***::.:** **.********:***:** .**:* *:***:: :. *:. .:* **

Högbom 2010 positions 1 * 2 | 3 45 6 7 891011 12 13

Högbom 2010 exceptions | * * | | *| * *| *| | |

Geo_kaustophilus_YP_148624 -----MFKLYEKAKRNGKWNPADIDFSQ----DQKDFAS---------LTSEEKISALPLVAGFSAGE-----------------------------------------EAVTLDILPMAHALARQG----------------RLEDVLFLTTFMHDEAKHV--EMFSRWQQAVGIG---------QMDLS--------------VFHNDHYKRIFYEALPE-AMNRLYADDSP-------------------------

Geo_sp_ZP_03557705 -----MYKLYEKAKRNGKWNPADIDFSQ----DKEDFAR---------LTSEEKIAALPLVAGFSAGE-----------------------------------------EAVTLDILPMAHALARQG----------------RLEDVMFLTTFMHDEAKHV--EMFSRWQQEVGIG---------AMDLS--------------VFHNDHYKRIFYEALPE-AMNRLYTDDSP-------------------------

Myc_avium_NP_962606 ----LPLKLFAGGN-AKFWNPADIDFSR----DRADWER---------LTDDERSYATRLCAEFIAGE-----------------------------------------ESVTQDIQP-FMAAMRA---------------EGRLGDEMYLTQFAFEEAKHV--QVFRMWLDAVGVT-------------D------DL------HHFLDDVPSYRTIFY---EELPDCLNALTIDPS---------------------

Myc_bovis_NP_853903 ----LPLKLFAGGN-AKFWDPADIDFTR----DRADWEK---------LSDDERDYATRLCTQFIAGE-----------------------------------------EAVTEDIQP-FMSAMRA---------------EGRLADEMYLTQFAFEEAKHT--QVFRMWLDAVGIS-------------E------DL------HRYLDDLPAYRQIFY---AELPECLNALSADPS---------------------

Myc_tuberculosis_NP_214747 ----LPLKLFAGGN-AKFWHPADIDFTR----DRADWEK---------LSDDERDYATRLCTQFIAGE-----------------------------------------EAVTEDIQP-FMSAMRA---------------EGRLADEMYLTQFAFEEAKHT--QVFRMWLDAVGIS-------------E------DL------HRYLDDLPAYRQIFY---AELPECLNALSADPS---------------------

Nat_pharaonis_YP_331256 ------MRYYRNAV-ERHWDPHDIELDT----DRENLKAFLDS---TDTPETYLDGLRSGVARFGAGE-----------------------------------------QAVTEDLAPLATALGS-------------------IDDQMFVTTQIYEEAKHT--DFFDRYWREVIHP---------------------------VEDALGLDPSSPTDETWFNDAYEELFDRNE------------------EAMERLL

Nat_pharaonis_YP_330945 ----GYFKHAVYN----HWDPYEDIEKALIEQDRKRLIDAD------EFGETEFEDLMRTLALFGAGE-----------------------------------------EAVTEDLAPLMLVVDD-------------------INDQMFVSSQIYEEAKHT--QFFDRYWREVIHP--------VADARG-----FERRAPTDQRFFVDGYVDLFDRT---EAAMERLLEPGE-------------------------

Sul_islandicus_YP_002913609 ------MKLYQLGK-KLFWDPANIDLSK----DAEDWKK---------LNDLEKMFIINVGSKFAAGE-----------------------------------------EAVALDLHPLIVTLVKE----------------GRVEEVMYLEQFVYEESKHV--EAFRRFFDAVNVMEDLS-----AYTKD---------------LSPNYKKIFYEELPKA-MWNLSRDPSPE-------------------------

Sul_solfataricus_NP_343843 ------MKLYQLGK-KLFWDPANIDLSK----DAEDWKK---------LNDLEKMFIINVGSKFAAGE-----------------------------------------EAVALDLHPLIVTLVKE----------------GRVEEVMYLEQFVYEESKHV--EAFRRFFDIVNVMEDLS-----AYTKD---------------LSPNYKKIFYEELPKA-MWNLSRDPSPE-------------------------

: *.* : * . : * *** ::*: *: * : : ::: .:*:**. : * : *

1JK0:A S. cerevisiae Y2 αE αF αG αH

.........--------------..........-......... .......................... .................... .---------.................

HHHHHHHHH--------------HHHHTTTHHH-HHHHHHHHHTT HHHHHHHHHHHHHHHHHHHHHHHHHHT SS HHHHHHHHHHHHHHHHHHHHTTS GGGGT H---------HHHHHHHHHHHHHHHHTTT SS

**1**SMQ:A S. cerevisiae HHHHHHHHH--------------HHHHTTTHHH-HHHHHHHHHTT HHHHHHHHHHHHHHHHHHHHHHHHHHH SS HHHHHHHHHHHHHHHHHHHTTT GGGGT H----------HHHHHHHHHHHHHHHHHHT SS

1JK0:B S. cerevisiae Y4 HHHHHHHHH--------------HHHHTTTHHH-HHHHHHHTSSS-----------S HHHHHHHHHHHHHHHHHHHHHHHHHHT SS HHHHHHHHHHHHHHHHHHHTTS THHHHHHHHHHHHTT

1SMS:A S. cerevisiae HHHHHHHHH--------------HHHHTTTHHH-HHHHHHHHTTT-----------S HHHHHHHHHHHHHHHHHHHHHHHHHTT SS HHHHHHHHHHHHHHHHHHHHTTS STTTTS T----------HHHHHHHHHHHHHHHHTTS SS

3HF1:A H. sapiens HHHHHHHHH--------------HHHHTTTHHH-HHHHHHHHHTT HHHHHHHHHHHHHHHHHHHHHHHHHHT SS HHHHHHHHHHHHHHHHHHHHTTS GGGGT H----------HHHHHHHHHHHHHHHHHTT SS

2UW2:A H. sapiens -----S HHHHHHHHH--------------HHHHTTTHHH-HHHHHHHHTSS HHHHHHHHHHHHHHHHHHHHHHHHHHT SS HHHHHHHHHHHHHHHHHHHHTTS GGGGT H----------HHHHHHHHHHHHHHHHHTT SS

2VUX:A H. sapiens HHHHHHHHH--------------HHHHTTTHHH-HHHHHHHHHTT HHHHHHHHHHHHHHHHHHHHHHHHHTT SS HHHHHHHHHHHHHHHHHHHHTTS GGGGT H----------HHHHHHHHHHHHHHHHHTT SS

1H0N:A M. musculus -----S HHHHHHHHH--------------HHHHHHHHHH-HHHHHHHHHTT HHHHHHHHHHHHHHHHHHHHHHHHHTT SS HHHHHHHHHHHHHHHHHHHHTTS GGGGT H----------HHHHHHHHHHHHHHHHHTT SS

2O1Z:A P. vivax HHHHHHHHH--------------HHHHTTTHHH-HHHHHHHHHTT HHHHHHHHHHHHHHHHHHHHHHHHHHT SS HHHHHHHHHHHHHHHHTTBTTTB TTTT H----------HHHHHHHHHHHHHHHHHTT SS

2P1I:A P. yoelii HHHHHHHHH--------------HHTTTTTHHH-HHHHHHHHTTT STTHHHHHHHHHHHHHHHHHHHHHHHHT SS HHHHHHHHHHHHHHHHHHHTTTS STTTT H----------HHHHHHHHHHHHHHHHHTT SS

2RCC:A B. halodurans HHHHHHHHHHHH--------------HHHHTTTHHH-HHHHHHHHHTT-----------SSHHHHHHHHHHHHHHHHHHHHHHHHHHHHHHH G-----GG SHHHHHHHHHHHHHHHHHHHHHHHHHTT----T STT H----------HHHHHHHHHHHHHHHHHTT S

2ALX:A E. coli HHHHHHHHHHHHHHHH--------------HHHHTHHHHH-HHHHHHHHHTT HHHHHHHHHHHHHHHHHHHHHHHHHHHHTTTSS HHHHHHHHH-THHHHHHHHHHHHHHHHHHHHHHTS---S BTTB H----------HHHHHHHHHHHHHHHHTTS SS

1AV8:A E. coli HHHHHHHHHHHHHHHH--------------HHHHTHHHHH-HHHHHHHHHTT SHHHHHHHHHHHHHHHHHHHHHHHHHHHHHHSSS HHHHHHHHH-THHHHHHHHHHHHHHHHHHHHHHHT---T BTTB H----------HHHHHHHHHHHHHHHHHTT S

1JPR:A E. coli HHHHHHHHHHHHHHHH--------------HHHHTHHHHHHHHHHHHHTTT HHHHHHHHHHHHHHHHHHHHHHHHHHHHHHTSS HHHHHHHHH-THHHHHHHHHHHHHHHHHHHHHHTTT BTTBSH----------HHHHHHHHHHHHHHHHHTT S

1JQC:A E. coli HHHHHHHHHHHHHHHH--------------HHHHTHHHHH-HHHHHHHHHTT HHHHHHHHHHHHHHHHHHHHHHHHHHHHHHTSS HHHHHHHHH-THHHHHHHHHHHHHHHHHHHHHHTT---T BTTBSH----------HHHHHHHHHHHHHHHHHTT S

1MXR:A E. coli HHHHHHHHHHHHHHHH--------------HHHHTHHHHH-HHHHHHHHHTT HHHHHHHHHHHHHHHHHHHHHHHHHHHHHHTSS HHHHHHHHH-THHHHHHHHHHHHHHHHHHHHHHTT---T BTTBSH----------HHHHHHHHHHHHHHHHHTT S

1SYY:A C. trachomatis HHHHHHHHHHHHHHH-------------TTTTTTHHHHH-HHHHHHHHHTT-----------SSHHHHHHHHHHHHHHHHHHHHHHHHHHHHHHH GGG HHHHHHHHHHHHHHHHHHHHHHHHH S----S BTTB H----------HHHHHHHHHHHHHHHHHTT SS

2ANI:A C. trachomatis HHHHHHHHHHHHHHH-------------TTIIIIITHHH-HHHHHHHHHTT-----------SSHHHHHHHHHHHHHHHHHHHHHHHHHHHHHHH GGG HHHHHHHHHHHHHHHHHHHHHHHHH S----S STT H----------HHHHHHHHHHHHHHHHHTT SS

3EE4:A M. tuberculosis HHHHHHHHHHHI-------------IIIITTHHHHH-HHHHHHHHTTTT HHHHHHHHHHHHHHHHHHHHHHHHHHHHHHH THH--HHHHHHHHHHHHHHHHHHHHHHHHTTTT STT H----------HHHHHHHHHHHHHHHHHHHTTTT

S. cerevisiae S288c NP 012508 (Y2) 230 240 250 260 270 280 290 300 310 320 330 340

coordinates . . . . . . . . . . . .

matrix position 640 650 660 670 680 690 700 710 720 730 740 750 760 770 780 790 800 810

. . . . . . . . . . . . . . . . . .

E. coli electron transfer pathway ⧫

C. trachomatis electron transfer pathway ❖

S. typhimurium electron transfer pathway ◊

diiron center residues ⚫ ⚫ ⚫

hydrophobic residues ⯀ ⯀ ⯀

conserved residues (Wang 1997) ↓ ↓ ↓ ↓ ↓ ↓↓↓ ↓

Högbom 2010 positions 14 15 16 17 18 19 20 21 22 23 24 25 | 26 27 | | |28

Högbom 2010 exceptions |* *| * *|| | * | | | | | |*

Bab_bovis_XP_001610573 ------CFYIKVILQC---------------ISKAIFRSA-FGILADYLRNSK----------KLPTLLSALDTVAKDVSIHLKFAMAALEHLKLR----------PT--RETVMELLNSAYDLEMAYCRSVLP----MDHMGLSEY----------HLSQYIKNNVNQCLLITGNTEEH

Bab_equi_6m007985 ------SFFKRVLLML---------------ISKLVFSAS-FGIIRDFIVKEK----------IIPTYALALDAIESDQHIHARFAFCLFELMEN------------RMLESEVLSLINEVKELEIAFCLSSLP----LEFMKLQK----------DHITSYVESRLNQVLLVSGYKAIF

The_annulata_XP_953574 ------CFFKRVLLLV---------------IMKLLFYCS-INILRDYLLKEK----------LLPTFITAFDSITNDRIIHTRFAHCTFRLLQN------------KMLESEVLEIIKQALKLEYQFILQYFT----IQFFNLST----------NVIKSYLEWIGNNILLVCGYKTLY

The_parva_XP_766717 ------CFFKRVLLVV---------------ISKLLFTAT-FNILRDYLVKEK----------LLPTFTLALDAVSKDRHIHTRFAHCTFTLLQH------------KMLESDVVQLIKEALKLEYDFANKFLP----LSLMAITQMLT-------DVLRGYMEWVGNNVLMVCGYRTLY

Pla_berghei_Pdb_121420 -------FYNKLILLY---------------ISKVLFNGG-FSILASHCKENN----------ILSCFTGVHEKIQRDEYLQGDFSVMCCNHLNN----------KLK--YESVLYYFKSAVELEYNFCVDMLD----FNFLKITK----------EQVKQFLQYLADTMLLNLKYPKHF

Pla_yoelii_XP_727957 -------FYNKLILLY---------------ISKVLFNGG-FSILASYCKENN----------ILSSFTGVHAKIQRDEYLQGDFSVMCCNHLNN----------KLR--YENVLNYFKAAVELEYNFCVDMLD----FDFLKITK----------DQVKQFLQYLADTMLLNLKYPKHY

Pla_chabaudi_PCAS_121490 -------FYNKLILLY---------------ISKVLFNGG-LSIVSSYCKENG----------ILPCLTSVYDKIQRDEYLQGDFSVMCCNHLNN----------KLK--YENVLDYFKTAVDLEYNFCVEMLD----FDSLKITK----------DQVKQFLQYLADTMLVNLKYSKHY

Pla_falciparum_XP_001347439 -------YYNKLVLLY---------------ISKVLFNGT-LNILIGYCKENS----------ILPCLCNVHEKIHRDEYLHGDFSVMCCNHLVN----------KLK--YEHVLDYFKMAVDLEYQFSLEMLE----LNVLKIKK----------EQVRAFLEYLTDTMLTNLKYPKHY

Pla_knowlesi_XP_002258799 -------FYNKLVLIY---------------ISKVLFNGT-LNILIGYCKENS----------ILPCLCNVHEKIHRDEYLHGDFSVMCCNHLNN----------KLK--YERLLDYFKMAVKLEYQFAIEMLD----LSVLKIKK----------EQVKSFLEYLADTMLVNLKHPKYF

Pla_vivax_XP_001614470 -------FYNKLVLIY---------------ISKVLFNGT-LNILIGYCKENS----------ILPCLCNVHEKIHRDEYLHGDFSVMCCNHLNN----------KLK--YESLLDYFKMAVELEYQFSMEMLE----LSVLKIKK----------EQVKSFLEYLADTMLVNLKHPKYF

Cry_hominis_XP_665685 ------LFSEKLIYYV---------------IIKKIFSSSIHLLRLSFSMRNISESE------LPTQLCDGLDRIRRDEQYQVEFCLSIAESMKT----------KPS--NDQVVNLLEQAIMLEKNFIFSIVDP----QVLQIER----------EDIVAFLLFNADEILVDLKYPRQF

Cry_parvum_XP_001388304 ------LFSEKLIYYV---------------IIKKIFSSSIHLLRLSFSMRNISESE------LPTQLCDGLDRIRRDEQYQVEFCLSIAKSMKT----------KPS--NDQVVNLLEQAIMLEKNFIFSIVDP----QVLQIER----------EDIVAFLLFNADEILVDLKYPRQF

Cry_muris_XP_002140092 -------FSEKLIIYT---------------IIKRVFSSGIHLIRQSFFNKEGP---------VLEQLCDALDRIRRDELFQAEFCLSIARSMKT----------KPS--NEYIISMLENAITIEKGFIFSIFN----LEILEIPR----------EDIVAYLLFSADEILVELKYPRHF

: ::: * * :* : .. .. : * : *. : . :: :: . :* : . . . : : : :: : * .

Högbom 2010 positions 14 15 16 17 18 19 20 21 22 23 24 25 | 26 27 | | |28

Högbom 2010 exceptions | |* ** * **| || * | | |found | | |*

Ano_gambiae_XP_308927 -----ANFGERVVAFA--------------AVEGIFFSGS-FAAIFWLKKRG-----------LMPGLTFSNELISRDEGLHCDFACLMFKYLVQ----------KPS--KERVTEIIRDAVLIEQEFLTKALP----VDMLGMNC----------DLMSQYIEFVADRLLLELGCEKIY

Dro_melanogaster_NP_525111 -----ANFGERIIAFA--------------AVEGIFFSGS-FASIFWLKKRG-----------LMPGLTFSNELISRDEGLHCDFAVLMFQHLVQ----------RPK--RERIIEIIRDAVAIEQEFLTDALP----VNLIGMNC----------DLMSQYIEFVADRLLVELGVGKIY

Dan_rerio_NP_571525 -----ARYGERVVAFA--------------AVEGIFFSGS-FASIFWLKKRG-----------LMPGLTFSNELISRDEGLHCDFACLMFKHLIN----------KPS--EETVKKIIMNAVEIEQEFLTDALP----VKLIGMNC----------DLMKQYIEFVADRLLLELGFDKVY

Gal_gallus_XP_001231545 -----ATYGERVVAFA--------------AVEGIFFSGS-FASIFWLKKRG-----------LMPGLTFSNELISRDEGLHCDFACLMFKHLIR----------KPS--EERVKEIIMNAVLIEQEFLTEALP----VKLIGMNC----------TLMKQYIEFVADRLMLELGFRKIY

Hom_sapiens_isoform_2_NP_001025 -----ATYGERVVAFA--------------AVEGIFFSGS-FASIFWLKKRG-----------LMPGLTFSNELISRDEGLHCDFACLMFKHLVH----------KPS--EERVREIIINAVRIEQEFLTEALP----VKLIGMNC----------TLMKQYIEFVADRLMLELGFSKVF

Mus_musculus_NP_033130 -----ATYGERVVAFA--------------AVEGIFFSGS-FASIFWLKKRG-----------LMPGLTFSNELISRDEGLHCDFACLMFKHLVH----------KPA--EQRVREIITNAVRIEQEFLTEALP----VKLIGMNC----------TLMKQYIEFVADRLMLELGFNKIF

Rat_norvegicus_NP_001020911 -----ATYGERVVAFA--------------AVEGIFFSGS-FASIFWLKKRG-----------LMPGLTFSNELISRDEGLHCDFACLMFKHLVH----------KPS--EQRVKEIITNSVRIEQEFLTEALP----VKLIGMNC----------TLMKQYIEFVADRLMLELGFNKIF

Xen_laevis_NP_001079369 -----ATFGERVVAFA--------------AVEGIFFSGS-FASIFWLKKRG-----------LMPGLTFSNELISRDEGLHCDFACLMFKHLIR----------KPS--EERVVELITDAVQIEQEFLTEALP----VNLIGMNC----------TLMKQYIEFVADRLLLELGFKKVF

Xen_Silurana_tropicalis_NP_001007890 -----ATYGERVVAFA--------------AVEGIFFSGS-FASIFWLKKRG-----------LMPGLTFSNELISRDEGLHCDFACLMFKHLVN----------KPS--EERVVQLITDAVQIEQEFLTEALP----VNLIGMNC----------TLMKQYIEFVADRLLLELGFNKVF

Xen_laevis_NP_001080772 -----ATYGERVVAFA--------------AVEGIFFSGS-FASIFWLKKRG-----------LMPGLTFSNELISRDEGLHCDFACLMFRHLVH----------KPS--EERVVQLITNAVQIEQEFLTEALP----VNLIGMNC----------TLMKQYIEFVADRLLLELGFNKVF

Xen_laevis_NP_001085389 -----APYCERLVAFA--------------AVEGIFFSGS-FASIFWLKKRG-----------LMPGLTFSNELISRDEGLHTDFACLLFKHVVH----------KPS--EQRVREIIIDAVKIEQEFLTEALP----VELIGMNC----------TLMKQYIEFVADRLLLELGFKKIF

Xen_Silurana_tropicalis_NP_989048 -----APFCERIVAFA--------------AVEGIFFSGS-FASIFWLKKRG-----------LMPGLTFSNELISRDEGLHTDFACLLFKHVVH----------KPS--EQRVREIIIDAVKIEQEFLTEALP----VELIGMNC----------KLMKQYIEFVADRLLLELGFKKIF

Gal_gallus_XP_418364 -----STFGERVVAFA--------------AVEGIFFSGS-FAAIFWLKKRG-----------LMPGLTFSNELISRDEGLHCDFACLMFHYLVN----------RPS--EERVREIIVNAVEIEQEFLTEALP----VGLIGMNC----------TLMKQYIEFVADRLLMELGFSKVF

Hom_sapiens_isoform_1_NP_056528 -----STFGERVVAFA--------------AVEGVFFSGS-FAAIFWLKKRG-----------LMPGLTFSNELISRDEGLHCDFACLMFQYLVN----------KPS--EERVREIIVDAVKIEQEFLTEALP----VGLIGMNC----------ILMKQYIEFVADRLLVELGFSKVF

Mus_musculus_NP_955770 -----STFGERVVAFA--------------AVEGIFFSGS-FAAIFWLKKRG-----------LMPGLTFSNELISRDEGLHCDFACLMFQYLVN----------KPS--EDRVREIIADAVQIEQEFLTEALP----VGLIGMNC----------VLMKQYIEFVADRLLGELGFSKIF

Rat_norvegicus_NP_001124015 -----STFGERVVAFA--------------AVEGIFFSGS-FAAIFWLKKRG-----------LMPGLTFSNELISRDEGLHCDFACLMFQYLVN----------KPS--EERVMEIIVDAVKIEQEFLTEALP----VGLIGMNC----------VLMKQYIEFVADRLLGELGFSKIF

Xen_Silurana_tropicalis_NP_001119973 -----ASFGERVVAFA--------------AVEGIFFSGS-FAAIFWLKKRG-----------LMPGLTFSNELISRDEGLHTDFACLMFHYLVK----------KPT--EERVTEIIVNAVRIEQEFLTEALP----VSLIGMNC----------TLMKTYIEFVADRLLVELGCSKAF

Dan_rerio_NP_001007164 -----STFGERLVAFA--------------AVEGIFFSGS-FAAIYWLKKRG-----------LMPGLTYSNELISRDEGLHCNFACLIYSYLVK----------KPS--VDRVNDIIAKAVSIEQEFLTEALP----VNLIGMNC----------SLMKQYIEFVADRLLTDLGLPKAY

Dap_pulex_GNO_1472053 -----APYSERIVAFA--------------AVEGIFFSGS-FAAIFWLKKRG-----------LMPGLTFSNELISRDEGLHTDFACVMFKHLVN----------KPP--KERIIEIINNAVKIEQEFLTDALP----VEMIGMNC----------RLMRQYIEFVADRLLLELGCTKLY

Asp_clavatus_XP_001274524 -----SSFGQRLVAFA--------------AVEGIFFSGS-FASIFWLKKRG-----------LMPGLTFSNELISRDEGLHTDFACLLFSHLNN----------RPD--KKVVEDIIVEAVAIEQEFLTDALP----VALLGMNS----------KLMCQYIEFVADRLLVALGNKKYY

Neu_crassa_XP_962820 -----STFAQRLVAFA--------------AVEGIFFSGA-FASIFWLKKRG-----------LMPGLTFSNELISRDEGLHTDFACLLFSHLNN----------RPS--KQLIQEIIVDAVRIEQEFLTEALP----CALLGMNA----------DLMKQYIEFVADRLLVALGNEKIY

Dap_pulex_GNO_1331594 -------FAERLIAFA--------------AVEGIFFSGS-FCSIFWLKKRG-----------LMPGLTFSNELISRDEGVHCDFAVHLHNHHLIN---------KVP--KERIREILVDALNIEREFITESLP----VSLIGMNAG----------LMTQYLEFVTDRLLVELGCEREY

Can_albicans_XP_715277 -----ALYGERLVAFA--------------AVEGIFFSGS-FASIFWLKKRG-----------LMPGLTFSNELICRDEGLHTDFACLLFAHLQN----------RPS--PEIVERIITEAVDVEKEYFTDALP----VSLLGMNC----------NLMCQYIEFVADRLLLALGNKKVY

Can_albicans_XP_713125 -----ALYAERLVAFA--------------AVEGIFFSGS-FAAIFWLKKRG-----------LMPGLTFSNELICRDEGIHTDYACLLFSYLKN----------KPS--SEIIEKIITEAVDIEKEYFSDALP----VSLLGMNC----------DLMCQYVEFVADRLLVAFGNKKYY

Sac_cerevisiae_S288c_NP_012508 -----ALFGERLVAFA--------------SIEGVFFSGS-FASIFWLKKRG-----------MMPGLTFSNELICRDEGLHTDFACLLFAHLKN----------KPD--PAIVEKIVTEAVEIEQRYFLDALP----VALLGMNA----------DLMNQYVEFVADRLLVAFGNKKYY

Lei_braziliensis_XP_001565976 ------SFQERLIGFA--------------AVEGIFFSGS-FCALFWLKKRG-----------LMPGLTFSNELISRDEGLHTDFACLLYNSHIKH---------KLP--RERVLEIIVDAVNIEREFICDALP----VRLIGMNA----------ELMAQYIEFVADRLLVSLGEEKHY

Lei_braziliensis_XP_001565036 ------SFQERLIGFA--------------AVEGIFFSGS-FCALFWLKKRG-----------LMPGLTFSNELISRDEGLHTDFACLLYNSHIKH---------KLP--RERVLEIIVDAVNIEREFICDALP----VRLIGMNA----------ELMAQYIEFVADRLLVSLGEEKHY

Try_cruzi_XP_813233 ------SFAERLVAFA--------------AVEGIFFSGS-FCALFWLKKRG-----------LMPGLTFSNELISRDEGLHTDFACLLYENYIVN---------KLP--KERIREIICSAVDIEREFICDAIP----VRLLGMNA----------DLMSTYIEFVADRLLVALGNERHY

Par_tetraurelia_XP_001454302 ------SFAERLIAFA--------------AVEGIFFSGS-FCAIFWIKKRS-----------LMPGLTFSNELISRDEGLHTDFACLLYKHLVN----------KMS--NQRIHEIISSAVEIEKEFISEALP----VELIGMNS----------NLMKQYIEFVADRLIFALGAPKIY

Tet_thermophila_XP_001024960 ------NFASRLIAFA--------------AIEGIFFSGS-FCAIFWLKKRS-----------LMPGLTFSNELISRDEGLHTDFACLLYSMLNK----------KLT--KEEIYEIITEAVEIEKEFISEALP----VELIGMNS----------NMMKQYLEFVADRLLFALGVPKFY

Ara_thaliana_NP_189000 ------SFAVRLVAFA--------------CVEGIFFSGS-FCAIFWLKKRG-----------LMPGLTFSNELISRDEGLHCDFACLLYSLLQK----------QLP--LEKVYQIVHEAVEIETEFVCKALP----CDLIGMNS----------NLMSQYIQFVADRLLVTLGCERTY

Pop_trichocarpa_EEE89193 ------VFAERLVAFA--------------CIEGIFFSGS-FCAIFWLKKRG-----------LMPGLTFSNELISRDEGLHCDFACLLYSLLQR----------QLH--WQKVYHIVGEAVEIETKFVCEALP----CALIGMNA----------TLMGDYIKFVADRLLVALGYQKKY

Ara_thaliana_NP_189342 ------TFAERIIAFA--------------CVEGIFFSGS-FCSIFWLKKRG-----------LMPGLTFSNELISRDEGLHCDFACLLYTLLKT----------KLS--EERVKSIVCDAVEIEREFVCDALP----CALVGMNR----------DLMSQYIEFVADRLLGALGYGKVY

Pop_trichocarpa_EEE77642 ------SFAERLVAFA--------------CVEGIFFSGS-FCAIFWLKKRG-----------LMPGLTFSNELISRDEGLHCDFACLLYSLLRK----------KLS--EEQVKGIVKDAVDIEREFVVDALP----CALVGMNG----------ELMSQYIEFVADRLLGALGCGKVY

Pop_trichocarpa_EEE83435 ------SFAERLIAFA--------------CVEGIFFSGS-FCAIFWLKKRG-----------LMPGLTFSNELISRDEGLHCDFACLLYSLLRK----------KLS--EERVKGIVKEAVDIEREFVVDSLP----CALVGMNG----------ELMSQYIEFVADRLLGALGCGKVY

Ory_sativa_ACC95435 ------RFAERLVAFA--------------CVEGIFFSGS-FCAIFWLKKRG-----------LMPGLTFSNELISRDEGLHCDFACLLYDLLRG----------KLD--EARVREIVADAVDIEREFVCDALP----VALVGMNG----------DLMSQYIEFVADRLLMALGCKKMY

Zea_mays_NP_001130908 ------RFAERLVAFA--------------CVEGIFFSGS-FCAIFWLKKRG-----------LMPGLTFSNELISRDEGLHCDFACLLYDLLRG----------KLD--ESRVREIVADAVDIEREFVCDALP----VALVGMNG----------GLMSQYIEFVADRLLMALGHRKMY

Zea_mays_NP_001131892 ------RFAERLVAFA--------------CVEGIFFSGS-FCAIFWLKKRG-----------LMPGLTFSNELISRDEGLHCDFACLLYDVLRS----------KLD--EARVRTIVADAVDIEREFVCDALP----VSLVGMNG----------DLMSQYIEFVADRLLMALGCKKMY

Zea_mays_NP_001150842 ------RFAERIVAFA--------------CVEGIFFSGS-FCAIFWLKKRG-----------LMPGLTFSNELISRDEGLHCDFACLLYELLRN----------KLD--EARVREIVADAVDIEREFVCDALP----VALVGMNG----------DLMSQYIEFVADRLLMALGCKKMY

Ory_sativa_NP_001056668 ------RFAERLVAFA--------------CVEGIFFSGS-FCAIFWLKKRG-----------LMPGLTFSNELISRDEGLHCDFACLLYDLLRG----------KLD--ESRVQEIVADAVDIEREFVCDALP----VALVGMNG----------ELMSQYIEFVADRLLMALGCKKMY

Per_marinus_XP_002786498 ------CFAERLIAFA--------------CVEGILFSGS-FCAIYWLKKRG-----------LMPGLTFSNELISRDEGLHADFACLLYNMLKYT---------RLP--DERVHEIVRGAVEVERVFISESLP----VSLIGMNS----------QLMCRYIEFVADRLLVALGHPKLY

Per_marinus_XP_002773236 ------CFSERLIAFA--------------CVEGILFSGS-FCAIYWLKKRG-----------LMPGLTFSNELISRDEGLHADFACLLYNMMHYT---------RLP--DERVHEIVRGAVDVERVFISESLP----VSLIGMNS----------QLMCRYIEFVADRLLVALGHPKLY

Bab_bovis_XP_001610982 ------SFAERIVAYA--------------AVEGIFFSGS-FCAIFWLKKRG-----------LMPGLTFSNELIARDEGLHADFGCFIYSKLKH----------KLP--AECVQQIIKEAVAVERVFVCDSLP----TDLIGMNS----------MLMAQYIEFVADRLLQALGVPPVY

Bab_equi_6m007342 ------AFAERIVAYA--------------AVEGILFSGS-FCALFWLKKRG-----------LMPGLTFSNELISRDEGLHADFGCFIYQNLKH----------KLP--NEAVQQIIKEAVIVERSFICDSLP----CDLIGMNS----------QLMIQYIEFVADRLLKLLGVPPVY

The_annulata_XP_954052 ------SFAERIVSNFKNKLINYYRIVAYAAVEGILFSGS-FCALFWLKKRG-----------LMPGLTFSNELIARDEGLHADFGCFIYHNLKH----------KLP--EEAVQQIIKEAVVVERSYICDSLP----CDLIGMNS----------QLMTQYIEFVADRLLQSLGVRPVY

The_parva_XP_766246 ------SFAERIVAYA--------------AVEGILFSGS-FCALFWLKKRG-----------LMPGLTFSNELIARDEGLHADFGCFIYHNLKH----------KLP--EEAVQQIIKEAVVVERSYICDSLP----CDLIGMNSRKKILTHCLIELMTQYIEFVADRLLQALGVRPVY

Cry_hominis_XP_665115 ------SFAERIIGFC--------------AVEGILFSGS-FCAIFWLKKRG-----------LMPGLTFSNELISRDEGLHADFACLIYKALKF----------KLP--APRVQQIISEAVEVERNFLCDSLP----VDLIGMNS----------RLMAQYIEFVADRLLFALDIPKIY

Cry_parvum_XP_627447 ------SFAERIIGFC--------------AVEGILFSGS-FCAIFWLKKRG-----------LMPGLTFSNELISRDEGLHADFACLIYKALKF----------KLP--APRVQQIISEAVEVERNFLCDSLP----VDLIGMNS----------RLMAQYIEFVADRLLFALDIPKIY

Cry_muris_XP_002140093 ------SFAERIIGFC--------------AVEGILFSGS-FCAIFWLKKRG-----------LMPGLTFSNELISRDEGIHADFGCLLYKTLNN----------RLP--YGRVQEIIAEAVEVERAFLCDSLP----VDLIGMNS----------RLMAQYIEFVADRLLFALDVPKIY

Neo_caninum_NCLIV_052980 ------CFAERLVGFA--------------CVEGILFSGS-FCSIFWLKKRG-----------LMPGLTFSNELISRDEGLHADFACLLYSQLKN----------KLP--DKVVQDMVREAVTVERAFICEALP----VDLIGMNN----------RSMAQYIEFVADRLLTSLGVPKIF

Tox_gondii_XP_002371991 ------CFAERLVGFA--------------CVEGILFSGS-FCAIFWLKKRG-----------LMPGLTFSNELISRDEGLHADFACLLYSQLKH----------KLP--DKVVQDMVREAVTVERAFICEALP----VDLIGMNN----------RSMAQYIEFVADRLLTSLGVPKIF

Dic_discoideum_XP_644369 ------SFAERLVAFA--------------AVEGIFFSGS-FCSIFWLKKRG-----------LMQGLTFSNELISRDEGLHCDFACLLYTKLQR----------KLD--PKVIEKMIRDAVECEKEFICESLP----VDLIGMNS----------RSMSQYIEFCADRLVVSLGYKKIF

Cae_elegans_NP_497821 -----ASFAERLIAFA--------------AVEGIFFSGS-FASIFWLKKRG-----------LMPGLTHSNELISRDEGLHRDFACLLYSKLQK----------KLT--QQRIYDIIKDAVAIEQEFLTEALP----VDMIGMNC----------RLMSQYIEFVADHLLVELGCDKLY

Enc_cuniculi_NP_585829 -----SDFATRLVAFA--------------CVEGIFFSGA-FASIFWLKKRG-----------LMPGLTFSNELISRDEGLHCEFACLLHYHLKK----------KCN----RIKEVVMDAVEIEKKFLSESLP----VNLIGMNC----------NLMCRYIEFVADRLLENLGEERVY

Pla_berghei_Pdb_103660 ------SFAERIVANA--------------CVEGILFSGS-FCAIFWFKKQN-----------KLHGLTFSNELISRDEGLHTDFNCLIYSLLEN----------KLP--EEVVQNIVKEAVEVERSFICESLP----CDLIGMNS----------RLMSQYIEFVADRLLECLGCSKVF

Pla_chabaudi_XP_739266 ------SFAERIVANA--------------CVEGILFSGS-FCAIFWFKKQN-----------KLHGLTFSNELISRDEGLHTDFNCLIYSLLEN----------KLP--EEVVQNIVKEAVEVERSFICESLP----CDLIGMNS----------RLMSQYIEFVADRLLECLGCSKVF

Pla_yoelii_XP_723858 ------SFAERIVANA--------------CVEGILFSGS-FCAIFWFKKQN-----------KLHGLTFSNELISRDEGLHTDFNCLIYSLLEN----------KLP--ENVVQNIVKEAVEVERSFICESLP----CDLIGMNS----------RLMSQYIEFVADRLLECLGCSKVF

Pla_falciparum_XP_001348226 ------SFAERIVANA--------------CVEGILFSGS-FCAIFWFKKQN-----------KLHGLTFSNELISRDEGLHTDFNCLIYSLLDN----------KLP--EQMVQNIVKEAVEVERSFICESLP----CDLIGMNS----------RLMSQYIEFVADRLLECLGCSKIF

Pla_reichenowi_novel_model_330 ------SFAERIVANA--------------CVEGILFSGS-FCAIFWFKKQN-----------KLHGLTFSNELISRDEGLHTDFNCLIYSLLDN----------KLP--EQMVQNIVKEAVEVERSFICESLP----CDLIGMNS----------RLMSQYIEFVADRLLECLGCSKIF

Pla_gallinaceum_rna_PF_0053_1_1cds ------SFAERIVANA--------------CVEGILFSGS-FCAIFWFKKQN-----------KLHGLTFSNELISRDEGLHTDFNCLIYSLLEN----------KLP--EEVVQNIVKEAVEVERSFICESLP----CDLIGMNS----------RLMSQYIEFVADRLLECLGCSKVF

Pla_knowlesi_XP_002260936 ------SFAERIVANA--------------CVEGILFSGS-FCAIFWFKKQN-----------KLHGLTFSNELISRDEGLHTDFNCLIYSLLEN----------KLP--EEVVQNIVKEAVEVERSFICESLP----CDLIGMNS----------RLMSQYIEFVADRLLECLGSRKIF

Pla_vivax_XP_001616894 ------SFAERIVANA--------------CVEGILFSGS-FCAIFWFKKQN-----------KLHGLTFSNELISRDEGLHTDFNCLIYSLLEN----------KLP--EEVVQNIVKEAVEVERSFICESLP----CDLIGMNS----------RLMSQYIEFVADRLLECLGSPKIF

Sch_pombe_NP_596546 -----STYAIRLVAFA--------------AVEGIFFSGS-FASIFWLKKRG-----------LMPGLTFSNELICRDEGLHTDFACLMFSHLKH----------RPG--RKVVEAIIVEAVDIEKEYFTDALP----VSLLGMNK----------DLMCQYIEFVADRLLVALGNDKYY

Sac_cerevisiae_S288c_NP_011696 -----SLYAERLVAFA--------------AKEGIFQAGN-YASMFWLTDKK-----------IMPGLAMANRNICRDRGAYTDFSCLLFAHLRT----------KPN--PKIIEKIITEAVEIEKEYYSNSLP----VEKFGMDL----------KSIHTYIEFVADGLLQGFGNEKYY

Per_marinus_XP_002768004 ------CFAERLVAYA--------------CVEGILFSGS-FCALFWLKKRG-----------LMPGLTFSNELISRDEGLHTDFGCLLYNMLKFT---------RLP--EERVEDIVRSAVNVEREFITDALP----VGLIGMNA----------ELMGRYIEFVADRLLVALGYAKIY

Cae_elegans_NP_500944 -----APFSERLAAVA--------------AVEGIFFSSS-FAAIFWLKKRG-----------LLPGLTHSNELISRKPA------------------------------ESRITEIINESVLVEQDFVRESLP----TNLIGMNQ----------HMMCKYVEFVADLLLEELGYSKRY

Cae_elegans_NP_508269 -----APFSERLAAFA--------------AVEGIFFSSS-FAAIFWLKKRG-----------LLPGLTHSNELIS-MFLLHPNCFFSTFNLQRR-----------RKPAESRITEIINESVLVEQDFVRESLP----TNLIGMNQ----------HMMCKYVEFVADLLLEELGYSKRY

Dic_discoideum_XP_629985 --------EKRLVALV--------------AFESIFFSGS-FRIISLLKKRS-----------LMQGLTFSNELIN--------------------------------------------------------------------------------------------------------

*: . . *.:: :. : : :..: : **: :*. *. : : . : :: :: * : .::* .**: : *::* :* *: :. :

(Cae_elegans_NP_500944 & Dic_discoideum_XP_629985 excluded)

Högbom 2010 positions 14 15 16 17 18 19 20 21 22 23 24 25 | 26 27 | | |28

Högbom 2010 exceptions |* ** * **| || * | | |found | | |*

Baci_halodurans_NP_241368 --PTPKTFLESIVYDV--------------ILEGLNFYSG-FAFFYNLARNQ-----------KMVSTSTMINYINRDEQLHVYLFTNIFKELLVEFP-----ELNTEETKTFVKTTLMKAADLEKDWFRYIIG----DKIPGINP----------EDMETYISFIANKRAVQLGMEKPY

Pae_sp_ZP_04851883 ---TPQSFFEALVADL--------------ILEGIFFYST-FAFFYNLARDQ-----------KMMGTSQMISYIQRDENQHCYFFAEVFKQLLIDYP-----ELNTKENIDYAYRTIDRAVELETNWAHYTLKN---VPGIDLN------------ELSDYIKYIANKRLTLMGLEKAY

Bact_vulgatus_YP_0130035 ----GFTLMKTLIANY--------------ILEGIYFYSG-FMFFYNLSRNG-----------KMSGSAQEIRYINRDENTHLWLFRNIILELKKEEPD-----LFTPDKVKIYEYMMREGVKQEIEWGQYVIG----DNIQGLNR----------KMIEDYIQYLGNLRWSSLGFGPLY

Clo_botulinum_YP_002805314 ---EIHNFLRVIMANY--------------ILEGIYFYSG-FSFFYTLARQG-----------KMTATSTIFKYINRDEVTHLVLFQNIIKELKNENP-----HIFTEELEEEFRQMMRIGVEHEIQWGQYVTN----NEILGLND----------ELIEKYIKYLSNLRLVAIGLKPLY

Cya_sp_ATCC_51142_YP_001803056 ----PENYFIALMADF--------------LLEGLYFYNG-FIYFYNLASRM-----------LMPGSADIFKMINRDELSHVRLYQKLIPEARK----------VFPHSVEQIYEMFDMAVNYECRWTNHIVG----NNILGITES----------STEKYTKYLANIRLRSIGLEPLY

Cya_sp_CCY0110_ZP_01726237 ----SENYFIALMADF--------------LLEGLYFYNG-FIYFYNLASRM-----------LMPGSADIFKMINRDELSHVRLYQKLIPEARK----------VFPHSVEQIYEMFDMAVNYECRWTNHIVG----NNILGITES----------STEKYTKYLANIRLRSIGLEPLY

Cya_sp_ATCC_51142_YP_001806290 -----ETYFMALIGDY--------------LLEGLLFYQG-FLFFYNLASSQ-----------RLAGCAKLIKQINMDELTHVSLYQKLITEAKT----------KFSYSEDEITELFVNTVEKEIEWTNHIFDG----QILGVSE----------ASTEAYTKYLANLRLKAIGISAIY

Cya_sp_CCY0110_ZP_01729893 -----ENYYMALIGDY--------------LLEGLLFYQG-FLFFYNLASSQ-----------RLAGCAKLIKQINMDELTHVSLYQKLITEATT----------KFSYSEDEITELFHTTVEREIEWTNHIFDG----QILGVSK----------ASTEAYTKYLANLRLKAIGIPAIY

Cau_cresents_NP_419079 -----ADICRTLAMFG-------------GFTEGLQLFAS-FAMLMNFPRFN-----------KMKGMGQIVSWSIRDESLHCDGIIKLYHAFNKETG------AVTKAVADDIVDCCKTVVGMEDNFIDLAFEAG---DIQGMTP----------DDIKAYIRFVADWRLRQLHLPEVY

Cau_sp_YP_001686327 -----ADIARTLAMFG-------------GFTEGLQLFAS-FAMLMNFPRFN-----------KMKGMGQIVSWSIRDESLHCEGIIKLYHAFNAET------KCVTPEVAADIVECCKSVVIMEDAFIDLAFEAG---EIQGMTP----------HDIKQYIRFIADWRLRQLHLPEVY

Neo_sennetsu_YP_506404 -----TEIAKTLAVYG-------------AFTEGLQLFAS-FAILLNFPRFN-----------KMKGMGQIIAYSVRDETLHSNSITKLFRTFLLENN------IDKFKLEAEIREICNAMVHHEDAFIDLAFEMG---DVEGLKA----------SEVKEYIRYIANLRLKQLGFSEIY

Ori_tsutsugamushi_YP_001248105 -----EEIATTLAVFG-------------AFTEGVQLFAS-FAILLNFPRYN-----------KMKGMGQIITWSVRDETLHTNSIITLFKTFIQEYP-----EVWTEQLKSRIYEACCTIIHFEDAFIDLAFEVG---GVEGLEA----------RDVKKYIRYIADRRLLQLGLKENY

Ric_rickettsii_YP_001494766 -----EDIATTLAVFG-------------AFTEGLQLFAS-FAIMLNFPRFN-----------KMKGMGQIIAWSVRDETLHTNSIITLFKTFVRENPE-----VWTETLRKRIYEACTTIVHFEDAFIELAFEVG---GIEGLTA----------YEVRQYIRYIADRRLMQLGLKDVY

Wol_pipientis_YP_001974856 ------HVAKTLAVFG-------------AFTEGLQLFAS-FAILLNFQRFG-----------KMKGMGQIIAWSARDETLHTNSIINLFNTFIKENNE-----IWNDEFKEELYSACRTIVELEDEFISLAFDLG---DVEGLSA----------EEVRNYIRYIANRRLTQLGLKSIY

Wol_sp_NP_966023 --HDKKHVAKTLAVFG-------------AFTEGLQLFAS-FAILLNFQRFG-----------KMKGMGQIIAWSARDETLHTNSIIMLFNTFIKENNE-----IWDNEFKEELYSACRTIVELEDEFIKLAFDLG---DVEGLSA----------EEVRNYIRYVANRRLMQLGLESIY

Bau_cicadellinicola_YP_588829 QHELKKKLYLCLMSIN--------------ALEAIRFYVS-FACSFAFAERK-----------LMEGNAKIIRLIARDEALHLAGTQHMLNLMRLGKDDPEMADIALE-CQHDSYHLFLAANQ-EKDWASYLFR---DGSMIGLNE----------EILCQYIEYITNIRMQAVLLDLPF

Esc_coli_YP_001731173 LRELKKKLYLCLMSVN--------------ALEAIRFYVS-FACSFAFAERE-----------LMEGNAKIIRLIARDEALHLTGTQHMLNLLRSGADDPEMAEIAEE-CKQECYDLFVQAAQQEKDWADYLFR---DGSMIGLNK----------DILCQYVEYITNIRMQAVGLDLPF

Shi_dysenteriae_YP_403993 LRELKKKLYLCLMSVN--------------ALEAIRFYVS-FACSFAFAERE-----------LMEGNAKIIRLIARDEALHLTGTQHMLNLLRSGADDPEMAEIAEE-CKQECYDLFVQAAQQEKDWADYLFR---DGSMIGLNK----------DILCQYVEYITNIRMQAVGLDLPF

Sod_glossinidius_YP_455265 LHELKKKLYLCLMSVN--------------ALEAIRFYVS-FACSFAFAERE-----------LMEGNAKIIRLIARDEALHLTSTQHMLNLMRSGEDDPEMAEIARE-CQQESYDLFLLAAQQEKEWASYLFR---DGSMIGLNK----------DILCQYVEYITNIRMQSVGLDLPF

Yer_pestis_A_ZP_04509308 LRDLKKQLYLCLMSVN--------------ALEAIRFYVS-FACSFAFAERE-----------LMEGNAKIIKMIARDEALHLTGTQHILNLMRLGEDDPEMAEIAEE-CQQQCYDLFVLAAQQEKEWAEYLFR---DGSMIGLNK----------EILCQYVEYITNIRMQAVGLGIPF

Buc_aphidicola_NP_240009 LRFLKKRLYLCLISVN--------------VLEAIRFYVS-FACSFAFAERE-----------LMEGNAKIIRLIARDEALHLTGTQHILNLLSNIKNN-ENMEDVVLECKEEAINIFISAAQQEKKWASYLFK---SGSMLGLNK----------DILCQYIEYITNIRMHAIGFKMPF

Yer_pestis_A_ZP_04512133 PYPLQRQLFRTLVSVN--------------ALEAIRFYVS-FACTFAFGERK-----------LLEGNTKIMRFIARDEALHCEGTERMIRFMRTGREGLMWKMIAAE-EESFIYQTMMDVAGQEMRWADYLFK---DGSMIGLNA----------DILKSYVKYRTNLAMRRLGLRPLF

Halom_mukohataei_ZP_03875489 -----QEYVMSLLLFS-------------TFVEHVSLFSQ-FLIMTSFDKHEK----------KFKGIANAVEATSKEEQIHGLFGVELVETIREENPD-----LFDEDFEEEVQAACQQAFEAEMKILDWIFGE---GELEFLPR----------AHVDAFLRDRFNQSLENVGVEPIF

Natro_pharaonis_YP_327710 -----QEYVMSILLFS-------------TFVEHVSLFSQ-FLIMMSFDKYKK----------KFKGIANAVEATSKEEQIHGLFGVELVETIREENPD-----LFDEDFEEEVQAACQRAFEAEMKILDWIFGE---GELEFLPR----------THVDAFLRDRFNQSLENVGVEPIF

Halor_lacusprofundi_YP_002564382 -----KGYVMSILLFS-------------MFVEHVSLFSQ-FLIMTSFDKYEK----------KFKGIANAVEATSKEEQIHGLFGVELVDTIREENPG-----LFDDEFEAEIREACRRAYEAEMGMLDWIFGE---GELQFLPR----------EYVNEFLKDRFNQSLENVGVYPMF

: * : :: * : : . . :* * : * : : : : : . :

Högbom 2010 positions 14 15 16 17 18 19 20 21 22 23 24 25 | 26 27 | | |28

Högbom 2010 exceptions |* ** * *|| || * * | *found | | |*

Chl_muridarum_NP_296594 -VEGLQEFIKNLVGYY-------------IIMEGIFFYSG-FVMILSFHRQN-----------KMVGIGEQYQYILRDETIHLNFGVDLINGIKEEN-----PEIWTTELQQEIIEMIQRAVDLEIDYARDCLP----RGILGLRA----------SMFIDYVQHIADRRLERIGLKPIY

Chl_trachomatis_YP_328659 -VEGLQEFVKNLVGYY-------------IIMEGIFFYSG-FVMILSFHRQN-----------KMIGIGEQYQYILRDETIHLNFGIDLINGIKEEN-----PGIWTPELQQEIVELIKRAVDLEIEYAQDCLP----RGILGLRA----------SMFIDYVQHIADRRLERIGLKPIY

Halob_sp_NP_280997 ------DFLRDLVGFY-------------VIMEGIFFYAG-FAMMLALKRRG-----------KMVGVGEQFEYIMRDESLHLNFGVDLINQIRDEHP-----DVWTADFEAEVNSLMRDAVDLEQLYARDACP----DDLLGMSS----------EQFADYVEYVADRRFQQLRMDPVY

Halog_borinquense_ZP_04000565 DTADVRDLLRDLVGFY-------------VVMEGVFFYAG-FAMMLALKRQN-----------KMVGIGQQFEYIMRDESLHVGFGVDLINQIRAENP-----DAWTDEFGAEVRDLIEKAVDLEQIYAREACP----DDVLGMSP----------DQFAEYVEHVADRRLGQLDLDASY

Halom_utahensis_YP_003131236 ----VRDLLRDLVGFY-------------VIMEGIFFYAG-FAMMLGLKRQN-----------KMVGIGQQFEYIMRDESLHVGFGVDLIDQIRTENPG-----VWTDEFDSEVIDLIKEAVELEQIYAREACP----EEILGMSG----------SQFADYVEYIADRRLDQLDLPEQY

Natri_magadii_ZP_03692956 TDEDLRDFIRDLVGFY-------------VIMEGIFFYAG-FAMMLGLKRQN-----------KMVGIGQQFEYIMRDESLHVGFGVDLIDQIRMENPD-----VWTDAFGDEVVELITEAVELEKIYAYEACP----DEILGMSA----------DQFAEYVEHIADRRLGQLDLPTQY

:::::***:* ::***:***:* *.*:*.::*:. **:*:*:*::**:***::*:.**:***: *: *: ** : *: .:: **:** ** :. * :**: . * :**:::****: :: : *

Högbom 2010 positions 14 15 16 17 18 19 * 20 21 22 23 24 25 | 26 27 | | |28

Högbom 2010 exceptions |* ** * * *|| || * | | |found | | |*

Geo_kaustophilus_YP_148624 -----EAVIRAATVYN-------------MIVEGTLAESG-YYTFRQIYKKAG----------LFPGLLQGIDYLNMDEGRHIQFGIYTIQRIVNE--------------DERYYELFIRYMDELWPHVIGYVD---YLTELGKRQ----------QQLARTYALEIDYDLLRHYVIKQF

Geo_sp_ZP_03557705 -----EAVIRAATVYN-------------MIVEGTLAESG-YYTFRQIYKKAG----------LFPGLLQGIDYLNMDEGRHIQFGLYTIQRLVNE--------------NERYYDLFIQYMDELWPHVVGYVAY---LTELGKRQ----------QQLARTYALEIDYDMLNNYVVKQF

Myc_avium_NP_962606 ----PAAQVRASVTYN-------------HMVEGMLALTG-YFGWHKICVERG----------ILPGMQELVRRIGDDERRHMAWGTFTCRRHVAAD--------DAN--WGVFETRMNELMPLGLRLIEEGFALY-DPMPFDLSV----------DEFMAYASDKGMRRFGTIASARGR

Myc_bovis_NP_853903 ----PAAQVRASVTYN-------------HIVEGMLALTG-YYAWHKICVERA----------ILPGMQELVRRIGDDERRHMAWGTFTCRRHVAAD--------DAN--WTVFETRMNELIPLALRLIEEGFALYGDQPPFDLSK----------DDFLQYSTDKGMRRFGTISNARGR

Myc_tuberculosis_NP_214747 ----PAAQVRASVTYN-------------HIVEGMLALTG-YYAWHKICVERA----------ILPGMQELVRRIGDDERRHMAWGTFTCRRHVAAD--------DAN--WTVFETRMNELIPLALRLIEEGFALYGDQPPFDLSK----------DDFLQYSTDKGMRRFGTISNARGR

Nat_pharaonis_YP_331256 AAPTAEQFAKAYCHYH-------------LVIEGILAQTG-YYGMQQSYSADSYPELP-----HLPGLCDGFTKIRQDEGRHVGFGMAKLKALVG----------DGKVDPQLLDDIVGELLPLVDDITTDVGDES--FDDVGIPP----------EELEAFAAEKHTERMEQIKD---A

Nat_pharaonis_YP_330945 --DTPTNRVRAYCHYH-------------LTVESVLAQTG-YWGIVSSMSPRGSEALREDGMPHLEGLVKGISFIRSDEGRHVGFGMQKVQSHLA--------------EDGVDESVVRETLQELLPLVAETVS-----ATDEVTD---------PEPLVEYASEKLTRRIEVITD---A

Sul_islandicus_YP_002913609 ------NQVGAVVTYN-------------LIVEGVAAEGG-YNIFRQITNTRK----------ILPGLAKMVNLIATDESRHIAFGIYLITRLVKEYG--------EGVYKAAMDHINYLAPYAIGIFSEPTMP---QVESLPFNLTN--------IELVDYAKKLLNTRIDAINRAKRM

Sul_solfataricus_NP_343843 ------NQVRAVVTYN-------------LIVEGVAAEGG-YNIFRQITNTRK----------ILPGLAKMVNLIATDESRHIAFGIYLITRLVKEYG--------EGVYKAAMDHINYLAPYAIGIFSEPTMP---QVESLPFNLTN--------IELVDYAKKLLNTRIDAINRARRM

* *: :*. * * * . : *: . . : ** **: :* : : :

1JK0:A S. cerevisiae Y2

...

GGGG

**1**SMQ:A S. cerevisiae STT

1JK0:B S. cerevisiae Y4

1SMS:A S. cerevisiae S HHHHHHHTT

3HF1:A H. sapiens

2UW2:A H. sapiens S TT

2VUX:A H. sapiens TT

1H0N:A M. musculus TTT

2O1Z:A P. vivax TTTT

2P1I:A P. yoelii S

2RCC:A B. halodurans TT S S TT

2ALX:A E. coli S GGGGG

1AV8:A E. coli S GGGGG------------------------------------T

1JPR:A E. coli S TTHHH------------------------------------HH

1JQC:A E. coli S GGGHH------------------------------------HH

1MXR:A E. coli S GGGGG------------------------------------T

1SYY:A C. trachomatis S TT

2ANI:A C. trachomatis S TT

3EE4:A M. tuberculosis G-GGT

S. cerevisiae S288c NP 012508 (Y2) 350 360 370 380 390 399

coordinates . . . . . .

matrix position 820 830 840 850 860 870 880 890 900 910 920

. . . . . . . . . . .

E. coli electron transfer pathway ⧫ ⧫

S. typhimurium electron transfer pathway ◊

conserved residues (Wang 1997) ↓ ↓

Högbom 2010 positions || 29 30

Högbom 2010 exceptions || |||| *

Bab_bovis_XP_001610573 R----VKVELGWLKP--------------------------------------SIFTGNASTQVRQIQRKIQPVEENS------------------GGSISFNEDF----

Bab_equi_6m007985 K----PDLDVDWINEFS------------------------------------VDLNIQLNISKPSIQHDIEHFEQ----------------------EIKFDEDF----

The_annulata_XP_953574 T----SEFDIDWLNHP----------------------------------------IIDLNIQKQTNKIQKHNENLN---------------------DIKFDEDF----

The_parva_XP_766717 S----SDFDVEWLNHP------------------------------------------TVDLNIRQVIKKQKTEHTT---------------------EIKFDEDF----

Pla_berghei_Pdb_121420 N----SKYPFTWKEFS--------------------------------------KIVVSENAKNESVKKSSDLYGEK---------------------QITFDEDF----

Pla_yoelii_XP_727957 N----SKYPFAWKEFSKVLSFYTLRCNFYHFTLPIFTTLRCNFCYLFISTTFYFQIVVSENAKNESVKKSSDLYGEK---------------------QITFDEDF----

Pla_chabaudi_PCAS_121490 N----SKYPFTWKEFS--------------------------------------KIIVSENAKNDSVKKSADLYGAK---------------------QITFDEDF----

Pla_falciparum_XP_001347439 N----SKYPFSWPEFT--------------------------------------KIIVNENGKEETVKKGENVYGEK---------------------QILFDEDF----

Pla_knowlesi_XP_002258799 N----SKYPFTWPEFN--------------------------------------KIVVSENQKTESVKKAEDIYGER---------------------QIVLDEDF----

Pla_vivax_XP_001614470 N----SKYPFTWPEFN--------------------------------------KIVVSENQKAESVKKTGDIYGER---------------------QIVLDEDF----

Cry_hominis_XP_665685 N----VSNPFTNIMNDFPAPSQ------------------------------EEAVARQTLTQTLKAAHYRTYQEISS-------------------NQFSTSQDF----

Cry_parvum_XP_001388304 N----VSNPFINIMN------------------------------------------DFPCSFSRRSCC-----------------------------------------

Cry_muris_XP_002140092 N----VSNPFSELIS--------------------------------------DLPAPSQEEAAARQTLTQTLKVIH----------CKTYQEITDALQFSTSQDF----

. . : .:**

(Cry_parvum_XP_001388304 excluded)

Högbom 2010 positions || 29 30

Högbom 2010 exceptions || |||| *

Ano_gambiae_XP_308927 H----TKNPFSFMEF--------------------------------------ISLEGKTNFFEKKVGEYQKWGVMA----------NRLD------NVFTLDADF----

Dro_melanogaster_NP_525111 N----TKNPFNFMEM--------------------------------------ISLDGKTNFFEKKVGEYQRMGVVS----------NPLD------NVFTLDADF----

Dan_rerio_NP_571525 R----VENPFDFMEN--------------------------------------ISLEGKTNFFEKRVGEYQRMGVMS----------GTTD------NTFTLDADF----

Gal_gallus_XP_001231545 K----VENPFDFMEN--------------------------------------ISLEGKTNFFEKRVGEYQRMGVMS----------KPTD------NSFTLDAEF----

Hom_sapiens_isoform_2_NP_001025 R----VENPFDFMEN--------------------------------------ISLEGKTNFFEKRVGEYQRMGVMS----------SPTE------NSFTLDADF----

Mus_musculus_NP_033130 R----VENPFDFMEN--------------------------------------ISLEGKTNFFEKRVGEYQRMGVMS----------NSTE------NSFTLDADF----

Rat_norvegicus_NP_001020911 K----VENPFDFMEN--------------------------------------ISLEGKTNFFEKRVGEYQRMGVMS----------NSTE------NSFTLDADF----

Xen_laevis_NP_001079369 K----ATNPFDFMEN--------------------------------------ISLEGKTNFFEKKVGEYQKMGVMS----------KAKD------NTFTLDADF----

Xen_Silurana_tropicalis_NP_001007890 K----ATNPFDFMEN--------------------------------------ISLEGKTNFFEKKVGEYQKMGVMS----------KPKD------NTFTLDADF----

Xen_laevis_NP_001080772 K----ASNPFDFMEN--------------------------------------ISLEGKTNFFEKRVGEYQKMGVMS----------KPKD------NTFTLDADF----

Xen_laevis_NP_001085389 K----SENPFDFMEN--------------------------------------ISLEGKTNFFEKKVGEYQKMGVMA----------KATE------NQFTLNADF----

Xen_Silurana_tropicalis_NP_989048 K----SENPFDFMEN--------------------------------------ISLEGKTNFFEKKVAEYQKMGVMS----------KATD------KQFTLNAEF----

Gal_gallus_XP_418364 H----AENPFDFMEN--------------------------------------ISLEGKTNFFEKRVSEYQRFAVMA----------ETMD------NVFTLDADF----

Hom_sapiens_isoform_1_NP_056528 Q----AENPFDFMEN--------------------------------------ISLEGKTNFFEKRVSEYQRFAVMA----------ETTD------NVFTLDADF----

Mus_musculus_NP_955770 Q----AENPFDFMEN--------------------------------------ISLEGKTNFFEKRVSEYQRFAVMA----------ETTD------NVFTLDADF----

Rat_norvegicus_NP_001124015 Q----AENPFDFMEN--------------------------------------ISLEGKTNFFEKRVSEYQRFAVMA----------ETTD------NVFTLDADF----

Xen_Silurana_tropicalis_NP_001119973 K----AENPFDFMEN--------------------------------------ISLEGKTNFFEKRVSEYQRFAVMA----------HTED------NVFTLDADF----

Dan_rerio_NP_001007164 C----SENPFDFMES--------------------------------------ISLEGKTNFFEKRVAEYQRLGVMS----------NVKD------CEFTLDADF----

Dap_pulex_GNO_1472053 N----SENPFDFMEH--------------------------------------ISLEGKTNFFEKKVGEYQKAGVMN----------SKGNS----AHVFTLDEDF----

Asp_clavatus_XP_001274524 N----SPNPFDFMES--------------------------------------ISLAGKTNFFEKRVGDYQKAGVMA---STKK-DTNQDAAKDTNAGGLSFDEDF----

Neu_crassa_XP_962820 R----STNPFDFMEN--------------------------------------ISLGGKTNFFEKRVGDYQKAGVMN---STKKADADAEVAKNENGGDFTFDEDF----

Dap_pulex_GNO_1331594 N----TTNPFDFMDM--------------------------------------ISLQGKTNFFEKKVAEYQKAGVMN----------TDSDA-----QKISFDADF----

Can_albicans_XP_715277 N----VTNPFDFMEN--------------------------------------ISLAGKTNFFEKRVSDYQKAGVMA----------KTENK--E-ADAFTFDEDF----

Can_albicans_XP_713125 N----VTNPFDFMEN--------------------------------------ISLAGKTNFFEKRVSDYQKAGVME----------KVENK--N-EKTGLFDQDF----

Sac_cerevisiae_S288c_NP_012508 K----VENPFDFMEN--------------------------------------ISLAGKTNFFEKRVSDYQKAGVMS----------KSTKQ--E-AGAFTFNEDF----

Lei_braziliensis_XP_001565976 N----ATQPFDFMEM--------------------------------------ISLQGKTNFFEKKVGEYQKAGVMS----------AEGTS-----KKFSLSEDF----

Lei_braziliensis_XP_001565036 N----ATQPFDFMEM--------------------------------------ISLQGKTNFFEKKVGEYQKAGVMS----------AEGTS-----KKFSLSEDF----

Try_cruzi_XP_813233 N----SKNPFDFMEM--------------------------------------ISLQGKTNFFEKKVGEYQKAGVMS----------SEGTT-----KVFSLDAEF----

Par_tetraurelia_XP_001454302 N----SKNPFEWMDM--------------------------------------ISLQGKTNFFERRVGDYQKAGVMS----------KKQD------KVFTMDADF----

Tet_thermophila_XP_001024960 N----SKNPFEWMDM--------------------------------------ISIQGKTNFFEKRVGEYQKANVMS----------SKED------KVFRLNADF----

Ara_thaliana_NP_189000 K----AENPFDWMEF--------------------------------------ISLQGKTNFFEKRVGEYQKASVMS----------NLQNG--NQNYEFTTEEDF----

Pop_trichocarpa_EEE89193 N----VENPFDWMEF--------------------------------------ISLQGKANFFERRVGDYQKASVMS----------SLQDG--GKNYVFKMDEDF----

Ara_thaliana_NP_189342 G----VTNPFDWMEL--------------------------------------ISLQGKTNFFEKRVGDYQKASVMS----------SVNGNGAFDNHVFSLDEDF----

Pop_trichocarpa_EEE77642 N----VANPFDWMEL--------------------------------------ISLQGKTNFFEKRVGEYQKASVMS----------SINGN--GGNHVFKMDEDF----

Pop_trichocarpa_EEE83435 N----VANPFDWMEL--------------------------------------ISLQGKTNFFEKRVGEYQKASVMS----------SINGN--GDNHVFKMDEDF----

Ory_sativa_ACC95435 N----VANPFDWMEL--------------------------------------ISLQGKTNFFEKRVGDYQKASVMS----------SLNGG-ASANHVFSIDEDF----

Zea_mays_NP_001130908 N----VANPFDWMEL--------------------------------------ISLQGKTNFFEKRVGEYQKASVMS----------SLNGG-AAANHTFSIDEDF----

Zea_mays_NP_001131892 N----VTNPFDWMEL--------------------------------------ISLQGKTNFFEKRVGDYQKASVMN----------SLNGS-AAANHVFSIDEDF----

Zea_mays_NP_001150842 N----VTNPFDWMEL--------------------------------------ISLMGKTNFFEKRVGDYQKASVMN----------SLNGG-AAADHVFSIDEDF----

Ory_sativa_NP_001056668 N----VANPFDWMEL--------------------------------------ISLQGKTNFFEKRVGDYQKASVMS----------SLNGG-ASCNHVFSIDEDF----

Per_marinus_XP_002786498 N----ATNPFDWMKM--------------------------------------ISLQGKTNFFEKRVGEYQKGGVMAGTAAHKNSSMDAEERRKSIGADFALDEDF----

Per_marinus_XP_002773236 N----ATNPFDWMKM--------------------------------------ISLQGKTNFFEKRVGEYQKGGVMAGTAAHKNGSMDAEERRKSIGADFALDEDF----

Bab_bovis_XP_001610982 K----TKNPFEWMEL--------------------------------------ISVQGKTNFFEKRVGEYQKSGILA----------NQDE------QKFATDADF----

Bab_equi_6m007342 N----VTNPFDWMDL--------------------------------------ISVQGKTNFFEKRVGEYQKAGIMA----------NQNE------QKFSTDIEF----

The_annulata_XP_954052 N----VTNPFDWMQL--------------------------------------ISVQGKTNFFEKRVGEYQKAGIMA----------NHQE------QKFSTDLEF----

The_parva_XP_766246 N----VTNPFDWMQL--------------------------------------ISVQGKTNFFEKRVGEYQKAGIMA----------NHHE------QKFSTDLEF----

Cry_hominis_XP_665115 N----VSNPFDWMDL--------------------------------------ISLQGKTNFFEKRVGEYQMAGVMV----------NQDD------QKFDLNADF----

Cry_parvum_XP_627447 N----VSNPFDWMDL--------------------------------------ISLQGKTNFFEKRVGEYQMAGVMV----------NQDD------QKFDLNADF----

Cry_muris_XP_002140093 N----VTNPFDWMDL--------------------------------------ISLQGKTNFFEKRVAEYQMAGVMA----------HQKD------QIFNLDAEF----

Neo_caninum_NCLIV_052980 F----ATNPFDWMEL--------------------------------------ISLQGKTNFFEKRVGEYQKAGVMA----------DKEQ------QKFVLDADF----

Tox_gondii_XP_002371991 F----ATNPFDWMEL--------------------------------------ISLQGKTNFFEKRVGEYQKAGVMA----------DKDQ------QKFVLDADF----

Dic_discoideum_XP_644369 N----SSNPFEWMEM--------------------------------------ISLQRKSNFFEGKVAEYAKTGVAIQ---------GNNQQKNNQSRTLVLDEDF----

Cae_elegans_NP_497821 K----SKNPFDFMEN--------------------------------------ISIDGKTNFFEKRVSEYQRPGVMVN---------EAER-------QFDLEADF----

Enc_cuniculi_NP_585829 N----ATNPFDFMEN--------------------------------------ISLVGKTNFFDKRESQYQKAFVGIE---------NGND-------SFRIDVDF----

Pla_berghei_Pdb_103660 H----SKNPFNWMDL--------------------------------------ISLQGKTNFFEKRVADYQKSGVMA----------QRKE------QVFSLNTDF----

Pla_chabaudi_XP_739266 H----SKNPFSWMDL--------------------------------------ISLQGKTNFFEKRVADYQKSGVMA----------QRKE------QVFSLNTDF----

Pla_yoelii_XP_723858 H----SKNPFNWMDL--------------------------------------ISLQGKTNFFEKRVADYQKSGVMA----------QRKE------QVFSLNTDF----

Pla_falciparum_XP_001348226 H----SKNPFNWMDL--------------------------------------ISLQGKTNFFEKRVADYQKSGVMA----------QRKD------QVFCLNTEF----

Pla_reichenowi_novel_model_330 H----SKNPFNWMDL--------------------------------------ISLQGKTNFFEKRVADYQKSGVMA----------QRKD------QVFCLNTEF----

Pla_gallinaceum_rna_PF_0053_1_1cds H----SKNPFNWMDL--------------------------------------ISLQGKTNFFEKRVADYQKSGVMA----------QRKD------QVFCLNTDF----

Pla_knowlesi_XP_002260936 H----AKNPFNWMDL--------------------------------------ISLQGKTNFFEKRVADYQKSGVMA----------QRKD------QVFCLNSDF----

Pla_vivax_XP_001616894 H----AKNPFNWMDL--------------------------------------ISLQGKTNFFEKRVADYQKSGVMA----------QRKD------QVFCLNSDF----

Sch_pombe_NP_596546 N----VTNPFDFMEN--------------------------------------ISLAGKTNFFEKKVSDYQIAGVMSG--------TKRAEK---DDHTFTIDEDF----

Sac_cerevisiae_S288c_NP_011696 N----AVNPFEFMED--------------------------------------VATAGKTTFFEKKVSDYQKASDMS----------KSAT------PSKEINFDDDF--

Per_marinus_XP_002768004 N----SANPFDWMTM--------------------------------------ISLQGKTNFFEKRVERVPKRQCGG----------GGGKR----VRIASPAAE-----

Cae_elegans_NP_500944 N----TKNLFDFMEN--------------------------------------ISIEGKTNFF-----------------------------------------------

Cae_elegans_NP_508269 N----TKNPFDFMEN--------------------------------------ISIEGKTNFFEKRVSEYQRPEMMVD---------ESEK-------VFDLSALF----

Dic_discoideum_XP_629985 --------------------------------------------------------------------------------------------------------------

:**.:* :: *:.**: :

(The_annulata_XP_954052, The_parva_XP_766246, Cae_elegans_NP_500944 & Dic_discoideum_XP_629985 excluded)

Högbom 2010 positions || 29 30

Högbom 2010 exceptions || |||| *

Baci_halodurans_NP_241368 PE--IKHNPMKWIRA------------------------------------YEDVNSGKSDFFEQKSRQYAKVSA------------DNGF------DEL----------

Pae_sp_ZP_04851883 AG--VDINCMPWIKPF-SD---------------------------------EALNATKTDFFEAKSRNYGKVGDDN-----------------------GFDDL-----

Bact_vulgatus_YP_0130035 EENHKEPESMHWVSQY-S-----------------------------------NANMVKTDFFEAKSTAYAKS--------------------------TALEDDL----

Clo_botulinum_YP_002805314 LE--INKHPMEWIDSF-S-----------------------------------KLNSTKTDFFEAKVTNYTKAAAFD---------------------FDDLD-------

Cya_sp_ATCC_51142_YP_001803056 PEPKYNKSPYTHLERFSDT---------------------------------KKEAHTKANFFEATVTSYVMSSGVTG-----------------------WDEI-----

Cya_sp_CCY0110_ZP_01726237 PEPKYNKSPYTHLERFSDT---------------------------------KKEAHTKANFFEATVTSYVMSSGVTG-----------------------WDEI-----

Cya_sp_ATCC_51142_YP_001806290 PEVENPYAHLDWADFKG-------------------------------------IGKSKAAFFETEVTGYAQPTAVKG------------------WEKLRV--------

Cya_sp_CCY0110_ZP_01729893 PKIENPYAHLDWADFKG-------------------------------------IGKSKAAFFETEVTGYAQPTAVKG------------------WDKLRV--------

Cau_cresents_NP_419079 G---VKENPLPWLQS-------------------------------------LLSGVEHANFFEARATEYSKAATKGQWHG-SDGVWTSFDEMMKKRSAETIPAE-----

Cau_sp_YP_001686327 G---IKENPLPWLQS-------------------------------------LLSGVEHANFFEARATEYSKAATTGQWHG-AEGVWSSFDDMLQKRAQNTLPAE-----

Neo_sennetsu_YP_506404 E---QKVNPLLWLEAV-------------------------------------LNTVEHTNFFENKATEYSKAATKG-----------------TWDEAFNFSDA-----

Ori_tsutsugamushi_YP_001248105 L---IEQNPLTWLDQ-------------------------------------ILNGVEHTNFFENRVTEYTRSATTG-----------SWDEVFAKIDNNNLPTG-----

Ric_rickettsii_YP_001494766 L---VNHNPLPWLDEM-------------------------------------LNGVEHTNFFENRTTEYSKAATTG--------------SWEEVFTEMDATAEAK---

Wol_pipientis_YP_001974856 N---VKDNPIPWLDEI-------------------------------------LNGVEHTNFFENRVTEYSRAATQG-------------------TWEEAFAKNYTKSE

Wol_sp_NP_966023 D---VNDNPIPWLDEI-------------------------------------LNGVEHTNFFENRVTEYSRAATQG-----------------TWEEAFTENDTNNK--

Bau_cicadellinicola_YP_588829 A---TRSNPIPWINS------------------------------------WLVSDNVQVAPQEVEVSSYLVGQI------------NSEVN------SDDFSDFQL---

Esc_coli_YP_001731173 Q---TRSNPIPWINT------------------------------------WLVSDNVQVAPQEVEVSSYLVGQI------------DSEV------DTDDLSNFQL---

Shi_dysenteriae_YP_403993 Q---TRSNPIPWINT------------------------------------WLVSDNVQVAPQEVEVSSYLVGQI------------DSEV------DTDDLSNFQL---

Sod_glossinidius_YP_455265 A---TRSNPIPWINS------------------------------------WLVSDNVQVAPQEVEVSSYLVGQI------------DAEVN------SDDLSDFQL---

Yer_pestis_A_ZP_04509308 S---TRTNPIPWINS------------------------------------WLVSDNVQVAPQEVEVSSYLVGQI------------DSEVS------ADDLSDFEL---

Buc_aphidicola_NP_240009 K---KQSNPIPWIND------------------------------------WLTSDNIQIAPQETEISSYLVGQI------------DSEVS------DNEFKKFEL---

Yer_pestis_A_ZP_04512133 P--EIKDDPLVWMNK------------------------------------WLLSDTLQIAPQEAEQSNYLVGQID-----------SAVDR-----SGLSQFADL----

Halom_mukohataei_ZP_03875489 EPDEDLLEETRWFDED-------------------------------------IMMTKDNDFFSKRSTTYNKHAQSVT-----------------------AEDMF----

Natro_pharaonis_YP_327710 EPDDGLLEETRWFDED-------------------------------------IMMTKDNDFFSKRSTTYNKHAQSVT-----------------------AEDMF----

Halor_lacusprofundi_YP_002564382 ETDDDLLDETRWFDED-------------------------------------IMMTKDNDFFSKRSTTYNKHTQSVT-----------------------AKEMF----

. . *

Högbom 2010 positions || 29 30

Högbom 2010 exceptions || |||| *

Chl_muridarum_NP_296594 H----TKNPFPWMSE-------------------------------------TIDLNKEKNFFETRVTEYQHAASLTW--------------------------------

Chl_trachomatis_YP_328659 H----TKNPFPWMSE-------------------------------------TIDLNKEKNFFETRVIEYQHAASLTW--------------------------------

Halob_sp_NP_280997 G----TDNPFPWMTEQ-------------------------------------VDLNTETNFFERNVSEYQQGGSLDWD-------------------------------

Halog_borinquense_ZP_04000565 G----TDNPFPWMSEA-------------------------------------VDLNKEKNFFETQVTEYQSGGSLDW--------------------------------

Halom_utahensis_YP_003131236 G----TENPFPWMSEQ-------------------------------------VDLNKEKNFFETQVTEYQSGGSLEW--------------------------------

Natri_magadii_ZP_03692956 G----TDNPFPWLSEQ-------------------------------------VDLNKEKNFFETQVTEYQSGGSLDW--------------------------------

*.*****::* :***.*.**** .* *** ..**

Högbom 2010 positions || 29 30

Högbom 2010 exceptions || |||| *

Geo_kaustophilus_YP_148624 N---LRKKQISRTKR-----------------------------------------VDVVEGLEKTAAES----------------------------------------

Geo_sp_ZP_03557705 N---IRKKQISRAKR-----------------------------------------VDSIEELEKTVAES----------------------------------------

Myc_avium_NP_962606 PL-AEIDLDYTPVQL--------------------------------------EDTFADEDARALAAV------------------------------------------

Myc_bovis_NP_853903 PV-AEIDVDYSPAQL--------------------------------------EDTFADEDRRTLAAASA----------------------------------------

Myc_tuberculosis_NP_214747 PV-AEIDVDYSPAQL--------------------------------------EDTFADEDRRTLAAASA----------------------------------------

Nat_pharaonis_YP_331256 A---------------------------------------------------------------KSVPDLDSLTSLDG--------------------------------

Nat_pharaonis_YP_330945 D--------------------------------------------------------ASIPDVETLVTLSEDSTAAD---------------------------------

Sul_islandicus_YP_002913609 K--------LEMLLP------------------------------------------KDLDVIESW--------------------------------------------

Sul_solfataricus_NP_343843 K--------LEMLLP------------------------------------------KDLDVIESW--------------------------------------------
