# Supplementary material for: A Novel Clade of Unique Eukaryotic Ribonucleotide Reductase R2 Subunits is Exclusive to Apicomplexan Parasites
Source: J Mol Evol. 2013 Sep 18;77(3):92–106. doi: 10.1007/s00239-013-9583-y (PMC3824934; doi:10.1007/s00239-013-9583-y)
Supplement: Supplementary file 2 — Supplementary material 2 (PDF 1643 kb) [file 239_2013_9583_MOESM2_ESM.pdf]

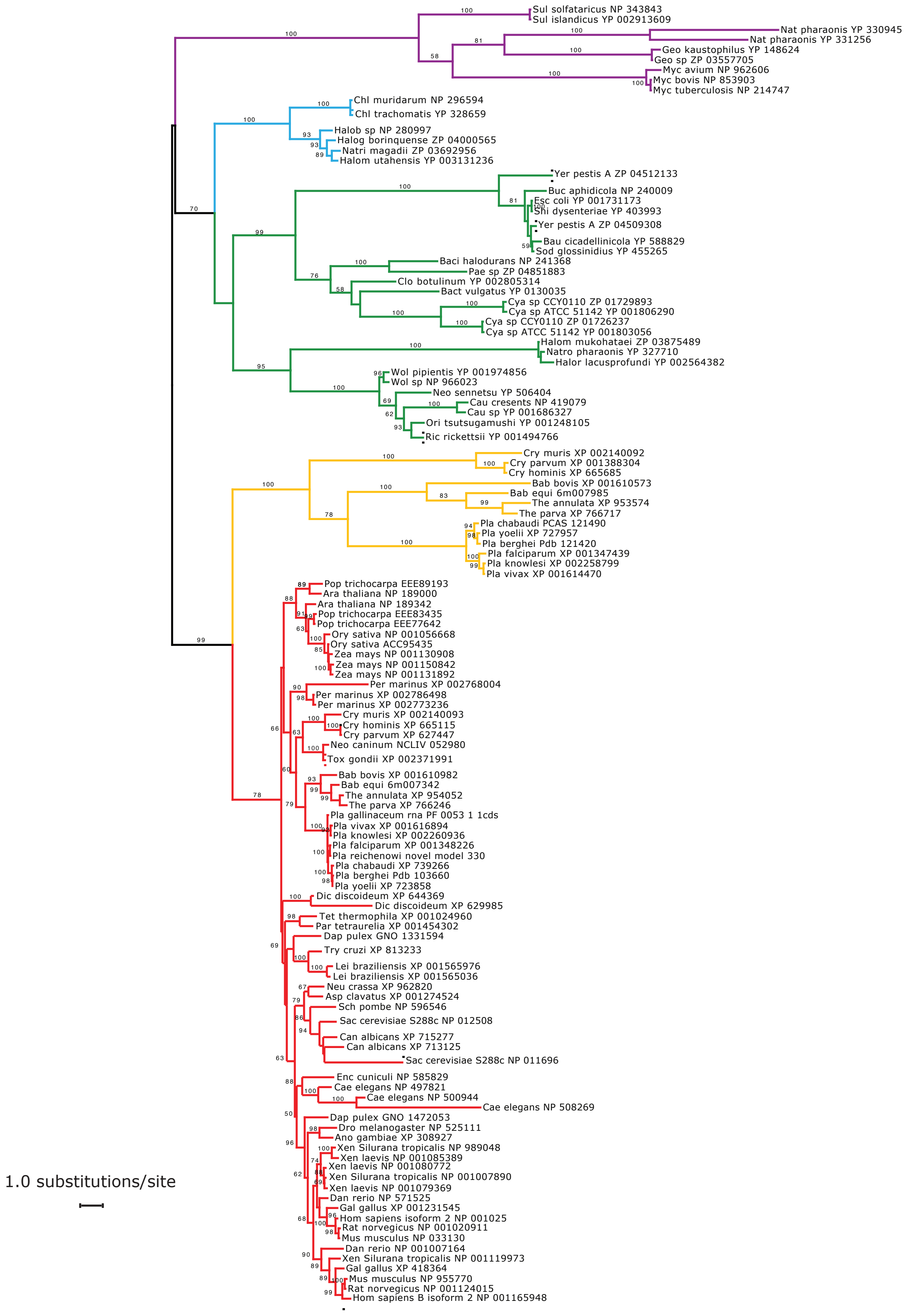

Figure S2. Maximum likelihood analysis with RAxML (seed 12345).

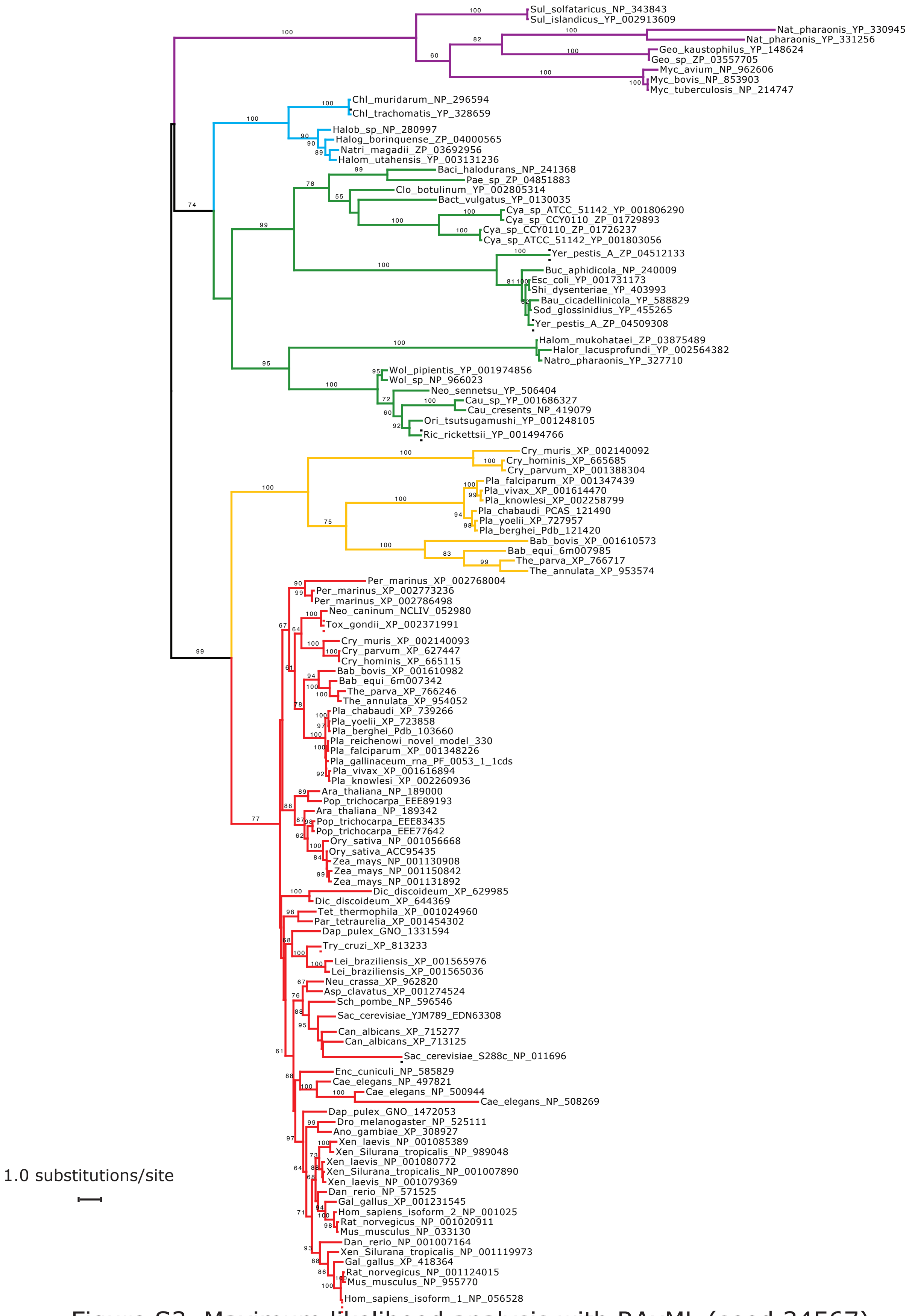

Figure S3. Maximum likelihood analysis with RAxML (seed 34567).

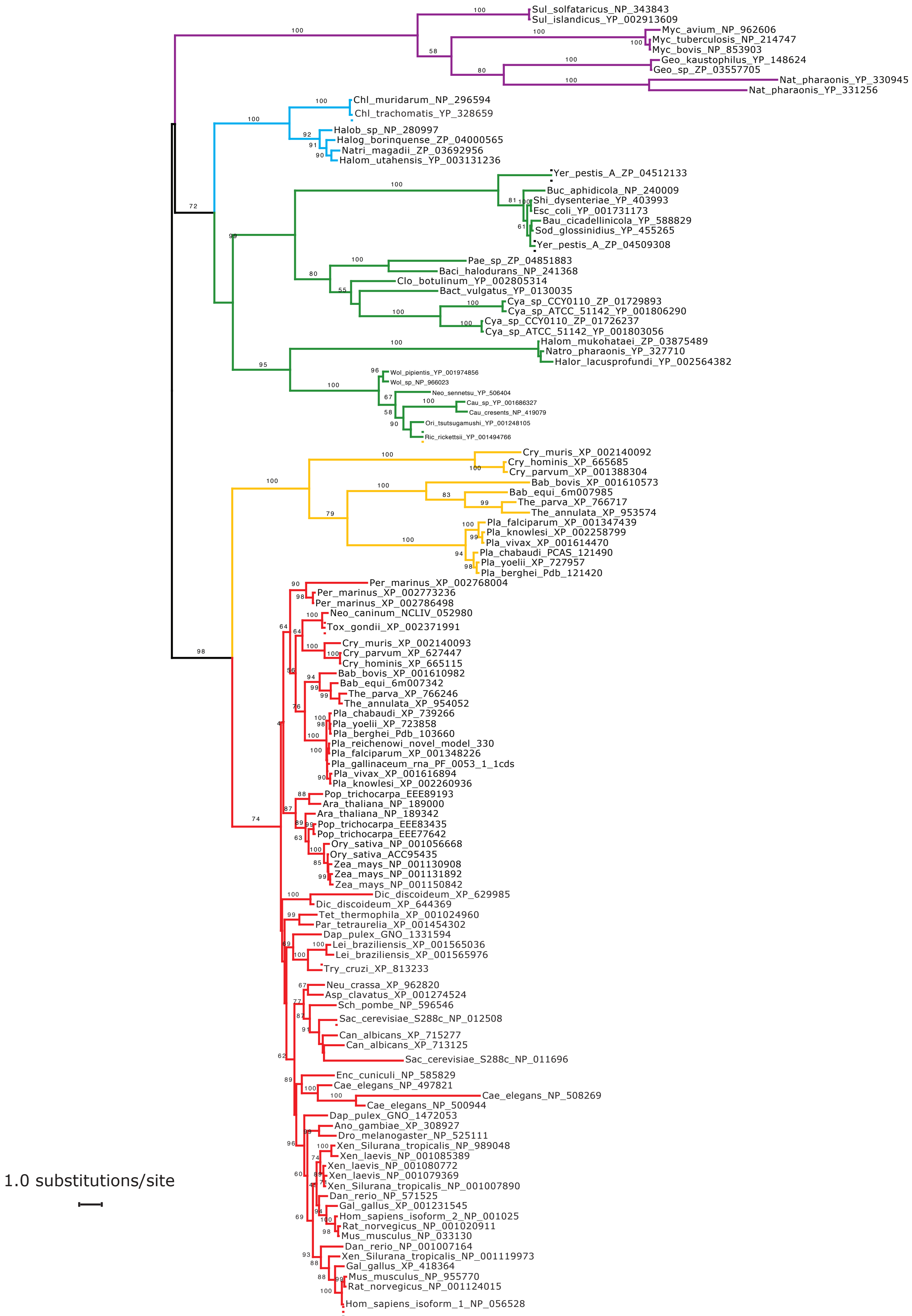

Figure S4. Maximum likelihood analysis with RAxML (seed 45678).

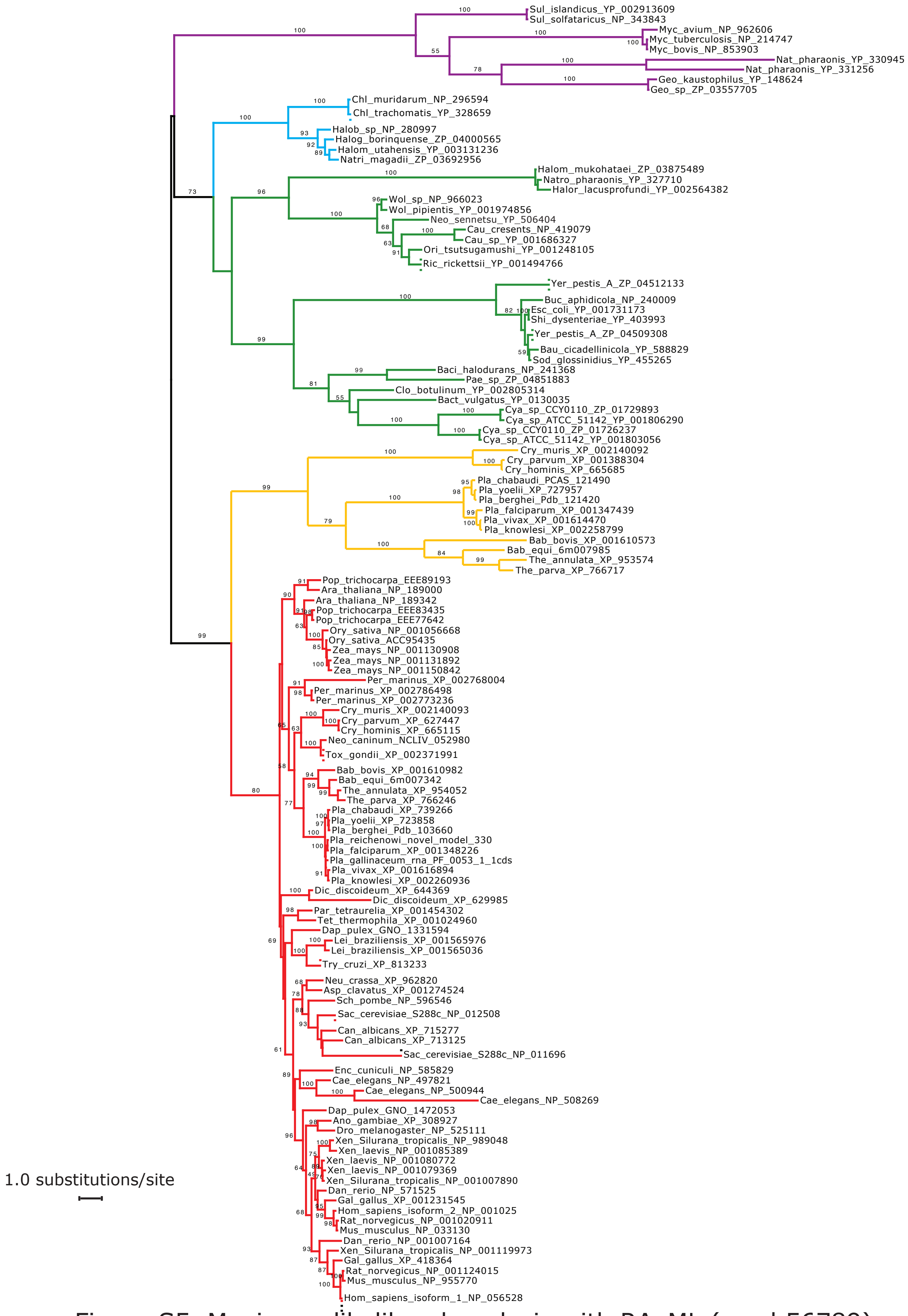

Figure S5. Maximum likelihood analysis with RAxML (seed 56789).

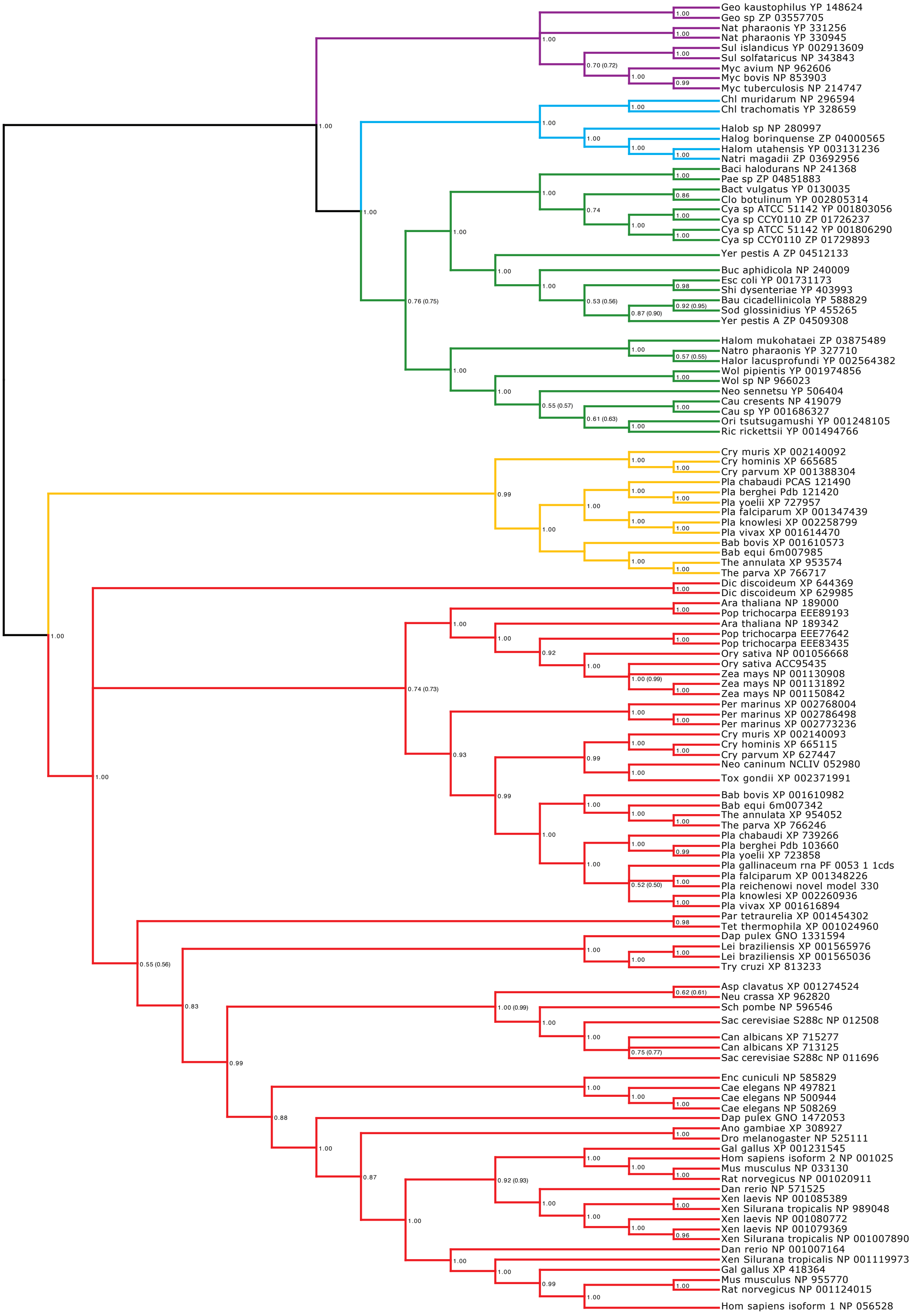

Figure S6. 50% majority rule tree of the 3.5 and 5 million generation MrBayes analyses.
