# Supplementary material for: A Novel Clade of Unique Eukaryotic Ribonucleotide Reductase R2 Subunits is Exclusive to Apicomplexan Parasites
Source: J Mol Evol. 2013 Sep 18;77(3):92–106. doi: 10.1007/s00239-013-9583-y (PMC3824934; doi:10.1007/s00239-013-9583-y)
Supplement: Supplementary file 3 — Supplementary material 3 (PDF 208 kb) [file 239_2013_9583_MOESM3_ESM.pdf]

Table S1. The standard class Ia R2 (R2_e1, R2_e2, and R2_ab), class Ic (R2c), and R2lox homolog protein taxa/proteins sampled, their unique identifier (NCBI, Eukaryotic Pathogen Database Resources, etc.), and matching RCSB Protein Data Bank structures.

Apicomplexan-specific R2 (R2_e2): [1]

Taxon	Accession #	RCSB PDB Match
Babesia bovis NrdB2	XP_001610573	2UW2
Babesia equi	6.m007985	1JK0 chain A
Cryptosporidium hominis TU502	XP_665685	1JK0 chain B
Cryptosporidium muris RN66	XP_002140092	1JK0 chain A
Cryptosporidium parvum Iowa II	XP_001388304	1JK0 chain B
Plasmodium berghei [2]	PBANKA_121420	3HF1
Plasmodium chabaudi [3]	PCHAS_121490	3HF1
Plasmodium falciparum 3D7 NrdB2	XP_001347439	2UW2
Plasmodium knowlesi strn. H NrdB?	XP_002258799	2UW2
Plasmodium vivax NrdB?	XP_001614470	2UW2
Plasmodium yoelii yoelii str. 17XNL NrdB2	XP_727957	2VUX
Theileria annulata strn. Ankara NrdB2?	XP_953574	1H0N
Theileria parva NrdB2	XP_766717	1H0N

Archaeal Standard R2 (R2_ab):

Taxon	Accession #	RCSB PDB Match
Halorubrum lacusprofundi ATCC 49239 NrdB	YP_002564382	no unique match
Halomicrobium mukohataei DSM 12286 NrdB	ZP_03875489	no unique match
Natronomonas pharaonis DSM 216 NrdB	YP_327710	no unique match

Bacterial Standard R2 (R2_ab):

Taxon	Accession #	RCSB PDB Match
Bacillus halodurans C-125 NrdB	NP_241368	2RCC
Bacteroides vulgatus ATCC 8482 NrdB	YP_0130035	no unique match
Baumannia cicadellinicola str. HC NrdB	YP_588829	no unique match
Buchnera aphidicola str. APS NrdB	NP_240009	no unique match
Caulobacter cresents CB15 NrdB	NP_419079	no unique match
Caulobacter sp. K31 NrdB	YP_001686327	no unique match
Clostridium botulinum A2 str. Kyoto NrdB	YP_002805314	no unique match
Cyanothece sp. ATCC 51142 NrdB1	YP_001803056	no unique match
Cyanothece sp. ATCC 51142 NrdB2	YP_001806290	no unique match
Cyanothece sp. CCY0110 NrdB1	ZP_01726237	no unique match
Cyanothece sp. CCY0110 NrdB2	ZP_01729893	no unique match
Escherichia coli str. K-12 substr. DH10B NrdB [4]	YP_001731173	1AV8, 2ALX, 1MXR, 1JPR, 1JQC, 1MXR
Neorickettsia sennetsu str. Miyayama NrdB	YP_506404	no unique match
Orientia tsutsugamushi Boryong NrdB	YP_001248105	no unique match
Paenibacillus sp. oral taxon 786 str. D14	ZP_04851883	no unique match
Rickettsia rickettsii str. 'Sheila Smith' NrdB [5]	YP_001494766	no unique match
Shigella dysenteriae Sd197 NrdB	YP_403993	1AV8, 2ALX, 1MXR, 1JPR, 1JQC, 1MXR
Sodalis glossinidius str. 'morsitans' NrdB	YP_455265	no unique match
Wolbachia pipientis NrdB	YP_001974856	no unique match
Wolbachia endosymbiont of *D. melanogaster* NrdB [6]	NP_966023	no unique match
Yersinia pestis Pestoides A NrdB1	ZP_04512133	no unique match
Yersinia pestis Pestoides A NrdB2	ZP_04509308	no unique match

Eukaryote Standard R2 (R2_e1):

**Taxon**	**Accession #**	**RCSB PDB Match**
Anopheles gambiae NrdB	XP_308927	no unique match
Arabidopsis thaliana NrdB1 (AtR2A)	NP_189000	no unique match
Arabidopsis thaliana NrdB2 (AtTSO2)	NP_189342	no unique match
Aspergillus clavatus NRRL 1 NrdB	XP_001274524	no unique match
Babesia bovis NrdB1	XP_001610982	no unique match
Babesia equi	6.m007342	no unique match
Caenorhabditis elegans NrdB	NP_497821	no unique match
Caenorhabditis elegans NrdB1	NP_500944	no unique match
Caenorhabditis elegans NrdB2	NP_508269	no unique match
Candida albicans NrdB1 [7]	XP_715277	no unique match
Candida albicans NrdB2 [8]	XP_713125	no unique match
Cryptosporidium hominis TU502 NrdB	XP_665115	no unique match
Cryptosporidium muris RN66 NrdB	XP_002140093	no unique match
Cryptosporidium parvum Iowa II NrdB1	XP_627447	no unique match
Danio rerio NrdB1	NP_571525	no unique match
Danio rerio NrdB2	NP_001007164	no unique match
Daphnia pulex	GNO_1472053	no unique match
Daphnia pulex	GNO_1331594	no unique match
Dictyostelium discoideum AX4 NrdB [9]	XP_644369	no unique match
Dictyostelium discoideum AX4 NrdB?	XP_629985	no unique match
Drosophila melanogaster NrdB	NP_525111	no unique match
Encephalitozoon cuniculi GB-M1 NrdB	NP_585829	no unique match
Gallus gallus NrdB1 [10]	XP_001231545	no unique match
Gallus gallus NrdB2	XP_418364	no unique match
Homo sapiens subunit M2 isoform 2 NrdB1 (R2)	NP_001025	2VUX
Homo sapiens subunit M2 isoform 1 NrdB2 (p53R2)	NP_056528	3HF1
Leishmania braziliensis NrdB1	XP_001565036	no unique match
Leishmania braziliensis NrdB2	XP_001565976	no unique match
Mus musculus NrdB1 [11]	NP_033130	1H0N
Mus musculus NrdB2	NP_955770	no unique match
Neospora caninum	NCLIV_052980	no unique match
Neurospora crassa OR74A NrdB	XP_962820	no unique match
Oryza sativa (japonica cultivar-group) NrdB1	NP_001056668	no unique match
Oryza sativa (japonica cultivar-group) NrdB2	ACC95435	no unique match
Paramecium tetraurelia NrdB [12]	XP_001454302	no unique match
Perkinsus marinus 1	XP_002786498	no unique match
Perkinsus marinus 2	XP_002773236	no unique match
Perkinsus marinus 3	XP_002768004	no unique match
Plasmodium berghei NrdB1pep	PBANKA_103660	no unique match
Plasmodium chabaudi chabaudi NrdB [13]	XP_739266	no unique match
Plasmodium falciparum 3D7 NrdB1 [14]	XP_001348226	no unique match
Plasmodium gallinaceum	rna_PF_0053.1.1.cds	no unique match
Plasmodium knowlesi NrdB	XP_002260936	no unique match
Plasmodium reichenowi	novel_model_330	no unique match
Plasmodium vivax NrdB	XP_001616894	2O1Z
Plasmodium yoelii NrdB1	XP_723858	2P1I

Eukaryote Standard R2 (R2_e1): (continued)

Taxon	Accession #	RCSB PDB Match
Populus trichocarpa NrdB1	EEE89193	no unique match
Populus trichocarpa NrdB2	EEE77642	no unique match
Populus trichocarpa NrdB3	EEE83435	no unique match
Rattus norvegicus NrdB1	NP_001020911	no unique match
Rattus norvegicus NrdB2	NP_001124015	no unique match
Saccharomyces cerevisiae S288c NrdB1 (Y2)	NP_012508	1JK0 chain A & 1SMQ
Saccharomyces cerevisiae S288c NrdB2 (Y4)	NP_011696	1JK0 chain B & 1SMS
Schizosaccharomyces pombe NrdB	NP_596546	no unique match
Tetrahymena thermophila NrdB	XP_001024960	no unique match
Theileria annulata strn. Ankara NrdB1	XP_954052	no unique match
Theileria parva NrdB1	XP_766246	no unique match
Toxoplasma gondii ME49 NrdB [15]	XP_002371991	no unique match
Trypanosoma cruzi strn. CL Brener NrdB1	XP_813233	no unique match
Xenopus laevis NrdB1	NP_001085389	no unique match
Xenopus laevis NrdB2	NP_001079369	no unique match
Xenopus laevis NrdB	NP_001080772	no unique match
Xenopus (*Silurana*) *tropicalis* NrdB1	NP_001007890	no unique match
Xenopus (*Silurana*) *tropicalis* NrdB2	NP_989048	no unique match
Xenopus (*Silurana*) *tropicalis* NrdB3	NP_001119973	no unique match
Zea mays NrdBiso1	NP_001130908	no unique match
Zea mays NrdBa	NP_001131892	no unique match
Zea mays NrdBb	NP_001150842	no unique match

Archaeal R2c:

Taxon	Accession #	RCSB PDB Match
Halobacterium sp. NRC-1 NrdB	NP_280997	no unique match
Halogeometricum borinquense DSM 11551 NrdB	ZP_04000565	no unique match
Halomicrobium utahensis DSM 12940 NrdB	YP_003131236	no unique match
Natrialba magadii ATCC 43099 NrdB	ZP_03692956	no unique match

Bacterial R2c:

Taxon	Accession #	RCSB PDB Match
Chlamydia muridarum Nigg NrdB	NP_296594	no unique match
Chlamydia trachomatis AHAR NrdB [16]	YP_328659	1SYY & 2ANI

Archaeal R2lox:

Taxon	Accession #	RCSB PDB Match
Natronomonas pharaonis DSM 2160 1	YP_331256	no unique match
Natronomonas pharaonis DSM 2160 2	YP_330945	no unique match
Sulfolobus islandicus M.16.4	YP_002913609	no unique match
Sulfolobus solfataricus P2	NP_343843	no unique match

Bacterial R2lox:

Taxon	Accession #	RCSB PDB Match
Geobacillus kaustophilus HTA426	YP_148624	no unique match
Geobacillus sp. Y412MC61	ZP_03557705	no unique match
Mycobacterium avium subsp. *paratuberculosis* K-10	NP_962606	no unique match
Mycobacterium bovis AF2122/97	NP_853903	no unique match
Mycobacterium tuberculosis H37Rv	NP_214747	3EE4

Notes:

1. While the "best matching" structures are listed, there were no suitable RCSB Protein Data Bank matches for the unorthodox apicomplexan R2_e2.
2. XP_676239 (RNRdb) was similar, but lacks first ~15 residues.
3. XP_742789 was missing ~125 first AAs, otherwise identical to PCHAS_121490.
4. Sequences YP_001731173 (*Esc. coli* NrdB) and YP_403993 (*Shi. dysenteriae* NrdB) were identical.
5. ABV76258 (RNRdb) was identical.
6. AAS13957 (RNRdb) was identical.
7. XP_715342 (RNRdb) was identical.
8. XP_713171 (RNRdb) was identical.
9. XP_645045 (RNRdb) was identical.
10. XP_419948 (RNRdb) was similar, but 8 missing AAs.
11. Not one PDB structure matched this — NP_033130 was missing 12 residues.
12. XP_001462342 (RNRdb) was identical.
13. CAH80811 (RNRdb) was identical.
14. Sequences XP_001348226 (*P. falciparum*) and novel model 330 (*P. reichenowi*) were identical and the Broad isolates PFHG00441H (*P. falciparum* HB3 nucleus) and PFMG01923I (*P. falciparum* IGH-CR14 nucleus) were identical to 3D7
15. EEE27410 (RNRdb) was identical.
16. *C. trachomatis* sequences differed from 1SYY by 1 residue

Table S2. RCSB Protein Data Bank structures used to create a structure-based alignment template.

RCSB PDB ID	Organism	Represented in Matrix	Reference
1AV8_A	*E. coli*	-	(Tong et al., 1998)
1H0N_A	*Mus musculus*	-	(Strand et al., 2002)
1JK0_A	*S. cerevisiae*	yes	(Voegtli et al., 2001)
1JK0_B	*S. cerevisiae*	yes	(Voegtli et al., 2001)
1JPR_A	*E. coli*	-	(Högbom et al., 2001)
1JQC_A	*E. coli*	-	(Högbom et al., 2001)
1MXR_A	*E. coli*	-	(Högbom et al., 2003)
1SMQ_A	*S. cerevisiae*	-	(Sommerhalter et al., 2004)
1SMS_A	*S. cerevisiae*	-	(Sommerhalter et al., 2004)
1SYY_A	*C. trachmatis*	yes	(Högbom et al., 2004)
2ALX_A	*E. coli*	yes	(Sommerhalter et al., 2005)
2ANI_A	*C. trachmatis*	yes	(Voevodskaya et al., 2007)
2O1Z_A	*P. vivax*	yes	none
2P1I_A	*P. yoelii*	yes	none
2RCC_A	*B. halodurans*	yes	none
2UW2_A	*H. sapiens*	yes	none
2VUX_A	*H. sapiens*	yes	none
3EE4_A	*M. tuberculosis*	yes	(Andersson and Högbom, 2009)
3HF1_A	*H. sapiens*	yes	(Smith et al., 2009)

The 19 "best matching" RCSB Protein Data Bank structures as determined by BLAST searches for each of the 121 taxa in the matrix. These structures were used in STRAP to create an α-carbon-based template alignment with CE, after which, ClustalW2 used this template to align the 121 sequences.

Notes:
1AV8 - chains A & B identical so B removed
1H0N - one chain only
1JK0 - chains A & B different and retained
1JPR - chains A & B identical so B removed
1JQC - chains A & B identical so B removed
1MXR - chains A & B identical so B removed
1SMQ - chains A-D identical so B-D removed
1SMS - chains A & B identical so B removed
1SYY - one chain only
2ALX - one chain only
2ANI - one chain only
2O1Z - chains A & B identical so B removed
2P1I - chains A-H identical so B-H removed
2RCC - chains A-C identical so B-C removed
2UW2 - one chain only
2VUX - chains A & B identical so B removed
3EE4 - one chain only
3HF1 - chains A & B identical so B removed

Table S3. Sequence consistency and conservation across the five clades.

H_X	***ScY2_X***	***M_X***	**R2lox**	**R2c**	**R2_ab**	**R2_e1**	**R2_e2**
N/A	85-90	364-369	absent	absent	absent	**R F V x F P**	R W V x F P
1	108	390	**W**	**W**	**W**	**W**	**W**
N/A	110	392	**P**	**P**	P	-	-
2	118	404	**D**	**D**	**D**	**D**	**D**
N/A	130	424	E	**E**	E	E	-
3	141	435	**F**	**F**	-	F	-
4	144	438	**G**	-	-	-	-
5	145	439	**E**	**E**	**D**	**D**	**D**
6	148	483	**V**	-	-	V	-
7	154	489	**P**	-	-	-	-
8	175	528	**E**	**E**	-	-	-
9	176	529	**E**	**E**	**E**	**E**	**E**
10	178	531	**K**	-	-	-	-
11	179	532	**H**	**H**	**H**	**H**	**H**
12	183	538	**F**	**F**	**Y**	**Y**	**Y**/F
13	213	589	-	**K**	-	K	K
14	235	645	**Y**	-	-	-	-
15	239	663	**E**	**E**	**E**	**E**	S>I>M
16	240	664	**G**	**G**	G	G	K
17	243	667	**A**	**F**	**F**	**F**	**F**
18	244	668	-	**Y**	Y	-	-
19	247	672	**Y**	**F**	**F**	**F**	F>L>H>I
N/A	261	697	**G**	G	G	G	
20	271	707	-	R	**R**	**R**	**R**
21	272	708	**D**	**D**	**D**	**D**	**D**
22	273	709	**E**	**E**	**E**	**E**	**E**
23	275	711	**R**	-	-	-	-
24	276	712	**H**	**H**	**H**	**H**	**H** / Q
25	280	716	**G**	**G**	-	-	-
N/A	N/A	726	-	**E**	-	-	-
26	307	755	-	**E**	E	E	E
27	310	758	-	**Y**	-	-	-
N/A	321	773	-	**G**	G	G	-
N/A	330	792	Y	**Y**	Y	Y	-
N/A	337	799	-	**R**	-	R	-
28	338	800	**R**	**R**	R	-	-
N/A	352	818	-	**N**	N	N	-
N/A	353	819	-	**P**	P	P	P
29	367-370	871-874	absent	**N F F E**	- F F E	N F F E	absent
30	376	880	absent	**Y**	**Y**	**Y**	absent
N/A	392-399	913-920	absent	absent	present?	present	present

The predominant residue(s) found in the 40 alignment positions referenced in Högbom (Högbom, 2010) for the five clades R2lox, R2c, R2_ab, R2_e1, and R2_e2. Residue(s) previously noted in these positions (thus, expected), are shown in bold font, "-" and "x" signifies inconsistency/variation in residue. *H_X* = Högbom positions (Högbom, 2010); *ScY2_X* = *S. cerevisiae* Y2 coordinates (Voegtli et al., 2001); *M_X* = matrix position, where "X" represents the

alignment coordinate in the respective study. (NB: while *S. cerevisiae* coordinates ignore gaps, matrix positions include gaps).

H_X N/A, ScY2_85-90, M_364-369

Position *ScY2_85-90* defined loop resides (Sommerhalter et al., 2005). The RFVxFP / RWVxFP motif was limited in distribution to R2_e1 and R2_e2, respectively. While RFVxFP, was consistent across R2_e1 taxa, notable exceptions occurred with substitution of the first phenylalanine by threonine (*S. cerevisiae* Y2, *T. annulata* and *T. parva*), tyrosine (*L. braziliensis*, *P. marinus*, and T. *cruzi*), aspargine (*B. bovis*), and isoleucine (*B. equi*). This first phenylalanine was substituted with tyrosine in one of the R2_e1 *C. albicans* taxa and histidine in the other sequence. In the R2_e1 apicomplexans *N. caninum* and *T. gondii*, the phenylalanine was substituted by tryptophan, which was also found across all the R2_e2 taxa. Position *ScY2_85* was consistently an arginine throughout the eukaryote R2_e1 and R2_e2 taxa, and it was reported to forms an intro-monomer hydrogen bond with glutamic acid residue at start of helix αC, position *ScY2_164*, which was also consistent across R2_e1 and R2_e2.

There has been an interesting modification to the *S. cerevisiae* Y2 loop region, where Phe86 has been replaced with threonine; i.e. an RTVxFP motif. In the Y2/Y4 heterodimer, Phe35 from Y4 extends into a pocket of Y2 lined by Phe201 and His205, an action not possible with Thr86 in a Y2 R2 homodimer (Sommerhalter et al., 2005). The authors further noted that additional *Saccharomyces* species that also encode Y4 possess a threonine, serine, or histidine in this position. However, *Saccharomyces kluyveri*, which does not encode Y4, possess the conserved phenylalanine in this position. All apicomplexan R2_e2 had a tryptophan in position *ScY2_86*, while their orthodox R2_e1 copies contain a variety of residues: *Babesia* = asparagine and isoleucine, *Neospora* and *Toxoplasma* = tryptophan, and *Plasmodium* possessed the conserved phenylalanine. It would be interesting to determine if these changes in residue composition influence the putative R2_e1 and R2_e2 interaction.

H_1, ScY2_108, M_390

Tryptophan was consistent across all five clades R2c, R2lox, R2_ab, R2_e1, and R2_e2, with three exceptions, all of which were most likely due to alignment issues (*H. sapiens* B isoform 3 NP_001165949, *P. marinus* XP_002768004, and *C. elegans* NP_508269).

H_X N/A, ScY2_110, M_392

Proline was consistent across R2c and R2lox; however, proline was also consistently found across all R2_ab bacterial taxa, save *P*. sp. ZP_04851883. Proline was not found on this position in the archaeal R2_ab, R2_e1, and R2_e2 taxa, with Ala being the predominant reside in the eukaryotes.

H_2, ScY2_118, M_404

Asparagine was consistent across all five clades R2c, R2lox, R2_ab, R2_e1, and R2_e2, save for the conserved substitutions of Glu in the R2_e1 *B. bovis* and a missing residue in the orthodox *P. marinus* XP_002768004 (a probable alignment issue).

H_X N/A, ScY2_130, M_424

Glutamic acid was found to be consistent across more than just the R2c taxa; it was consistent across all R2_ab taxa and the predominant residue found in this position for the R2_e1 and R2lox taxa. However in R2_e2, this residue was entirely lacking in this position.

H_3, ScY2_141, M_435

Phenylalanine was consistent across more than just R2c and R2lox (i.e. it was not unique to R2c and R2lox); it was also consistent across R2_e1 taxa, save the *S. cereviciae* Y4 taxa and problematically aligned *C. elegans* NP_508269. It was also consistent across the R2_ab Proteobacteria (*C. cresents*, *C.* sp., *N. sennetsu*, *O. tsutsugamushi*, *R. rickettsii, W. pipientis*, and *W*. sp.). The residue was not found in this position in the R2_e1 taxa.

H_4, ScY2_144, M_438

Glycine was unique and consistent across all R2lox taxa sampled, although (Högbom, 2010) noted that there are rare exceptions.

H_5, ScY2_145, M_439

Aspartic acid was consistent across all R2_ab and R2_e2 with exception to the three archaeal R2_ab taxa (*H. mukohataei* ZP_03875489, *N. pharaonis* YP_327710, and *H. lacusprofundi* YP_002564382), which had a

conserved substitution of glutamic acid. Additional exceptions were attributed to alignment issues (i.e. *C. muris* XP_002140092, *P. marinus* XP_002768004, *C. elegans* NP_508269, and *D. discoideum* XP_629985). Similarly, with the exception of the semi-conserved substitution of serine in *C. muris* XP_002140092, all R2_e1 taxa had an aspartic acid in this position. While Högbom (Högbom, 2010) noted rare exceptions, glutamic acid was characteristic of the R2c and R2lox subunits.

H_6, ScY2_148, M_483

While valine was consistent only across all R2lox taxa, it was also found in R2c, R2_ab, R2_e2, and it was the predominant residue across Re_e1 taxa.

H_7, ScY2_154, M_489

Proline was unique and consistent across all R2lox taxa sampled, although (Högbom, 2010) noted that there are rare exceptions.

H_8, ScY2_175, M_528

Glutamic acid was consistent across R2c and R2lox with the exception of the conserved substitution of aspartic acid for the R2lox *Geobacillus* taxa. Aspartic acid was also found in the R2_e2 *Babesia equi* and the two *Theileria* taxa. Högbom (Högbom, 2010) noted rare exceptions for R2c and R2lox at this position and the conserved substitution aspartic acid for glutamic acid in the *Geobacillus* species was not unusual. The presence of Asp in the unorthodox R2_e2 taxa *B. equi* 6m007985, *T. annulata* XP_953574, and *T. parva* XP_766717 is most likely homplasious.

H_9, ScY2_176, M_529

Glutamic acid was consistent across all five clades R2c, R2lox, R2_ab, R2_e1, and R2_e2, with one exception, *D. discoideum* XP_629985, which was most likely due to alignment issues

H_10, ScY2_178, M_531

Lysine was unique and consistent across all R2lox taxa sampled, although (Högbom, 2010) noted that there are rare exceptions.

H_11, ScY2_179, M_532

Histidine was consistent across all five clades R2c, R2lox, R2_ab, R2_e1, and R2_e2, with several exceptions: the R2_e1 Y4 *S. cerevisiae* taxa and the three R2_e2 cryptosporidians, all of which had a conserved substitution of tyrosine. The conserved substitution of histidine for tyrosine in the *S. cerevisiae* Y4 was previously documented (Huang and Elledge, 1997; Wang et al., 1997). It is interesting to note that only the R2_e2 apicomplexan *Cryptosporidium* taxa share this conserved substitution.

H_12, ScY2_183, M_538

Tyrosine was consistent across R2_ab and R2_e1 taxa, while phenylalanine was conserved across the R2c and R2lox taxa. While tyrosine was the predominant residue found in this position in the R2_e2 taxa, having been found across all six species of *Plasmodium* species, it was not consistent across the remaining taxa. Phenylalanine was found in the three cryptosporidian taxa and *Babesia bovis*, while valine was found in *Babesia equi* and isoleucine was found in the two *Theileria* species.

This position is the radical-harboring residue and, by definition, all "standard R2" proteins are expected to possess a tyrosine. However, there are exceptions to this as both Phe and Leu have been documented in some 58 taxa (Lundin et al., 2009), although it should be noted that it is the opinion of Högbom (Högbom, 2010) that these cases should be considered non-standard R2 and, as such, have been misidentified. The variety of residues found in the unorthodox taxa was quite unexpected as the identity of the residue in this position is a major defining difference between the R2_ab/ R2_e1 and R2c/R2lox groups. In the case of the three *Cryptosporidium* species, tyrosine was found 3 residues downstream in the alignment and the placement of phenylalanine in this position may be incorrect. However, in the case of *Babesia bovis*, there is no indication that a residue other than phenylalanine belongs in this location. The presence of valine in the case of *Babesia equi*, and isoleucine in the case of the two *Theileria* species is even more perplexing as substitution of phenylalanine by leucine, isoleucine, and valine has been documented in R2c proteins (Högbom, 2010).

H_13, ScY2_213, M_589

Lysine was consistent across R2c taxa. However, it was also consistent across the R2_e1 taxa, found in several of the R2_ab taxa, and the predominant residue in R2_e2. Lys was absent from the R2lox taxa sampled.

H_14, ScY2_235, M_645

Tyrosine was consistent across all R2lox taxa, yet it was also found in several R2c taxa, and sparingly represented in the R2_ab, R2_e1, and Re_e2 taxa.

H_15, ScY2_239, M_663

Glutamic acid was consistent across all five clades R2c, R2lox, R2_ab, R2_e1, and R2_e2, with several exceptions in the R2_e2 apicomplexans for which serine, isoleucine, and in the sole case of *T. annulata* XP_953574, methionine, were substituted. The conserved substitution of histidine for tyrosine in the *S. cerevisiae* Y4 was previously documented (Huang and Elledge, 1997; Wang et al., 1997). It is interesting to note that only the R2_e2 apicomplexan *Cryptosporidium* taxa share this conserved substitution. The lack of residue consistency across the remaining R2_e2 taxa is of interest as it is believed to be an iron-coordinating residues involved in ligand formation (Chakrabarti et al., 1993; Högbom et al., 2004; Nordlund et al., 1990; Roshick et al., 2000; Uppsten et al., 2006; Voegtli et al., 2001).

H_16, ScY2_240, M_664

Glycine was consistent across R2c and R2lox, save *N. pharaonis* YP_330945; however it was also consistent across all the R2_e1 taxa and more than one-half of the R2_ab taxa. Lysine was unique to R2_e2. Högbom (Högbom, 2010) noted rare exceptions for R2lox at this position and the substitution in *N. pharaonis* YP_330945 was not unexpected. Glycine was not unique to R2c and R2lox (Högbom, 2010) as it was found in almost all R2_e1 and most R2_ab taxa. However, this position may prove to be diagnostic for identifying R2_e2 taxa as lysine was found across all taxa and it was restricted to these taxa only.

H_17, ScY2_243, M_667

Alanine was unique and consistent across all R2lox taxa sampled. Phenylalanine was consistent across R2_ab, R2_e1, R2_e2, and R2c with exception of a non-conserved substitution of glycine in Y4 *S. cerevisiae* and the conserved substitution of leucine in the three archaeal R2_ab taxa and about one-third of the standard bacterial R2_ab taxa. Phenylalanine is reportedly conserved across R2_e1 and R2c, with rare exceptions in R2_e1 (Högbom, 2010) such as the non-conserved substitution of glutamine as documented in *S. cerevisiae* Y4 (Huang and Elledge, 1997; Wang et al., 1997).

H_18, ScY2_244, M_668

Tyrosine was consistent across the R2c taxa; however it was also consistent across all non-Proteobacteria and archaeal R2_ab taxa. This residue was lacking in the R2lox, R2_e1, and with the exception of *T. annulata* XP_953574, the R2_e2 taxa.

H_19, ScY2_247, M_672

Tyrosine was unique and consistent across all R2lox taxa sampled. Phenylalanine was consistent across all the R2_ab, R2_e1, and R2c taxa save the conserved substitution of tyrosine in *S. cerevisiae* Y4. Phenylalanine was found in only around one-third of the R2_e2 taxa, while non-conserved substitutions of histidine, isoleucine, and leucine were found in the remaining taxa. The conserved substitution of tyrosine for phenylalanine was documented in *S. cerevisiae* Y4 (Huang and Elledge, 1997; Wang et al., 1997).

H_X N/A, ScY2_261, M_697

Glycine consistency was not limited to the R2lox taxa, and it was consistent across all R2c and R2_e1 taxa. It was also the predominant residue in the R2_ab taxa. There was no indication of a glycine in this position of the R2_e2 taxa.

H_20, ScY2_271, M_707

Arginine was consistent across all R2_e1 taxa, save *C. elegans* NP_508269 and *D. discoideum* XP_629985 (problematic alignment), and the R2_ab taxa, with the exception of non-conserved substitutions of methionine (two of the four *Cyanothece* taxa) and lysine (Archaea). However, arginine was not limited to the R2_ab and R2_e1

taxa and it was consistent across the R2c taxa sampled and most of the R2_e2 taxa. This residue was lacking in the R2lox taxa.

H_21, ScY2_272, M_708

Aspartic acid was consistent across all five clades R2c, R2lox, R2_ab, R2_e1, and R2_e2, with several exceptions including the conserved substitution of glutamic acid in the archaeal R2_ab taxa *H. mukohataei* ZP_03875489, *N. pharaonis* YP_327710, and *H. lacusprofundi* YP_002564382 and the potentially misaligned *C. elegans* NP_500944 (lysine), *C. elegans* NP_508269 (methionine), and *D. discoideum* XP_629985 (missing a residue).

H_22, ScY2_273, M_709

Glutamic acid was consistent across all five clades R2c, R2lox, R2_ab, R2_e1, and R2_e2, save the R2_e1 *S. cerevisiae* Y4 taxa (arginine), the R2_e2 apicomplexan taxa *B. bovis* XP_001610573 (valine), *B. equi* 6m007985 (glutamine), and the two *Theileria* taxa (arginine), and the problematically aligned *C. elegans* NP_500944 (proline), *C. elegans* NP_508269 (phenylalanine), and *D. discoideum* XP_629985 (missing a residue). Substitution of arginine for glutamic acid in *S. cerevisiae* Y4 was previously documented (Huang and Elledge, 1997; Wang et al., 1997) and (Högbom, 2010) also noted possible rare exceptions in this position for R2_e1. The R2_e2 *Theileria* taxa were the only other example of this substitution.

H_23, ScY2_275, M_711

Arginine was unique and consistent across all R2lox taxa sampled, although (Högbom, 2010) noted that there are rare exceptions.

H_24, ScY2_276, M_712

Histidine was consistent across all five clades R2c, R2lox, R2_ab, R2_e1, and R2_e2, save the R2_e1 *S. cerevisiae* Y4 taxon (tyrosine), while nearly one-half of the unorthodox apicomplexans *P. berghei* Pdb_121420, *P. yoelii* XP_727957, *P. chabaudi* PCHAS_121490, *C. hominis* XP_665685, *C. parvum* XP_001388304, and *C. muris* XP_002140092 had glutamine substitution, and the problematically aligned taxa *C. elegans* NP_500944 and *D. discoideum* XP_629985 were lacking a residue in this position. Substitution of histidine by tyrosine was documented previously for the R2_e1 *S. cerevisiae* Y4 (Huang and Elledge, 1997; Wang et al., 1997).

H_25, ScY2_280, M_716

Glycine was consistent across R2c and R2lox sampled, however, it was also consistent across the archaeal R2_ab taxa.

H_X N/A, ScY2_X N/A, M_726

Glutamic acid was consistent across R2c taxa at a position *M_726*, (no *H_X* or *ScY2_X* equivalent). This residue was also consistent across the R2_ab Proteobacteria and Archaea taxa, and found in some R2lox taxa. Glutamic acid was lacking in R2_e1 and R2_e2 taxa, whose assumed αF helix (*S. cerevisiae* equivalent), ends before this position in the matrix.

H_26, ScY2_307, M_755

Consistency of glutamic acid was not limited to R2c as it was also consistent across the R2_ab, R2_e1, and R2_e2 taxa. It was absent for R2lox sequences.

H_27, ScY2_310, M_758

Tyrosine was consistent only across the R2c sampled, but it was found in the R2_e1 yeast (*Candida albicans* and *Saccharomyces cerevisiae*), *Theileria* species, and *Babesia bovis*.

H_X N/A, ScY2_321, M_773

Glycine was consistent across the R2c sampled; however, the residues were also consistent across the R2_e1 and non-archaeal R2_ab taxa.

H_X N/A, ScY2_330, M_792

Tyrosine was consistent across the R2c sampled; however, the residues were also consistent across the R2_e1 and non-archaeal R2_ab taxa.

H_X N/A, ScY2_337, M_799

Arginine was consistent across the R2c sampled; however, the residues were also consistent across most of the R2_e1 taxa.

H_28, ScY2_338, M_800

Arginine was consistent across the R2c and R2lox subunits save the two *Geobacillus* species. It was also the predominant residue for the majority of R2_ab taxa, yet it was absent from the R2_e1 and R2_e2 taxa. In contrast to Högbom (Högbom, 2010), arginine was not consistent across R2c and R2lox as the bacterial R2c taxa G. *kaustophilus* YP_148624 and *G.* sp. ZP_03557705 had a substitution of aspartic acid. The structural alignment places this position firmly within the αH helix (*S. cerevisiae* equivalent) and there were no arginine residues in close proximity and no indication that manual correction is needed.

H_X N/A, ScY2_352-353, M_818-819

Consistency of asparagine and proline were not limited to the R2c taxa as they were the predominant residues in most of the R2_ab and R2_e1 taxa. Neither residue was found in these positions in the R2lox taxa.

H_29, ScY2_367-370, M_871-874

Consistency of the motif, asparagine, phenylalanine, phenylalanine, and glutamic acid was not limited to the R2c taxa sampled. It was consistent across the R2_e1 taxa, save some instances where sequences terminated prematurely and in the case of *S. cerevisiae* Y4 where there was a semi-conserved tyrosine substitution. This motif was also found in many R2_ab taxa, and was consistent across the Proteobacteria.

H_30, ScY2_376, M_880

Tyrosine was consistent across all R2_ab and R2c taxa and the few cases where there was a lack of consistency in R2_e1 were attributed to alignment issues and early sequence termination (i.e. *P. marinus* XP_002768004, *C. elegans* NP_500944, *D. discoideum* XP_629985, *T. annulata* XP_954052, and *T. parva* XP_766246). The R2_e2 and R2lox taxa lacked a tyrosine in this position. Tyrosine was not found in the R2_e2 taxa sampled. However, all *Plasmodium* possessed a tyrosine residue four positions downstream. The C-terminus lacks structure, it is flexible, and one cannot say with certainty whether or not the tyrosine residues found in this region in the R2_e2 *Plasmodium* species are homologous to those of R2_e1 and R2c. Statements regarding positional homology in this region of the alignment are tenuous.

With the potential for exceptions, Högbom (Högbom, 2010) noted positions characteristic of the - Mn/Fe proteins [*H_3*(F), *H_5*(E), *H_8*(E), and *H_12*(F)] and standard R2 proteins [*H_3*, *H_5*(D), *H_8*, and *H_12*(Y)]. With an increased sampling of R2 subunits, we found that phenylalanine at position *H_3* was not unique to R2lox and R2c, being found across most R2_e1 taxa. We also found that glutamic acid at position *H_8* was, in *Geobacillus*, substituted with a conserved aspartic acid, and this aspartic acid was also found in some of the R2_e2 taxa. The novel R2_e2 clade lacked the highly conserved phenylalanine at *H_3*(F) and glutamic acid at *H_5*, typical of Mn/Fe proteins, and while the presence of tyrosine in position *H_12* would classify the *Plasmodium* species as "standard R2", the substitution of phenylalanine in the *Crypotosporidium*, would classify them as Mn/Fe proteins. (However, classifying *Crypotosporidium* as an Mn/Fe protein, would conflict with the data in positions *H_3* and *H_5*).

Table S4. MrBayes 3.5 and 5 million generation analysis statistics.

	**3.5 million**	**5 million**
ASDFS	0.004732	0.004898
-Ln cold-chain score	-47482.79	-47479.19
	-47484.61	-47479.19
	-47482.79	-47479.79
	-47487.42	-47483.19
PSRF for tree length	1.002	1.000
PSRF for alpha	1.000	1.000
PSRF for branch lengths	1.000 to 1.044	1.000 to 1.017

ASDFS = average standard deviation of split frequencies; -Ln cold-chain score of the four runs; PSRF = the Potential Scale Reduction Factor for tree length, alpha and branch lengths.

Table S5 A. Unambiguous character states supporting the unorthodox apicomlexan R2_e2 clade using a most parsimonious ancestral state character reconstruction as implemented by the 'trace' function in MacClade.

Character (Position in Matrix)	Ancestral Character State	Character State	Character Information
266	Leu	Asn	1
379	Glu	Asn/Ser	2
408	Trp	Leu	1
421	Thr	Asp	1
424	Glu	Leu	3
425	Arg	Leu	4
436	Ala	Ileu	1
560	Glu	Lys	5
579	Ala	Lys	6
586	Ileu	Leu	1
590	Ala	Thr	1
603	Asp	Ser	1
643	Val	Ileu	4
662	Val	Ileu	7
664	Gly	Lys	8
757	Glu	Asn	1
793	Ileu	Leu	4
821	Glu	Thr	4
872	Phe	Thr	9
875	Lys	Arg	1
888	Gly	Ser	7

1. Changed above to a state convergent with a state outside this clade. State at this node is convergent with a state outside this clade.
2. Derived state unclear. State at this node is convergent with a state outside this clade.
3. Uniquely derived state but changed above to a state not found outside this clade.
4. Reversed above to a state found in ancestor. State at this node is convergent with a state outside this clade.
5. Reversed above to state found in ancestor or changed above to a state convergent with a state outside this clade. State at this node is convergent with a state outside this clade.
6. Changed above to a state convergent with a state outside this clade.
7. State at this node is convergent with a state outside this clade.
8. Uniquely derived state, unchanged above. Two or more other states found outside this clade.
9. Reversed above to state found in ancestor or changed above to a state convergent with a state outside this clade.
10. Reversed above to a state found in ancestor.

Table S5 B. Unambiguous character states supporting the eukaryotic standard R2_e1 clade using a most parsimonious ancestral state character reconstruction as implemented by the 'trace' function in MacClade.

Character (Position in Matrix)	Ancestral Character State	Character State	Character Information
265	Asn	Leu	4
365	Trp	Phe	10
399	Lys	Gln	4
426	Asp	His	4
429	Leu	Ser	6
482	Ala	Leu -> Ileu	5
494	Leu	Glu	1
538	Phe	Tyr	7
541	Ilue	Leu	1
545	Ilue	Tyr	1
576	Glu	Ileu->Leu	7
580	Leu	Ileu	1
582	Asp	Thr	1
600	Leu	Trp	8
601	Leu	Ileu	7
673	Asn	Cys	6
676	Leu	Phe	1
677	Ser	Trp	3
678	Phe	Leu	4
682	Glu	Asn->Gly	5
717	Val	Cys	10
754	Leu	Ileu	7
771	Ileu	Leu	1
772	Leu	Ileu	4
775	Ser	Asn	7
776	Lys	Ser	4
790	Lys	Val->Ser	5
823	Leu	Met	7
865	Asp	Ser	1
912	Phe	Leu	4

1. Changed above to a state convergent with a state outside this clade. State at this node is convergent with a state outside this clade.
2. Derived state unclear. State at this node is convergent with a state outside this clade.
3. Uniquely derived state but changed above to a state not found outside this clade.
4. Reversed above to a state found in ancestor. State at this node is convergent with a state outside this clade.
5. Reversed above to state found in ancestor or changed above to a state convergent with a state outside this clade. State at this node is convergent with a state outside this clade.
6. Changed above to a state convergent with a state outside this clade.
7. State at this node is convergent with a state outside this clade.
8. Uniquely derived state, unchanged above. Two or more other states found outside this clade.
9. Reversed above to state found in ancestor or changed above to a state convergent with a state outside this clade.
10. Reversed above to a state found in ancestor.

Supplemental References

Andersson, C.S., Högbom, M., 2009. A *Mycobacterium tuberculosis* ligand-binding Mn/Fe protein reveals a new cofactor in a remodeled R2-protein scaffold. Proceedings of the National Academy of Sciences of the United States of America 106, 5633-5638.

Chakrabarti, D., Schuster, S.M., Chakrabarti, R., 1993. Cloning and characterization of subunit genes of ribonucleotide reductase, a cell-cycle-regulated enzyme, from *Plasmodium falciparum*. Proceedings of the National Academy of Sciences of the United States of America 90, 12020-12024.

Högbom, M., 2010. The manganese/iron-carboxylate proteins: What is what, where are they, and what can the sequences tell us? Journal of Biological Inorganic Chemistry 15, 339-349.

Högbom, M., Andersson, M.E., Nordlund, P., 2001. Crystal structures of oxidized dinuclear manganese centres in Mn-substituted class I ribonucleotide reductase from *Escherichia coli*: Carboxylate shifts with implications for O_2 activation and radical generation. Journal of Biological Inorganic Chemistry 6, 315-323.

Högbom, M., Galander, M., Andersson, M.E., Kolberg, M., Hofbauer, W., Lassmann, G., Nordlund, P., Lendzian, F., 2003. Displacement of the tyrosyl radical cofactor in ribonucleotide reductase obtained by single-crystal high-field EPR and 1.4-Å x-ray data. Proceedings of the National Academy of Sciences of the United States of America 100, 3209-3214.

Högbom, M., Stenmark, P., Voevodskaya, N., McClarty, G., Gräslund, A., Nordlund, P., 2004. The radical site in chlamydial ribonucleotide reductase defines a new R2 subclass. Science 305, 245-248.

Huang, M., Elledge, S.J., 1997. Identification of RNR4, encoding a second essential small subunit of ribonucleotide reductase in *Saccharomyces cerevisiae*. Molecular and Cellular Biology 17, 6105-6113.

Lundin, D., Torrents, E., Poole, A.M., Sjöberg, B.-M., 2009. RNRdb, a curated database of the universal enzyme family ribonucleotide reductase, reveals a high level of misannotation in sequences deposited to GenBank. BMC Genomics 10, 589.

Nordlund, P., Sjöberg, B.-M., Eklund, H., 1990. Three-dimensional structure of the free radical protein of ribonucleotide reductase. Nature 345, 593-598.

Roshick, C., Iliffe-Lee, E.R., McClarty, G., 2000. Cloning and characterization of ribonucleotide reductase from *Chlamydia trachomatis*. Journal of Biological Chemistry 275, 38111-38119.

Smith, P., Zhou, B., Ho, N., Yuan, Y.-C., Su, L., Tsai, S.-C., Yen, Y., 2009. 2.6 A X-ray crystal structure of human p53R2, a p53-inducible ribonucleotide reductase. Biochemistry (Moscow) 48, 11134-11141.

Sommerhalter, M., Saleh, L., Bollinger, J.M., Rosenzweig, A.C., 2005. Structure of *Escherichia coli* ribonucleotide reductase R2 in space group P6122. Acta Crystallographica Section D 61, 1649-1654.

Sommerhalter, M., Voegtli, W.C., Perlstein, D.L., Ge, J., Stubbe, J., Rosenzweig, A.C., 2004. Structures of the yeast ribonucleotide reductase Rnr2 and Rnr4 homodimers. Biochemistry (Moscow) 43, 7736-7742.

Strand, K.R., Karlsen, S., Andersson, K.K., 2002. Cobalt substitution of mouse R2 ribonucleotide reductase as a model for the reactive diferrous state. Spectroscopic and structural evidence for a ferromagnetically coupled dinuclear cobalt cluster. Journal of Biological Chemistry 277, 34229-34238.

Tong, W., Burdi, D., Riggs-Gelasco, P., Chen, S., Edmondson, D., Huynh, B., Stubbe, J., Han, S., Arvai, A., Tainer, J., 1998. Characterization of Y122F R2 of *Escherichia coli* ribonucleotide reductase by time-resolved physical biochemical methods and X-ray crystallography. Biochemistry (Moscow) 37, 5840-5848.
Uppsten, M., Färnegårdh, M., Domkin, V., Uhlin, U., 2006. The first holocomplex structure of ribonucleotide reductase gives new insight into its mechanism of action. Journal of Molecular Biology 359, 365-377.
Voegtli, W.C., Ge, J., Perlstein, D.L., Stubbe, J., Rosenzweig, A.C., 2001. Structure of the yeast ribonucleotide reductase Y2Y4 heterodimer. Proceedings of the National Academy of Sciences of the United States of America 98, 10073-10078.
Voevodskaya, N., Galander, M., Högbom, M., Stenmark, P., McClarty, G., Gräslund, A., Lendzian, F., 2007. Structure of the high-valent $Fe^{III}Fe^{IV}$ state in ribonucleotide reductase (RNR) of *Chlamydia trachomatis*—combined EPR, ^{57}Fe-, ^{1}H-ENDOR and X-ray studies. Biochimica et Biophysica Acta 1774, 1254-1263.
Wang, P.J., Chabes, A., Casagrande, R., Tian, X.C., Thelander, L., Huffaker, T.C., 1997. Rnr4p, a novel ribonucleotide reductase small-subunit protein. Molecular and Cellular Biology 17, 6114-6121.
