# Supplementary material for: A Novel Clade of Unique Eukaryotic Ribonucleotide Reductase R2 Subunits is Exclusive to Apicomplexan Parasites
Source: J Mol Evol. 2013 Sep 18;77(3):92–106. doi: 10.1007/s00239-013-9583-y (PMC3824934; doi:10.1007/s00239-013-9583-y)
Supplement: Supplementary file 4 — Supplementary material 4 (DOCX 29 kb) [file 239_2013_9583_MOESM4_ESM.docx]

# Supplemental Material

## Supplemental file 1 – Fig. S1 Annotated Alignment

## Supplemental file 2 – Figs. S2-S6 Additional Phylogenetic Trees from Maximum Likelihood and MrBayes Analyses

Fig. S2 Maximum likelihood analysis with RAxML (seed 12345). 1000 rapid bootstrap inferences followed by ML search for seed #12345. Bootstrap values above 50% are shown.

**Fig. S3** Maximum likelihood analysis with RAxML (seed 34567). 1000 rapid bootstrap inferences followed by ML search for seed #34567. Bootstrap values above 50% are shown.

**Fig. S4** Maximum likelihood analysis with RAxML (seed 45678). 1000 rapid bootstrap inferences followed by ML search for seed #45678. Bootstrap values above 50% are shown.

**Fig. S5** Maximum likelihood analysis with RAxML (seed 56789). 1000 rapid bootstrap inferences followed by ML search for seed #45678. Bootstrap values above 50% are shown.

**Fig. S6** The 3.5 and 5 million generation MrBayes relationships were identical and the posterior probability values were comparable. Where posterior probability values differ, the 5 million generation posterior probability values are shown in brackets.

## Supplemental file 3 – Tables S1-S5

**Table S1** The standard class Ia R2 (R2_e1, R2_e2, and R2_ab), class Ic (R2c), and R2lox homolog protein taxa/proteins sampled, their unique identifier (NCBI, Eukaryotic Pathogen Database Resources, etc.), and matching RCSB Protein Data Bank structures.

**Table S2** RCSB Protein Data Bank structures used to create a structure-based alignment template.

**Table S3** Sequence consistency and conservation across the five clades.

**Table S4** MrBayes 3.5 and 5 million generation analysis statistics.

**Table S5a** Unambiguous character states supporting the unorthodox apicomplexan R2_e2 clade using a most parsimonious ancestral state character reconstruction as implemented by the Trace function in MacClade.

**Table S5b** Unambiguous character states supporting the eukaryotic standard R2_e1 clade using a most parsimonious ancestral state character reconstruction as implemented by the Trace function in MacClade.
